# Thiamyxins: Structure and Biosynthesis of Myxobacterial RNA‐Virus Inhibitors**

**DOI:** 10.1002/anie.202212946

**Published:** 2022-11-28

**Authors:** Patrick A. Haack, Kirsten Harmrolfs, Chantal D. Bader, Ronald Garcia, Antonia P. Gunesch, Sibylle Haid, Alexander Popoff, Alexander Voltz, Heeyoung Kim, Ralf Bartenschlager, Thomas Pietschmann, Rolf Müller

**Affiliations:** ^1^ Helmholtz-Institute for Pharmaceutical Research Saarland (HIPS) Helmholtz Centre for Infection Research (HZI) and Department of Pharmacy Saarland University Saarbrücken Germany; ^2^ German center for infection research (DZIF) Braunschweig Germany; ^3^ Institute of Experimental Virology TWINCORE Centre for Experimental and Clinical Infection Research a joint venture between the Medical School Hannover (MHH) and the Helmholtz Centre for Infection Research (HZI) Hannover Germany; ^4^ German Center for Infection Research Hannover-Braunschweig Partner Site, and Cluster of Excellence RESIST (EXC 2155) Hannover Germany; ^5^ Department of Infectious Diseases Molecular Virology Heidelberg University German Center for Infection Research Heidelberg Partner Site and Division of Virus-Associated Carcinogenesis German Cancer Research Center (DKFZ) German Center for Infection Research (DZIF) Heidelberg Germany

**Keywords:** Antiviral Agents, Biosynthesis, Depsipeptides, Natural Products, Structure Elucidation

## Abstract

During our search for novel myxobacterial natural products, we discovered the thiamyxins: thiazole‐ and thiazoline‐rich non‐ribosomal peptide‐polyketide hybrids with potent antiviral activity. We isolated four congeners of this unprecedented natural product family with the non‐cyclized thiamyxin D fused to a glycerol unit at the C‐terminus. Alongside their structure elucidation, we present a concise biosynthesis model based on biosynthetic gene cluster analysis and isotopically labelled precursor feeding. We report incorporation of a 2‐(hydroxymethyl)‐4‐methylpent‐3‐enoic acid moiety by a GCN5‐related N‐acetyltransferase‐like decarboxylase domain featuring polyketide synthase. The thiamyxins show potent inhibition of RNA viruses in cell culture models of corona, zika and dengue virus infection. Their potency up to a half maximal inhibitory concentration of 560 nM combined with milder cytotoxic effects on human cell lines indicate the potential for further development of the thiamyxins.

## Graphical abstract

The chemical structure and absolute stereochemistry of an unprecedented natural product family, the thiamyxins, is presented. These thiazole‐ and thiazoline‐containing cyclic depsipeptides are produced by a nonribosomal peptide synthetase‐polyketide synthase hybrid biosynthetic gene cluster, whose structure is described herein. The thiamyxins were found to exhibit promising antiviral activity against three different RNA viruses.

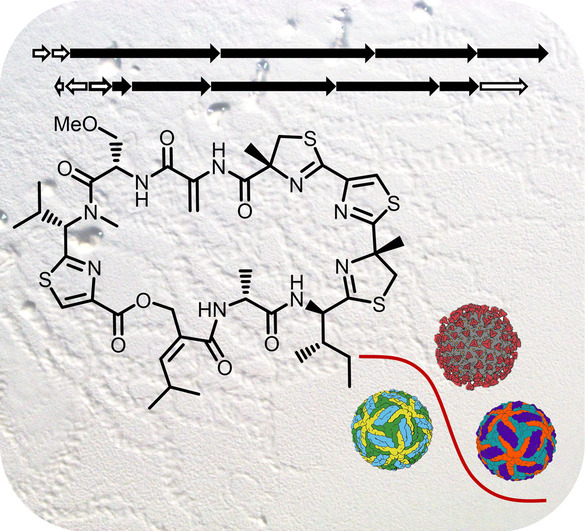

## Introduction

The severe acute respiratory syndrome coronavirus 2 (SARS‐CoV‐2) that was first identified in December 2019 in Wuhan, China, is the cause for the ongoing COVID‐19 pandemic that is challenging global health administrations in an unprecedented way.^[1]^ Due to the highly infectious nature of this pathogen, great efforts are being undertaken in developing medications to reduce and stop the spread of SARS‐CoV‐2.^[3]^ However, it is by far not the only human pathogenic RNA virus with high economic and social burden.^[4]^ Dengue fever, caused by infection with the dengue virus, on the one hand is a leading cause of severe illness and death in some Asian and Latin American countries.^[5]^ Zika virus, on the other hand was found to be linked to congenital malformations in newborns and miscarriages due to intrauterine infection of the fetus with the virus.^[6]^ Considering the recent pandemic, the possibility of future global health crises caused by viruses, but also ineffective treatment options against a variety of other viral diseases, it is of the utmost importance to keep identifying new compounds with antiviral activities as potential basis for novel drug candidates.

Bacterial natural products (NPs) represent an ubiquitous source of novel chemistry, coming with a diverse range of biological activities.^[7]^ They hereby form a great repository of finding promising lead structures in the search for new therapeutics.^[8]^ Myxobacteria are predatory Gram‐negative bacteria that have been intensely studied due to their biological uniqueness and extremely large genomes which are extraordinary rich in biosynthetic gene clusters (BGC) encoding diverse NPs.^[9]^ An important aspect of compound discovery from myxobacteria is that the taxonomic distance correlates with distance in chemical diversity, making taxonomically distant strains the most promising source for the discovery of yet undescribed NP families.^[10]^ As a result of this finding, we constantly aim to explore novel myxobacterial strains, such as *Myxococcaceae* strain MCy9487, towards their biosynthetic potential to produce biologically active NPs. In this manuscript, we report the structure of a family of unprecedented cyclic depsipeptides produced by this strain, which we called the thiamyxins, alongside the determination of their stereochemistry and biosynthesis, as well as their intriguing activities against RNA viruses.

## Results and Discussion

**Figure 1:**
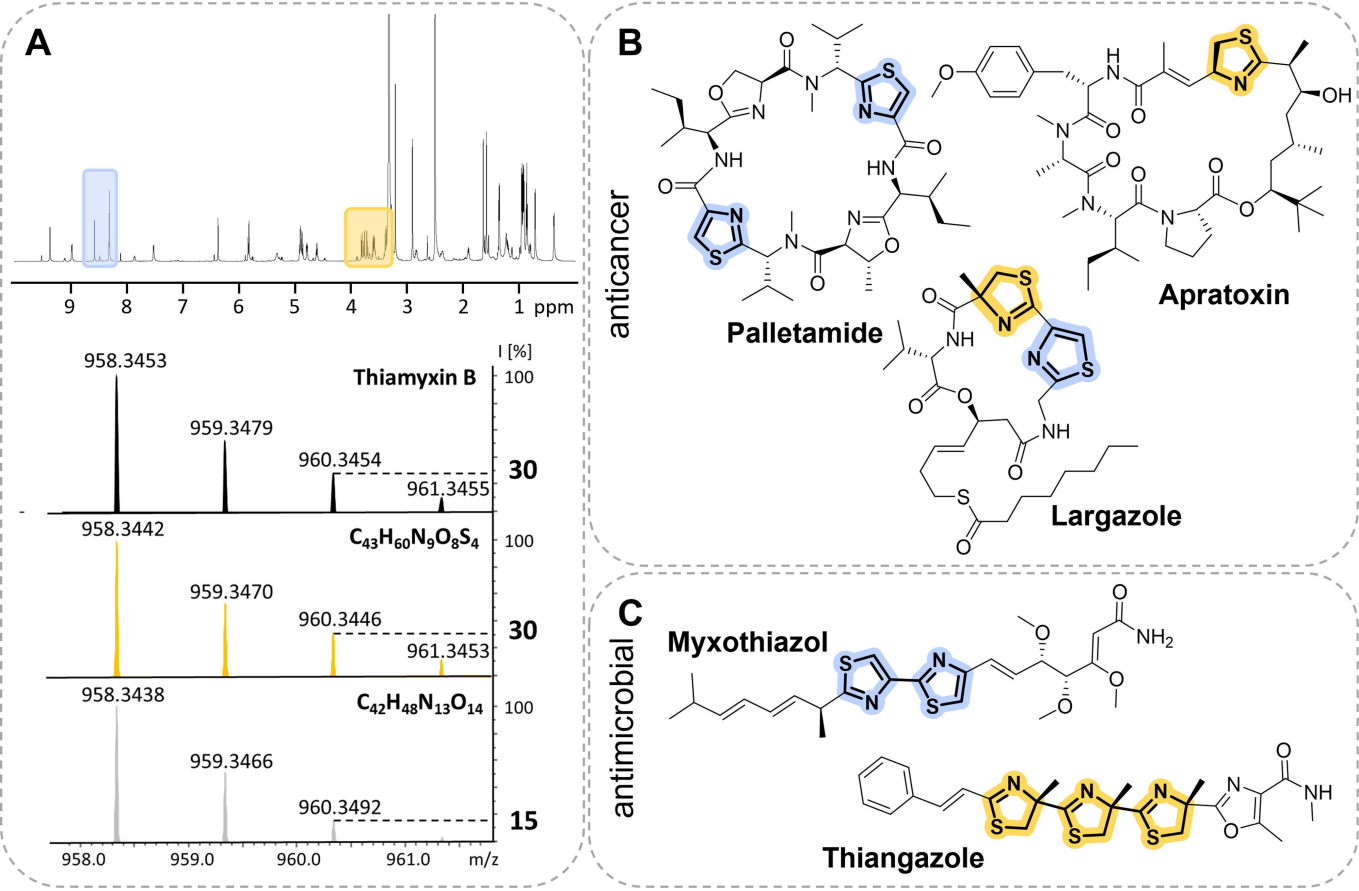
A) Characteristic chemical shifts (top, ^1^H NMR spectrum) and isotope pattern (bottom, mass spectrum) indicating the presence of thiazole and thiazoline in the thiamyxins. Mass spectra (top to bottom): measured spectrum of thiamyxin B (black), calculated spectrum for C_43_H_60_N_9_O_8_S_4_ (yellow) and C_42_H_48_N_13_O_14_ (grey) as an example for a possible sum formulae highlighting the intensity shift of the second isotope peak caused by ^34^S. B) Natural products from cyanobacteria and C) myxobacteria comprising thiazoline (yellow) and thiazole (blue) units with their most prominent biological activity.^[2]^

**Figure 2:**
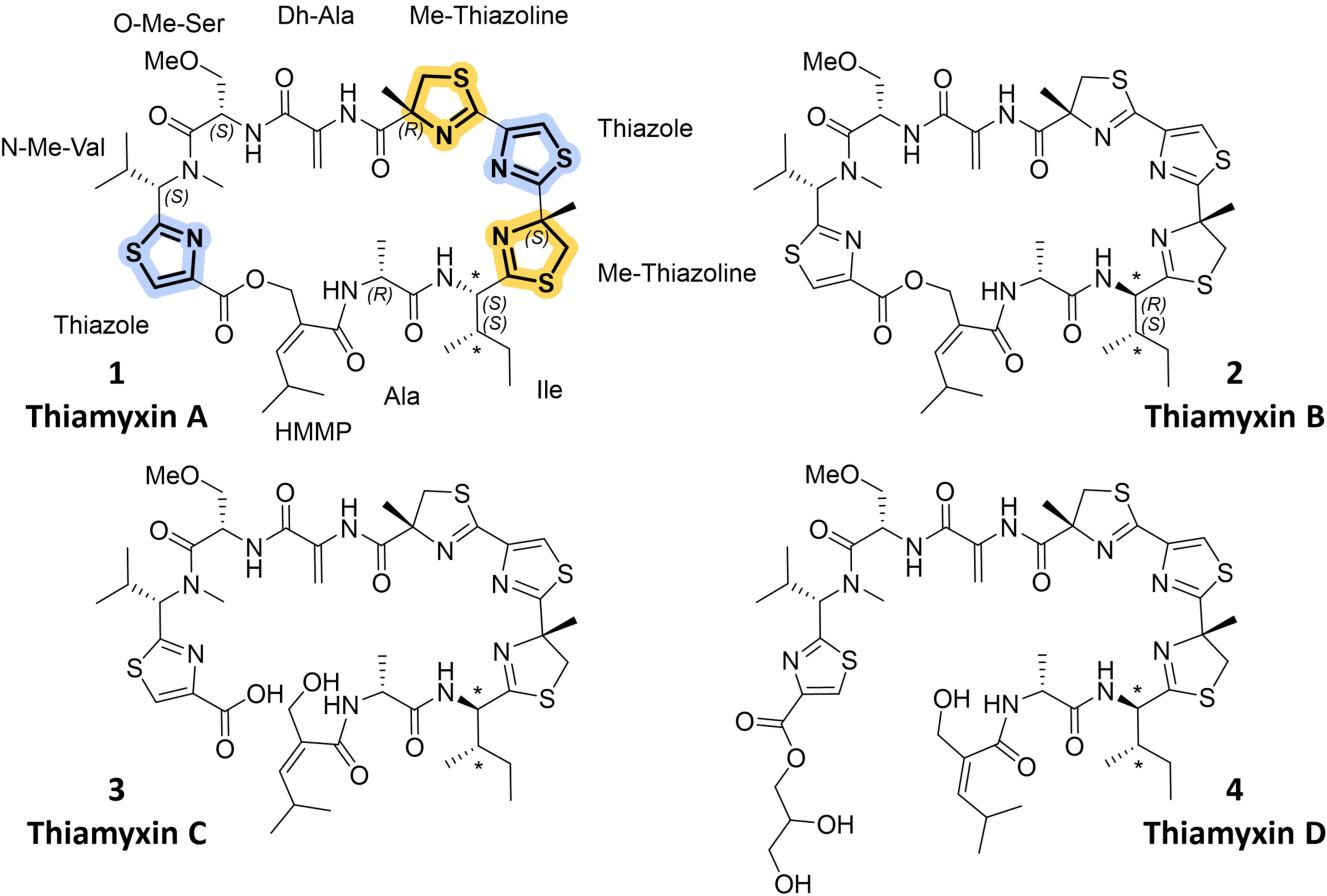
Chemical structures of the four congeners of the thiamyxin family (A–D). N‐Me‐Val: N‐Methyl‐Valine, O‐Me‐Ser: O‐Methyl‐Serine, Dh‐Ala: Dehydroalanine, Me‐Thiazoline: Methylthiazoline, Ala: Alanine, HMMP: 2‐(hydroxymethyl)‐4‐methylpent‐3‐enoic acid. *Proposed stereochemistry based on observed chemical shifts and coupling constants, prevalence of l‐Ile and d‐*allo*‐Ile, as well as the presence of an epimerization domain in module 4 and 5.

MCy9487 is a myxobacterial strain of the family *Myxococcaceae* isolated from soil of the Saarland University campus. Phylogenetic analysis based on 16S RNA revealed the strain's distinct position within the *Myxococcus‐Pyxidicoccus‐Corallococcus* clade. Due to its yet unclassified genus and the interesting inhibitory activity profile (Table S1) of the crude extract, it was prioritized for chemical in depth‐analysis. High performance liquid chromatography—high resolution mass spectrometry (HPLC‐*hr*MS) analysis of the MCy9487 crude extract and nuclear magnetic resonance spectroscopy (NMR) analysis of prefractionated crude material, revealed a family of at least four putatively novel NPs. *hr*MS isotope pattern and NMR signal shifts indicated peptide type structures with some striking characteristics including thiazole and thiazoline substructures (Figure 1A). The thiazole scaffold is a key structure in drug discovery and medicinal chemistry, as it has been correlated with a broad variety of biological activities such as antiviral, anticancer and antibacterial activities.^[11]^ This finding highly resembles observations from nature: Thiazoline and thiazole containing NPs were found to exhibit promising anticancer and antimicrobial properties as exemplified by the cyclic cyanobacterial NPs patellamide,^[2a]^ largazole^[2d]^ and apratoxin^[2e]^ or the myxobacterial NPs myxothiazol^[2b]^ and thiangazole^[2c]^ (Figure 1B and C). These examples made the thiamyxins promising targets for isolation.[^2c^, ^12^] An optimized production and purification process using liquid/liquid partitioning and semipreparative LC–MS led to the isolation of 1.5–2 mg each of four different congeners belonging to the thiamyxin family (thiamyxin A–D, Figure 2). Isolated yields were <0.2 mg L^−1^ for thiamyxin A, 0.6 mg L^−1^ for thiamyxin B, 1.4 mg L^−1^ for thiamyxin C and <0.2 mg L^−1^ for thiamyxin D. The structures of the isolated compounds were elucidated subsequently using a combination of NMR, high resolution (*hr*) MS and detailed configurational analysis, prior to evaluation of their biological activities against a broader panel of pathogens, including a panel of human pathogenic viruses.

Thiamyxin A and B were assigned a molecular formula of C_43_H_59_N_9_O_8_S_4_ based on *hr*MS data. Interpretation of the 1D and 2D NMR spectra, alongside with their characteristic MS^2^ fragmentation pattern, revealed all thiamyxins to consist of the following peptide sequence: 2‐(hydroxymethyl)‐4‐methylpent‐3‐enoic acid(HMMP)‐(Ala)‐(Ile)‐methylthiazoline(Me‐thiazoline)‐thiazole‐Me‐thiazoline‐dehydroalanine(Dh‐Ala)‐O‐methylserine(O‐Me‐Ser)‐N‐methylvaline(N‐Me‐Val)‐thiazole.

The occurrence of two thiazoline and two thiazole moieties was recognizable and eponymous for the thiamyxins (see Figure 2). Their consistent peptide sequence and exact mass but different retention time on HPLC indicate thiamyxin A **1** and B **2** to be diastereomers. Specific fragment connectivity was obtained for all derivatives by detailed analysis of homonuclear and heteronuclear 2D NMR data (see Supporting Information). The characteristic chemical shift of the HMMP methylene in **1** at δ_H_ 4.90/4.86 and δ_C_ 67.0 ppm, alongside with its HMBC correlation to the thiazole carboxy function (δ_C_ 160.5 ppm) implies cyclization between the C‐terminal thiazole and the HMMP primary alcohol. The HMBC correlation in **2** (δ_H_ 4.81/4.92 and δ_C_ 65.9 ppm to δ_C_ 160.4 ppm) is consistent with the finding for **1**. In contrary, thiamyxin C **3** shows a shielded chemical shift at this position of δ_H_ 4.00 and δ_C_ 63.1 ppm. This signal, in contrast to the corresponding signal in **1** and **2**, does not show any scalar coupling, indicating free rotatability of the HMMP methylene (Figure S1). Together with the assigned molecular formula (C_43_H_61_N_9_O_9_S_4_), thiamyxin C **3** was determined to be the ring open congener of **1** and **2**. The molecular mass of thiamyxin D **4** is further increased by C_3_H_6_O_2_ when compared to **3** and the chemical shift of the HMMP methylene only shows a deviation of 0.1 ppm for δ_C_ and an exactly matched δ_H_ compared to **3**, affirming that thiamyxin D also belongs to the open chain derivatives. Thorough analysis of the 1D and 2D NMR spectra (Tables S4–S7) finally revealed, that this derivative features an additional glycerol unit attached to the C‐terminal carboxyl function (Figure S2).

The stereochemical configuration of the individual aminoacids was elucidated using Marfey's method.^[13]^ The Ala and O‐Me‐Ser stereocenters were assigned as *R* and *S*, respectively, by comparing the hydrolyzed thiamyxins to commercially available references (Table S1). The configuration of the Me‐thiazoline moieties were assigned by comparison to thiangazole, wherein all Me‐thiazoline units previously were described to be derived from *R*‐configured 2‐Me‐cysteine.^[2c]^ Chiral HPLC retention time comparison of the derivatized hydrolysis products of thiangazole and thiamyxin B showed the methylcysteine peaks with resembling retention times (Figure S10), identifiying the Me‐thiazoline stereocenters in the thiamyxins as *R*‐configured as well. We observed demethylation of N‐Me‐Val under acidic conditions, wherefore the emerging Val was compared to a Val standard to assign the stereochemistry of this amino acid (see Figure S7). The stereocenter of N‐Me‐Val was hereby assigned as *S*‐configured. Additional NMR signal sets were detected for both cyclic derivatives **1** and **2**, in particular located surrounding the N‐Me‐Val signal sets. The ratio to the main signal set was constant (ca. 1 : 5) in both derivatives and in different isolated batches, indicating two conformational isomers even though NMR experiments at different temperatures and solvents did not yield a significant change of the conformer ratio (see Figure S5 and S6). The Ile stereocenter was found to racemize during hydrolysis, which could not be prevented by optimizing the conditions. Similar effects were observed previously for stereocenters adjacent to thiazoline moieties in the bottromycin biosynthesis and the synthesis of peptide thiazolines. We believe that racemization is promoted by the neighboring Me‐thiazoline in the thiamyxins.^[14]^ We therefore take into account in silico analysis of the biosynthetic domains for assignment of the Ile stereocenter. The presence of an epimerization domain in the Ile‐incorporating module M4 and the C‐domain of the following module M5, which is predicted to be a DLC domain, led to the conclusion that the Ile stereocenter is *R*‐configured (see below). After careful analysis of the 2D NMR data, the corresponding signals revealed that thiamyxins **1** and **2** are diastereomers. Configurational assignment was achieved based on observed shift and coupling constant differences of isoleucine vs. *allo*‐isoleucin (Figure S3).^[15]^ Based on this analysis, thiamyxin A (**1**) and thiamyxin B (**2**) are epimers in the 2‐Ile stereocenter. According to the high prevalence of l‐Ile in natural products, we assume thiamyxin A (**1**) to incorporate l‐isoleucine and thiamyxin B (**2**) d‐*allo*‐isoleucine, respectively. The open chain derivative **3** turned out to be a mixture of both isomers (ca. S : R 1 : 2 according to NMR) with exactly matching retention time on HPLC. The same holds true for thiamyxin D (**4**).

**Figure 3:**
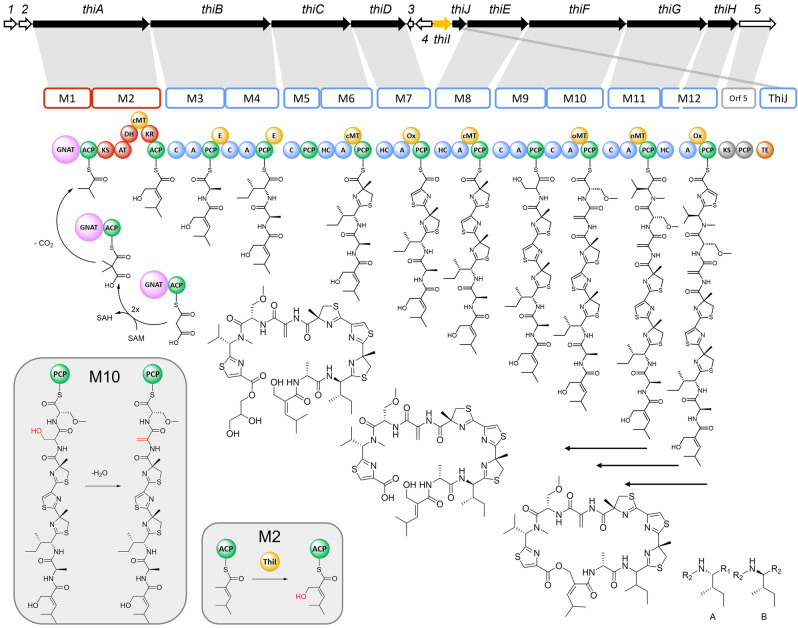
Proposed biosynthetic pathway for the thiamyxins (Map not drawn to scale). Core PKS modules are marked in red and core NRPS modules in blue, epimerization, methylation and oxidation domains are marked yellow and ACP and PCP domains in green. The thioesterase is shown in orange and the GNAT domain in pink. Modules proposed to be non‐functional are marked in grey. The genes involved in the thiamyxin biosynthesis are marked in black and named *thiA‐J*. The remaining genes with unknown or unassigned function are shown blank and named ORF1‐5. The hydroxylation by ThiI is shown in in the right box and the water elimination of Serine to form Dehydroalanine (module 10) in the left box. Gene cluster color code: NRPS genes (blue), PKS genes (red). SAM=S‐adenosyl methionine; SAH=S‐adenosyl homocysteine.

AntiSMASH analysis of the MCy9487 genome enabled identification of a PKS‐NRPS hybrid gene cluster capable of thiamyxin biosynthesis (Figure 3). It consists of two PKS and nine NRPS modules, which are encoded on nine genes (*thiA*‐*thiH*), as well as a cytochrome P450 dependent enzyme and a thioesterase domain encoded on *thiI* and *thiJ*, respectively. During bioinformatic analysis of the cluster we also found a fragmented but similar biosynthetic gene cluster in *Corallococcus terminator*, previously identified by Livingstone et al.^[16]^ Modules 3–12 largely follow textbook NRPS biosynthesis logic.^[17]^ The initiation of the biosynthesis, as proposed for modules 1 and 2 is unusual for PKS‐NRPS systems but has been described in similar fashion previously for loading of isovaleryl‐ and isobutyryl‐CoA by the GCN5‐related N‐acetyltransferase‐like decarboxylase (GNAT) domain. A detailed description of the gene cluster organization, including all genes of the BGC and their closest homologues, can be found in the Supporting Information. As all attempts to genetically manipulate strain MCy9487 failed and we could not identify a genetically manipulable alternative producer, we propose the following biosynthesis model based on in silico analysis of the BGC supported by feeding experiments with isotope labelled precursors:

The polyketide biosynthesis in thiamyxin is initiated by a GNAT domain. These domains have been shown to initiate polyketide biosynthesis by starter unit selection and decarboxylation, for example in the biosynthesis of the cytostatic polyketide gephyronic acid, as well as myxovirescin, rhizoxin, curacin and pederin.^[18]^ The formal starter unit of the thiamyxin biosynthesis is isobutyryl‐CoA, which is also the starter of the gephyronic acid biosynthesis. Isobutyryl‐CoA can be derived either from valine or dimethylmalonyl‐CoA. In the case of gephyronic acid dimethylmalonyl‐ACP is decarboxylated to generate the isobutyryl starter unit.^[18a]^ Analysis of the conserved residues of the thiamyxin GNAT domain confirmed the presence of arginine and threonine required for the decarboxylation. Analysis of the feeding experiment employing l‐methionine‐(methyl‐^13^C) indicated up to seven methyl incorporations into thiamyxin C, whereas feeding with l‐valine‐d_8_ resulted in only one incorporation which is explained by the N‐methylvaline incorporated by module 11. Thus, no second valine seems to be incorporated as would be expected if isovaleryl‐CoA formed from valine was the starter moiety. When feeding methionine‐(methyl‐^13^C), five of the observed incorporations can be accounted for by methyltransferases in modules 2, 6, 8, 10 and 11. To account for the remaining two methyl incorporations, we propose, that malonyl‐CoA is bis‐methylated to form dimethylmalonyl‐CoA. A conserved domain search of the *thiA* gene, revealed a dimerization domain directly upstream of the GNAT domain. This dimerization domain is commonly found in methyl transferases.^[19]^ We propose that the dimerization domain in combination with one of the methyltransferase domains present in the cluster is responsible for bis‐methylating malonyl‐CoA after it is loaded to the ACP in module 1 and thus this is the mechanism that forms the isobutyryl starter moiety.

Dimethylmalonyl‐ACP is then decarboxylated by the GNAT and transferred to module 2.^[18a]^ Module 2 extends the starter unit by one malonyl unit which we propose is subsequently methylated at the α‐carbon by the cMT domain located in the same module, analogous to the gephyronic acid biosynthesis.^[18c]^ This methyl group is then hydroxylated by the CyP450 encoded on *thiI*. This hydroxylation is required to take place on the assembly line as the resulting hydroxy group is necessary for final cyclization by the type I thioesterase located on *thiJ*. Modules 3 and 4 contain epimerization domains, which indicates that these incorporate d‐Ala and d‐Ile. Module 5 consists only of a C and a PCP domain and does not incorporate any building block, although the C domain seems to be functional based on analysis of its active site residues (see Supporting Information). The C domain is annotated as a DlC domain, which are responsible for forming amide bonds with d‐amino acids. As the previously incorporated amino acid is d‐Ile, we propose that this C‐domain has a transfer function that assists in loading of the intermediate onto the PCP‐domain of the next module 6. In module 6, a heterocyclization domain (HC) catalyzes the first cysteine incorporation followed by cyclization and methylation to form Me‐thiazoline as previously described in the bacillamide E biosynthesis.^[20]^ The methyl function is introduced by another cMT domain found in the same module. The following module 7 introduces a thiazole and contains an oxidation domain that oxidizes the formed thiazoline introducing the double bond between the four and five position. Module 8 incorporates another Me‐thiazoline, comparable to module 6. Modules 9 and 10 each introduce serine into the nascent molecule as confirmed by feeding experiments (Supporting Information). The serine incorporated in module 9, is dehydrated to Dh‐Ala in the final product. A new clade of C domains, that are associated with dehydration reactions in NRPS assembly lines have recently been reported.^[21]^ The condensation domain of module 10 was identified as belonging to this new clade of C domains by phylogenetic analysis. (See Supporting Information) We therefore propose that the C domain of module 10 facilitates the dehydration of the serine incorporated by module 9 to form dehydroalanine and also incorporates another serine. The second serine is subsequently O‐methylated by an oMT domain. Module 11 introduces a valine which is N‐methylated by an nMT domain in the same module. Module 12 is split on two genes: *thiG* and *thiH*. This module introduces a thiazole, analogous to module 7. The TE domain encoded on *thiJ*, finally releases the molecule from the assembly line by cyclic condensation with the hydroxyl function installed by the CYP‐450 in module 2. The derivative thiamyxin D likely is a shunt product, created during the release process. We propose that the type I TE domain promiscuously accepts glycerol as substrate next to the HMMP‐hydroxy function required for the intramolecular cyclization reaction. Similar glycerol ester formation has been previously observed in tubulysin biosynthesis.^[23]^ Thiamyxin C might also be created in a similar fashion by promiscuity for H_2_O but could also be an artefact from the purification process. Downstream of the TE Domain a KS and PCP di‐domain are encoded on orf5. Analysis of their active site residues revealed them to be non‐functional, which is in line with our biosynthesis hypothesis, as there are no non‐assigned biosynthetic reactions remaining to form the mature thiamyxins.

After determination of their chemical structure, the thiamyxins were evaluated against a broad panel of bacterial, fungal and viral pathogens, alongside with their antiproliferative effects on human cell lines (Table 1, Figures S17–S22, Table S3). A detailed description of the underlying assays and test organisms against which the thiamyxins were found inactive or only presented weak biological activities can be found in the Supporting Information. Against the antimicrobial test panel, the thiamyxins only presented weak activity at a minimal inhibitory concentration (MIC) of 32–64 μg mL^−1^ against two fungal test organisms (*Candida albicans* and *Mucor hiemalis*) and two Gram‐positive pathogens (*Bacillus subtilis* and *Micrococcus luteus*) (Supporting Information‐Table S3). Thiamyxin D did not show effects in the tested concentrations.

**Table 1:** Antiviral and antiproliferative activities of the thiamyxins. Half maximal inhibitory concentrations against three RNA viruses (IC_50_) values determined simultaneously to half maximal cytotoxic concentrations (CC_50_) in infected cells.

Test organism		IC_50_ and corresponding CC_50_ [μM]			
		Thiamyxin A	Thiamyxin B	Thiamyxin C	Thiamyxin D
**hCov‐229E‐luc** ^[a]^	IC_50_	2.47	2.39	>10	>10
Huh‐7.5 Fluc infected	CC_50_	>10	>10	>10	>10
**DENV‐R2A** ^[b]^	IC_50_	nd	0.56	14.56	nd
Huh‐7 infected with DENV‐R2A	CC_50_	nd	2.25	>50	nd
**ZIKV‐H/PF/2013** ^[c]^	IC_50_	nd	1.07	>15	nd
Huh‐7 infected with ZIKV‐H/PF/2013	CC_50_	nd	2.25	>50	nd

[a] positive control remdesivir IC_50_=5.6 nM.^[25]^ [b]  positive control ribavirin IC_50_=2.3 μM. [c] positive control ribavirin IC_50_=2.5 μM nd=not determined.

Thiamyxin B and C which are produced in higher amounts (0.6 and 1.4 mg L^−1^ isolated yield) and represent one cyclized and one open chain analogue were evaluated for activity in cell‐based assays against the human pathogenic corona virus hCoV‐229E and two representatives of the flavivirus genus, Dengue virus and Zika virus, for which we used the easy to measure reporter viruses hCoV‐229E‐luc, DENV‐R2A and ZIKV‐H/PF/2019, respectively.^[24]^ These assays allow simultaneous determination of antiviral activity and cytotoxic effects on the chosen cell line (results see Table 1). Due to their comparably low production rates of <0.2 mg L^−1^ isolated yield, thiamyxin A and D were only evaluated against a selected panel of test organisms.

The cyclized congeners thiamyxin A and B were found to effectively inhibit hCoV‐229E‐luc with a half maximal inhibitory concentration (IC_50_) in the low micromolar range. This activity was highly decreased for the open chain analogues thiamyxin C and D, which showed IC_50_ values above our tested concentrations of around 20 μM.

This trend is also observed for DENV‐R2A and ZIKV‐H/PF/2019, where thiamyxin B was found to inhibit the two viruses with an IC_50_ of 560 nM and 1.07 μM, respectively. In constrast, thiamyxin C inhibited DENV‐R2A with an IC_50_ value of 14.56 μM and was completely inactive against ZIKV‐H/PF/2019 (Table 1).

**Figure 4:**
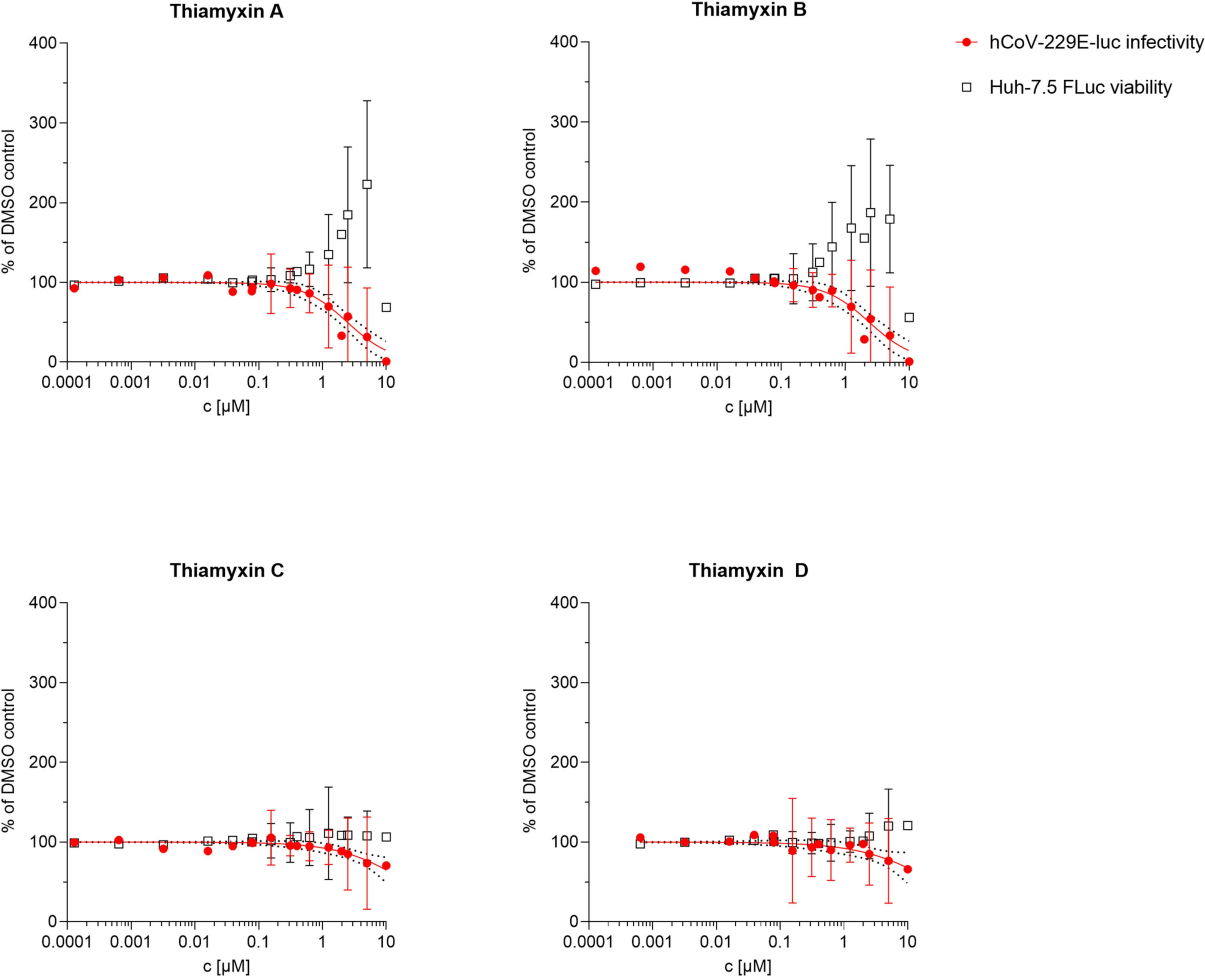
Antiviral activity of thiamyxin A–D against HCoV‐229E‐luc (red) when simultaneously determining their effect on Huh‐7.5 Fluc cells (black). Renilla luciferase serves as reporter for viral load, firefly luciferase for cell viability. Measurements performed in technical duplicates of four independent biological experiments. Non‐linear regression curves (red) are given with 95 % confidence interval (black dots). Increase in cell viability over 100 % caused by reduction in viral load.

As depicted in Figure 4, we observed a potential application window for the two cyclized thiamyxins A and B in the HCov‐229E assay, where we can see a clear separation of antiviral and cytotoxic activity with a more than fivefold difference between IC_50_ and CC_50_. This indicates a distinct mode of action for the antiviral activity compared to the cytotoxic effects. Among the three viral pathogens, DENV‐R2A shows best inhibition with a fourfold lower IC_50_ value compared to HCov‐229E‐luc for thiamyxin B. In this assay, however, the determined application window is smaller compared to HCoV‐229E with only a fourfold difference between IC_50_ and CC_50._ The extend of cytotoxic effects of the thiamyxins therefore seem to be cell line dependent.

## Conclusion

In this study, we present the thiamyxins, a family of cyclic thiazole‐ and thiazoline rich non‐ribosomal peptides, which we isolated from the myxobacterial strain MCy9487 discovered at the Saarland University campus. A combination of NMR analyses and detailed stereochemical configurational studies supported by bioinformatics analysis of the BGC responsible for their formation led to their complete stereochemical assignment. We developed a concise biosynthesis model for the thiamyxins, including the formation of their unusual isobutyryl starter unit, which presumably is formed in a similar fashion to gephyronic acid. Based on the underlying biosynthetic logic, thiamyxin B seems to be the main product of the thiamyxin BGC assembly line, which is also reflected in its higher production rate compared to thiamyxin A. The two open chain analogues thiamyxin C and D likely are shunt products, generated due to promiscuity of the release enzyme for gylcerol and water in competition with the preferred intramolecular HMMP‐hydroxy function. Thiamyxin B also shows the most potent biological activity among the four characterized derivatives. It shows significant activity against RNA viruses but also shows cytotoxic effects, which however seem to be cell‐line‐dependent and can be well separated from the observed antiviral activity. Due to the comparably low production rates of the thiamyxins, biotechnological production optimization, biosynthetic engineering approaches or development of a total synthesis route would be of great interest allowing their in‐depth evaluation against a broader panel of human pathogenic viruses and studying their cytotoxicity profile on non‐infected human cells. Those approaches would furthermore allow access to further derivatives, giving insight into structure‐activity relationships of the thiamyxins and enabling evaluation of the pharmacokinetic properties of this interesting NP class. In this study, we could already observe significantly lower antiviral activities for the open‐chain analogue thiamyxin C as compared to the two cyclic thiamyins A and B, but the data still suggest some residual antiviral activity at higher micromolar concentrations. This finding is particularly interesting, as most cyclic NRPs almost completely lose affinity to their target because their 3D structure is altered by opening the macrocycle. The resembling biological activities of thiamyxin A and B are unexpected, as their proposed incorporation of d‐ versus l‐isoleucine results in a distinct conformation of the two macrocycles (see Supporting Information). Determining the thiamyxins’ antiviral target ‐ besides developing a total synthesis route for them ‐ could provide deeper insight into the thiamyxin pharmacophore and help to understand these observations.

In summary, the thiamyxins are a structurally unique class of NPs showing a potential application window as broad spectrum antivirals targeting human pathogenic RNA viruses and their desription herein paves the way for their further investigation.

## Conflict of interest

The authors declare no conflict of interest.

1

## Supporting information

As a service to our authors and readers, this journal provides supporting information supplied by the authors. Such materials are peer reviewed and may be re‐organized for online delivery, but are not copy‐edited or typeset. Technical support issues arising from supporting information (other than missing files) should be addressed to the authors.

Supporting Information

## Data Availability

The data that support the findings of this study are available in the Supporting Information of this article.
