# Supplementary material for: Thiamyxins: Structure and Biosynthesis of Myxobacterial RNA‐Virus Inhibitors
Source: Angew Chem Int Ed Engl. 2022 Nov 28;61(52):e202212946. doi: 10.1002/anie.202212946 (PMC10100342; doi:10.1002/anie.202212946)
Supplement: Supplementary file 1 — Supporting Information [file ANIE-61-0-s001.pdf]

Angewandte Chemie
Eine Zeitschrift der Gesellschaft Deutscher Chemiker

Supporting Information

Thiamyxins: Structure and Biosynthesis of Myxobacterial RNA-Virus Inhibitors

*P. A. Haack, K. Harmrolfs, C. D. Bader, R. Garcia, A. P. Gunesch, S. Haid, A. Popoff, A. Voltz, H. Kim, R. Bartenschlager, T. Pietschmann, R. Müller**

Contents

1. General Experimental Procedures and Materials 4
1.1 Isolation and cultivation of MCy9487 4
1.2 Isolation of the thiamyxins 4
1.3 LC-MS systems 4
1.3.1 LC-MS system 1a – standard analytical measurements 5
1.3.2 LC-MS system 1b – Marfey's Method 5
1.3.3 LC-MS system 1c – adapted Marfey's Method for improved separation of isoleucins .. 5
1.3.4 LC-MS system 2 – semi-preparative purification 5
1.4 NMR measurements 6
1.5 Feeding experiments 6
1.6 Biological assays 7
1.6.1 Screening with hCoV-229E-luc reporter virus 7
1.6.2 Screening with dengue and Zika virus 7
2. Supporting Data 8
2.1 Bioactivity data of MCy9487 crude extract 8
2.2 NMR-based structure elucidation 8
2.3 Stereochemistry assignment 14
2.3.1 N-methyl-valine assignment 14
2.3.2 Isoleucine assignment 15
2.3.3 Thiazoline assignment 18
2.3.4 Stability of thiamyxin A 18
2.4 Gene cluster organization and proposed biosynthesis 19
2.4.1 Active site residues of module 5 (*thiC*) 21
2.4.2 Active site residues of orf 5 21
2.4.3 Dimerization domain in thiA 22
2.4.4 Alignment of GNAT Domains 23
2.4.5 Phylogenetic analysis of C domains 24
2.5 Feeding Experiments 24

2.6 Antimicrobial activities 26

2.7 Antiviral activities 26

2.8 NMR data 30

2.8.1 Thiamyxin B 30

2.8.2 Thiamyxin A 38

2.8.3 Thiamyxin C 45

2.8.4 Thiamyxin D 53

3 References 60

1. General Experimental Procedures and Materials

1.1 Isolation and cultivation of MCy9487

The myxobacterial strain MCy9487 was isolated at the hillside of Saarland University Campus, Saarbrücken, Germany in January 2011 from a soil sample with leaf litters and other decaying plant materials. The strain was recognized on a standard mineral salts agar isolation medium for transparent swarming and whitish fruiting bodies appearing as mounds- and horn-like shapes. Phylogenetic analysis based on 16S rRNA gene sequence revealed the strain's distinct position within the *Myxococcus-Pyxidicoccus-Corallococcus* clade which shows 98.62 – 98.68% similarity with *Corallococcus coralloides* strain M2^T (GenBank accession: NR_042329) and *C. exiguus* strain Cc e167^T (GenBank accession: NR_042330), respectively. The detailed and valid strain description will be published in a separate taxonomic manuscript.

The fermentation was performed in 30 L VY/2S medium (g/L, w/v: 0.5% commercial fresh Baker's yeast, 0.01% $CaCl_2$. $2H_2O$, 0.2% HEPES, 0.5% soluble starch (Roth), pH 7.2, adjusted with KOH before autoclaving) supplemented with 2% (v/v) amberlite resin XAD-16 (Sigma). The cultivation was maintained under rotary shaking condition (160 r.p.m., 30°C, 10d). At the end of cultivation, the cells and resins were harvested together by centrifugation (8,000 r.p.m. 30mins, 4°C).

1.2 Isolation of the thiamyxins

The cell pellet and resin were extracted three times with methanol and dried using a rotary evaporator. The extract was then resolved in Methanol : H_20 (60:40) and extracted with hexane. The methanol was then removed by rotary evaporation and the remaining aqueous layer was extracted with chloroform. All four compounds were found in the chloroform partition, which was then dried, resolved in methanol and the compounds were purified using LC-MS system 2.

1.3 LC-MS systems

All analytical LC-MS measurements were performed on a Dionex Ultimate 3000 RSLC system using a BEH C18, 100 x 2.1 mm, 1.7 µm dp column (Waters, Germany), coupled to a maXis 4G hr-ToF mass spectrometer (Bruker Daltonics, Germany) using the Apollo ESI source. UV spectra were recorded by a DAD in the range from 200 to 600 nm. The LC flow was split to 75 µL/min before entering the mass spectrometer. The LC-MS data was analyzed using Bruker DataAnalysis version 4.2.

1.3.1 LC-MS system 1a – standard analytical measurements

Separation of 1 µl sample was achieved by a linear gradient from (A) H_2O + 0.1% FA to (B) ACN + 0.1% FA at a flow rate of 600 µL/min and 45 °C. The gradient was initiated by a 0.5 min isocratic step at 5% B, followed by an increase to 95% B in 18 min to end up with a 2 min step at 95% B before reequilibration under the initial conditions. Mass spectra were acquired in centroid mode ranging from 150 – 2500 *m/z* at a 2 Hz scan rate.

1.3.2 LC-MS system 1b – Marfey's Method

Separation of 1 µl sample was achieved by a gradient from (A) H_2O + 0.1% FA to (B) ACN + 0.1% FA at a flow rate of 600 µL/min and 45 °C. The gradient was as follows: Ramp in 1 min from 5% B to 10% B, in 14 min to 35% B, in 7 min to 55% B and in 3 min to 80% B. This is followed by a 1 min step at 80% B before reequilibration with the initial conditions. Mass spectra were acquired in centroid mode ranging from 250 – 3000 *m/z* at a 2 Hz scan rate.

1.3.3 LC-MS system 1c – adapted Marfey's Method for improved separation of isoleucins

Separation of 1 µl sample was achieved by a gradient from (A) H_2O + 0.1% FA to (B) ACN + 0.1% FA at a flow rate of 600 µL/min and 45 °C. The gradient was as follows: Ramp in 1 min from 5% B to 27% B, in 37 min to 45% B and in 1 min to 80% B. This is followed by a 1 min step at 80% B before reequilibration with the initial conditions. Mass spectra were acquired in centroid mode ranging from 250 – 3000 *m/z* at a 2 Hz scan rate.

1.3.4 LC-MS system 2 – semi-preparative purification

The final purification was performed on a Dionex Ultimate 3000 SDLC low pressure gradient system using a Luna, 5u, C18(2), 100A, 250 x 100 mm column (Phenomenex). Separation of 80 µl sample was achieved by a gradient from (A) H_2O + 0.1 % FA to (B) ACN + 0.1 % FA at a flow rate of 5 mL/min and 45 °C. The gradient was as follows: A two min isocratic step at 40 %B, followed by a ramp to 80 %B in 15 min, a two min plateau, return to initial conditions in 1 min and re-equilibration for two min. UV spectra were recorded by a DAD in the range from 200 to 600 nm. The LC flow was split to 0.525 mL/min before entering the Thermo Fisher Scientific ISQTM EM single quadrupole mass spectrometer. Mass spectra were acquired by selected ion monitoring (SIM) at *m/z* 479.68 $[M+2H]^{2+}$, 488.68 $[M+2H]^{2+}$, 525.70 $[M+2H]^{2+}$ and fraction collection times were set accordingly.

1.4 NMR measurements

NMR spectra were recorded on a 500 MHz *Avance III* (UltraShield) spectrometer or on a 700 MHz *Avance III* (Ascend) spectrometer by Bruker BioSpin MRI GmbH, each equipped with a Helium cooled CryoProbe (TCI), at 298 K, if not stated differently. Chemical shift values of ^{1}H- and ^{13}C-NMR spectra are reported in ppm relative to the residual solvent signal (DMSO-d_6, δ_H 2.50 ppm and δ_C 39.5 ppm) given as an internal standard. ^{13}C-signals were assigned via 2D-CH and CCH or CNH correlations (HSQC and HMBC), using Bruker standard pulse programs. HSQC experiments were optimized for $^{1}J_{C\text{-}H}$ = 145 Hz and HMBC experiments were optimized for $^{2,3}J_{C\text{-}H}$ = 6 Hz. Multiplicities are described using the following abbreviations: s = singlet, d = doublet, t = triplet, q = quartet, m = multiplet, b = broad; coupling constants are reported in *Hz*.

1.5 Feeding experiments

4 x 50 mL cultures of Mx152 in M8-CyH medium (1.5 g/L casein peptone, 1.5 g/L yeast extract, 1.0 g/L glucose monohydrate, 1.0 g/L soy flour (degreased), 4.0 g/L starch, 1.0 g/L $CaCl_2$ dihydrate, 0.5 g/L $MgSO_4$ heptahydrate, 0.004 g/L Na-Fe-EDTA, 11.8 g/L HEPES pH 7.2, adjusted with NaOH before autoclaving) were inoculated with 5mL of preculture. Each culture was supplemented with 50 µL of a 0.1 M stock solution of one of the labeled aminoacids (L-Valine-d_8, L-Serine-2,3,3-d_3 and L-Methionine-(methyl-^{13}C)). One culture was supplemented with sterile H_2O as a control. After 24 h, 48 h and 72 h, an additional 50 µL of the stock solution was added. After incubation for 8 days at 30 °C and 180 rpm the cultures were harvested by centrifugation. The pellets were then extracted with 50 mL of methanol, dried using a rotary evaporator, redissolved in 1 mL of methanol, and analyzed using LC-MS system 1a.

1.6 Biological assays

1.6.1 Screening with hCoV-229E-luc reporter virus

Firefly luciferase-expressing Huh-7.5/Fluc cells were infected with a *Renilla* luciferase reporter virus of the alphacoronavirus HCoV-229E-luc one day after seeding (2x10^4 cells/well in a 96 well plate) in the presence of indicated thiamyxin concentrations or DMSO.[1] After 48h post inoculation and incubation of the cells at 33 °C and 5% CO_2, the virus inoculum was removed, cells were washed with PBS and lysed in 50 µl PBS/0,5% Triton X-100. Lysis of cells was further enhanced by freezing of the plates at -20 °C. To determine the cell viability, firefly luciferase activity was measured in 20 µl of the lysate, whereas residual virus replication/infection efficiency was measured in 20 µL of lysate via the *Renilla* luciferase activity (Berthold Centro plate luminometer version 2.02). Mean values were normalised to DMSO treated infected samples and standard deviations of technical duplicates of four independent biological experiments are depicted. Non-linear regression curve and confidence interval (confidence level 95%) were both calculated with GraphPad Prism 9 and half-maximal inhibitory (IC_{50}) and cytotoxic (CC_{50}) concentrations were interpolated from the curves. As previously described, remdesivir served as positive control for the assay giving an IC_{50} value of 5.6 nM.[2]

1.6.2 Screening with dengue and Zika virus

Huh-7 cells were seeded at 1.5 x 10^4 cells per well in 96 well plates. On the next day, cells were treated with 10 different concentrations of 3 fold serial diluted compounds ranging from 2.54 nM to 50 µM. Cells were inoculated with DENV2-R2A or ZIKV-R2A reporter virus using a multiplicity of infection (MOI) of 0.1. At 48 h post infection, cells were lysed and *Renilla* luciferase activity contained in cell lysates was quantified. To determine cytotoxicity of the compounds, cell viability was measured in parallel to antiviral activity test using the CellTiter Glo® Luminescent Cell Viability Assay (Promega) according to manufacturer’s instruction. Values were normalized using non-treated solvent control (0.5% DMSO) and IC_{50} and CC_{50} values were calculated using non-linear regression dose response analysis of the Prism 7 software package (GraphPad Software). Ribavirin was used as positive control giving an IC_{50} value of 2.3 µM for DNV2-R2A and 2.5 µM for ZIKV-R2A, without showing effects on Huh-7 cells (>90% cell viability) up to 100 µM concentration.

2. Supporting Data

2.1 Bioactivity data of MCy9487 crude extract

Table 1 Activity of strain MCy9487 extracted after 10-d cultivation in CYH medium. Activity determined by serial dilution as previously described[3]. 0- No activity, A- 20 µl extract (lowest tested dilution) causing activity, H- 0.16 µl extract (highest tested dilution) causing activity .

Test organism	*Activity score*
Escherichia coli TolC	E
Escherichia coli DSM 1116	0
Pseudomonas aeruginosa PA14	0
Micrococcus luteus DSM 1790	E
Mycobacterium smegmatis mc2-155	A
Chromobacterium violaceum DSM 30191	C
Staphylococcus aureus Newman	D
Bacillus subtilis DSM 10	D
Mucor hiemalis DSM 2656	D
Candida albicans DSM 1665	C
Wickerhamomyces anomalus DSM 6766	H

2.2 NMR-based structure elucidation

Thiamyxin B is assigned a molecular formula of $C_{43}H_{59}N_9O_8S_4$ on the basis of HR-ESI-MS data ($[M+H]^+_{meas}$ = 958.3449, $[M+H]^+_{calc}$ = 958.3442, Δ = 0.7 ppm). Interpretation of the 1D and 2D NMR spectra (Table 5-8, Figure 26-43) —which is described in detail in the following—,alongside with their characteristic MS^2 fragmentation pattern, reveals thiamyxin B to consist of the following peptide sequence: 2-(hydroxymethyl)-4-methylpent-3-enoic acid(HMMP)—alanine(Ala)—isoleucine(Ile)—Methyl-thiazoline(Me-thiazoline)—Thiazole—Me-Thiazoline—Dehydro-alanine(Dh-Ala)—Methylserine(Me-Ser)—N-Methylvaline(N-Me-Val)—Thiazole. In the ^{1}H and HSQC spectra of thiamyxin B we detect four α-protons with characteristic chemical shifts of δ_H 4.61 (Ala), 4.78 (Ile), 4.89 (Me-Ser) and 5.31 ppm (N-Me-Val). We find two signals corresponding to the terminal protons of an exo double bond at δ_H = 6.37 and 5.81 ppm (Dh-Ala), as well as three methine groups at δ_H 8.30, 8.57 and 5.83 ppm, whereof the deshielded shift of the two protons at δ_H 8.30 and 8.57 indicate their incorporation in the thiazole units and the more shielded shift of the methine group at δ_H 5.83 ppm indicates it as the HMMP aliphatic double bond proton. There are three additional shielded methine groups detectable at δ_H 1.91 (Ile), 2.83 (HMMP) and 2.36 ppm (N-Me-Val), which are—based on their characteristic chemical shifts—branching positions in the aliphatic sidechains of the respective amino acids. Furthermore, we find five methylene groups at δ_H 1.36/1.21 (Ile), 3.74 and 3.38/3.81 (Me-Thiazoline), 3.59 (Me-Ser) and 4.89 ppm (HMMP). The shielded shifts of the carbon atoms corresponding to the two proton signals at δ_H 3.59 (Me-Ser) and 4.89 ppm (HMMP) in the HSQC spectra at δ_C 71.0 and 67.0 ppm indicate their allocation next to an oxygen atom, whereas the deshielded shift of the methylene at δ_H 1.36/1.21 ppm,

besides its characteristic splitting pattern allows its assignment as part of the aliphatic chain in Ile next to a stereo center. We detect eleven methyl groups at δ_H 0.96 (HMMP), 0.96 (HMMP), 1.36 (Ala), 0.93 (Ile), 0.87 (Ile), 1.59 (Me-Thiazoline), 3.19 (Me-Ser), 0.38 (N-Me-Val), 0.71 (N-Me-Val), 2.90 (N-Me-Val) and 1.65 (Me-Thiazoline), whereof the shielded shift of the carbon at δ_C 58.4 ppm corresponding to δ_H 3.19 ppm (Me-Ser) points towards its allocation next to an oxygen atom and the slightly shielded shift of the carbon at δ_C 30.2 ppm corresponding to δ_H 2.90 (N-Me-Val) towards its allocation next to an nitrogen atom. Lastly, we find four broad signals in the proton spectrum at 7.51, 8.30, 9.36 and 8.97 ppm without corresponding signals in the HSQC, which we assign as peptide bond forming amino groups belonging to Ala, Ile, Dh-Ala and Me-Ser, respectively. Ala was identified as follows: The α-proton at δ_H 4.61 ppm shows COSY correlations to the methyl group at δ_H 4.61 ppm, as well as to the NH proton at δ_H 7.51 ppm. Both the α-proton and the methyl group reveal HMBC correlations to a quaternary carbon at δ_C 171.6 ppm, which was assigned as the amide function connecting Ala and Ile based on its HMBC correlations to the Ile α-proton. The Ala α-proton furthermore shows HMBC correlations to a quaternary carbon at δ_C 165.8 ppm assigned to HMMP, indicating their peptide bond connection between the respective functions. Ile was identified as follows: The Ile α-proton at δ_H 4.78 ppm shows COSY correlations to the methine group at δ_H 1.91 ppm, as well as to the NH proton at δ_H 8.30 ppm. The methine group in turn reveals COSY correlations to the methyl group at δ_H 0.93 and the methylene 1.36/1.21 ppm. The methylene group reveals COSY correlations to another methyl group at δ_H 0.87 ppm. Both the α-proton and the methine group reveal HMBC correlations to a quaternary carbon at δ_C 173.1 ppm, which was assigned as the thioamid function connecting Ile and Me-Thiazoline based on its HMBC correlations with the Me-Thiazoline methylene group. The first Me-Thiazoline was identified as follows: The Me-Thiazoline methylene group at δ_H 3.74 ppm reveals HMBC correlations to the methyl group at δ_H 1.65 ppm, as well as two quaternary carbons at δ_C 176.5 and 83.3 ppm. Based on the characteristic chemical shift of the quaternary carbon at δ_C 83.3 ppm and its correlations to both the methyl and methylene group, it was assigned as Me-Thiazoline α-carbon. The quaternary carbon at δ_C 176.5 ppm shows additional correlations with the Thiazole methine group at 8.30 ppm, indicating it as thiamid function connecting the first Me-Thiazoline with Thiazole. The first Thiazole was identified as follows: The first Thiazole methine group at δ_H 8.30 ppm shows HMBC correlations to two quaternary carbons at δ_C 147.5 and 163.8 ppm. Based on its shielded chemical shift and correlations with the second Me-Thiazoline methylene function at δ_H 3.38/3.81 ppm, the quaternary carbon at δ_C 163.8 ppm forms the thiamid bond between Thiazole and the second Me-Thiazoline. The second Me-Thiazoline was identified as follows: The second Me-Thiazoline methylene group at δ_H 3.38/3.81 ppm shows HMBC correlations to the methyl group at δ_H 1.59 ppm, as well as two quaternary carbons at δ_C 172.9 and 84.9 ppm. Based on the characteristic chemical shift of the quaternary carbon at δ_C 84.9 ppm and its correlations to both the methyl and methylene group, it was

assigned as Me-Thiazoline α-carbon. The quaternary carbon at δ_C 172.9 ppm shows additional correlations with the Dh-Ala NH proton at δ_H 9.36 ppm, indicating peptide bond formation here. Dh-Ala was identified as follows: The exo-double bond protons at δ_H 6.37 and 5.81 ppm show HMBC correlations to two quaternary carbons at δ_C 132.7 and 163.1 ppm. The characteristic chemical shift of the quaternary carbon at δ_C 132.7 identifies it as participating in the double bond, whereas the quaternary carbon at δ_C 163.1 ppm was identified as amid function connecting Dh-Ala with Me-Ser based on its characteristic chemical shift, as well as correlations to the Me-Ser α-proton. Me-Ser was identified as follows: The Me-Ser α-proton at δ_H 4.89 ppm shows COSY correlations to the methylene group at δ_H 3.59 ppm, as well as to the NH proton at δ_H 8.97 ppm. The methylene group reveals HMBC correlations to a methyl group at δ_H 3.19 ppm, which was assigned as methoxy function based on its characteristic chemical shifts and splitting pattern as a singlet. Both the α-proton and the methylene group reveal HMBC correlations to a quaternary carbon at δ_C 170.6 ppm, which was assigned as the amide function connecting Me-Ser and N-Me-Val based on its HMBC correlations to the N-Me-Val α-proton. N-Me-Val was identified as follows: The α-proton at δ_H 5.31 ppm shows COSY correlations to the methine group at δ_H 2.36 ppm. The methine group in turn reveals COSY correlations to two methyl groups at δ_H 0.38 and 0.71 ppm. For both the α-proton and the methine group the HMBC correlations to the quaternary carbon at δ_C 168.3 ppm do not exceed the limit of detection, but its characteristic chemical shift and correlations with the second Thiazole methine at 8.57 ppm allow its assignment as the amide function connecting N-Me-Val and the second Thiazole. Further proof for this assignment was given by the NMR spectra acquired for thiamyxin A (Table 6, Figure 31-34) where the respective correlations are detectable. The NMe group at δ_H 2.90 ppm, which was assigned based on its characteristic chemical shift, is located at the Val amino function based on its HMBC correlations to both the N-Me-Val α-proton and the Me-Ser amide function. The second Thiazole was identified as follows: The second Thiazole methine group at δ_H 8.57 ppm reveals HMBC correlations to two quaternary carbons at δ_C 145.2 and 160.5 ppm. Based on its shielded chemical shift and correlations with the HMMP methylene function at δ_H 4.89 ppm, the quaternary carbon at δ_C 160.5 ppm forms the ester bond between Thiazole and HMMP. HMMP was identified as follows: The HMMP methylene function participating in the ester bond at δ_H 4.89 ppm shows HMBC correlations to a methine group a δ_H 5.83 ppm, as well as two quaternary carbons at δ_C 129.2 and 165.8 ppm. Based on their characteristic chemical shifts, the quaternary carbon at δ_C 129.2 ppm and the methine group at δ_H 5.83 ppm participate in a double bond. The methine group reveals COSY correlations to another more deshielded methine group at δ_H 2.83 ppm, which in turn shows correlations with two methyl groups at δ_H 0.96 ppm.

Their consistent peptide sequence and exact mass but different retention time on HPLC-MS indicate thiamyxin A ($[M+H]^+_{meas}$ = 958.3448, $[M+H]^+_{calc}$ = 958.3442, Δ = 0.6 ppm) and B to be diastereomers.

This finding is in-line with the identical correlations observed in the NMR data acquired for thiamyxin A and B, as well as their very similar chemical shifts, except in the Ala and Ile region (see Table 5 and 6). The detailed analysis of this effect is described in the main manuscript and depicted in Figure 4 in this supporting information. The characteristic chemical shift of the HMMP methylene at δ_H = 4.89 and δ_C = 67.0 ppm, alongside with its HMBC correlation to the thiazole carboxy function implies cyclisation between the C-terminal thiazole and HMMP primary alcohol for both thiamyxin A and B. In contrary, thiamyxin C (Table 7, Figure 35-39) shows a shielded chemical shift at this position of δ_H = 4.00 and δ_C = 63.1 ppm with no signal split, which—in accordance with its sum formula of $C_{43}H_{61}N_9O_9S_4$($[M+H]^+_{meas}$ = 976.3556, $[M+H]^+_{calc}$ = 976.3548, Δ = 0.8 ppm)—reveals it to be the hydrolyzed non-cyclized congener (Figure 1). The molecular mass of thiamyxin D is further increased by $C_3H_6O_2$ ($[M+H]^+_{meas}$ = 1050.3928, $[M+H]^+_{calc}$ = 1050.3916, Δ = 1.1 ppm) when compared to thiamyxin C. Chemical shifts of the HMMP methylene in thiamyxin D only shows a deviation of 0.1 ppm for δ_C and 0.0 ppm for δ_H when compared to the C derivative, affirming that thiamyxin D also belongs to the open chain derivatives. In comparison to thiamyxin A-C, HSQC spectra of thiamyxin D (Table 8, Figure 40-43) show two additional methylene groups at δ_H 4.16/4.28 and 3.41 ppm, as well as one additional methine group at δ_H 3.76 ppm. Their characteristic chemical shifts reveal them all as hydroxylated and COSY correlations the methylene at 4.16/4.28 with the methine function, as well as COSY correlations of the methine group with the second methylene group at 3.41 disseminates the additional part of the molecule to be glycerol. The methylene group at 4.16/4.28 ppm furthermore shows correlations to the N-Me-Val C-terminus, revealing the N-Me-Val quaternary carbon at 172.2 ppm to participate in the ester bond with the respective glycerol methylene.

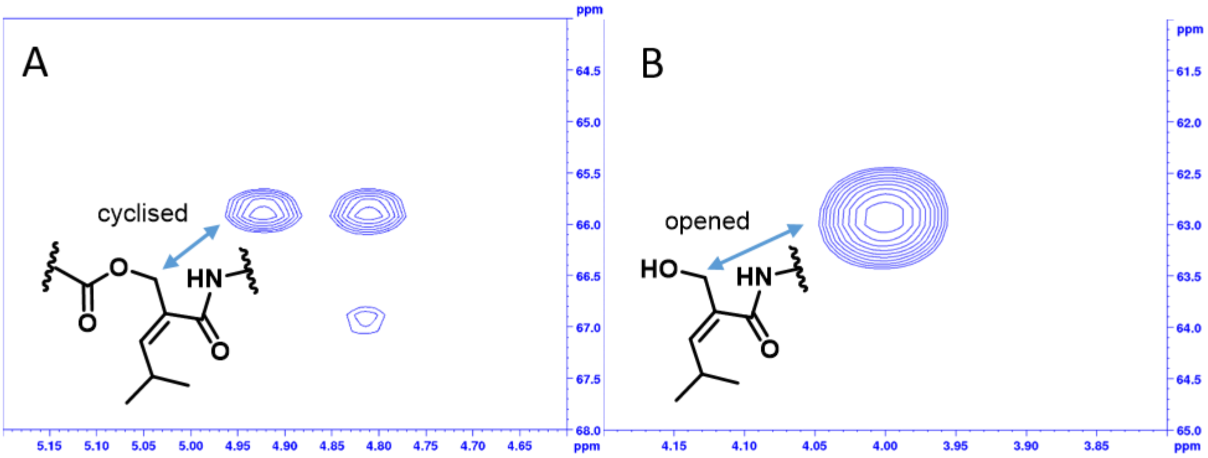

Figure 1 HSQC NMR signal of HMMP-CH_2 group of cyclized thiamyxin A (**A**) compared to open chain thiamyxin C (**B**) derivative.

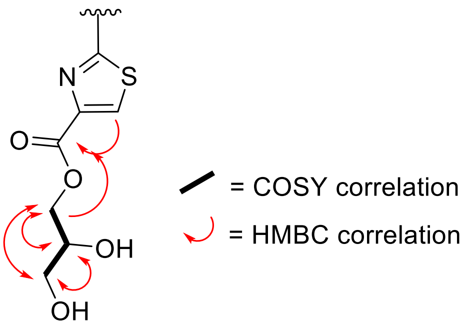

Figure 2 Glycerol attachment to thiazole unit in thiamyxin D derivative.

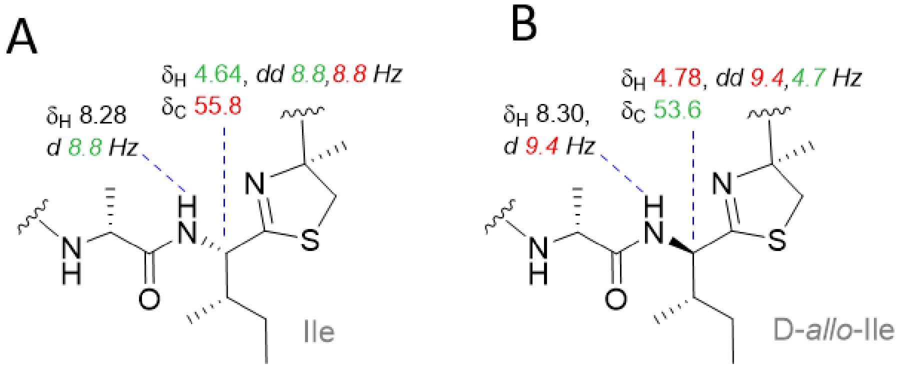

Figure 3 Analysis of observed shift and coupling constant differences of isoleucine vs. allo-isoleucin in thiamyxin A (**A**) and B (**B**). Larger values in red, smaller values in green are exactly matching the pattern shown by Anderson et al., in 2017.[4]

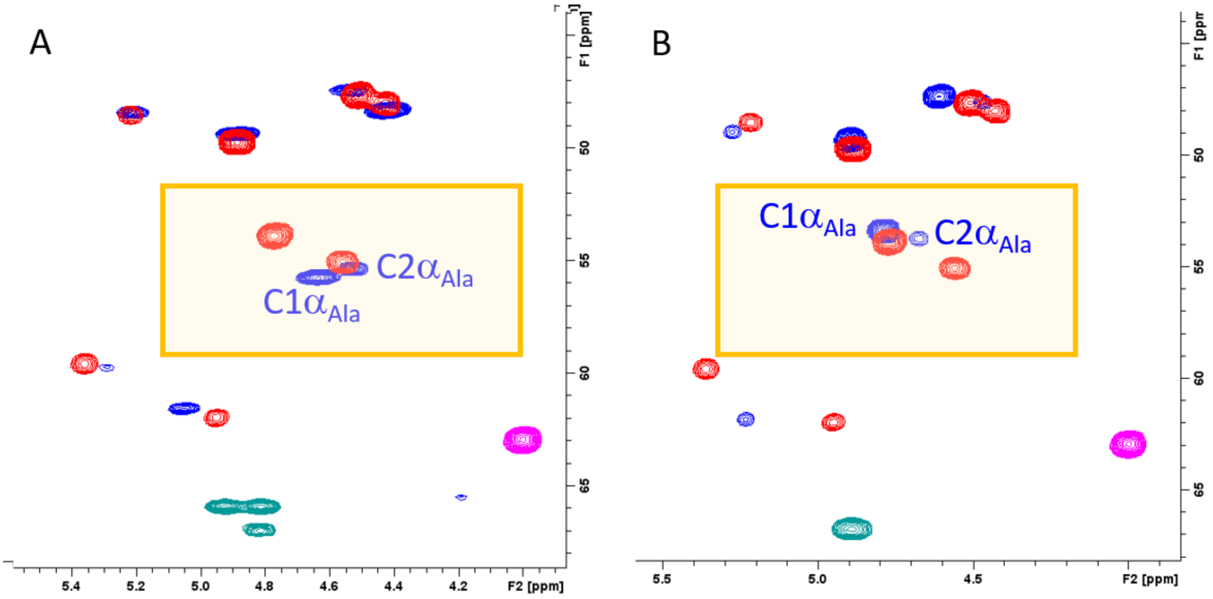

Figure 4 HSQC NMR section of thiamyxin A (**A**, blue/green signal set) and thiamyxin B (**B**, blue/green signal set), each overlapped with open chain derivative thiamyxin C (red/pink signal set). The highlighted signal sets were assigned to alpha-CH of the Alanin residue (Ala). Detailed analysis revealed both diastereomers (Ile and *allo*-Ile carrying derivative) in the open chain derivatives (see also Figure 3). The second signal set in thiamyxin A and B was assigned a second conformer (C1 and C2), as only occurring in the cyclized derivatives. In addition, the same ratio of signal sets in different isolated batches strongly indicates a stable equilibrium of two conformers.

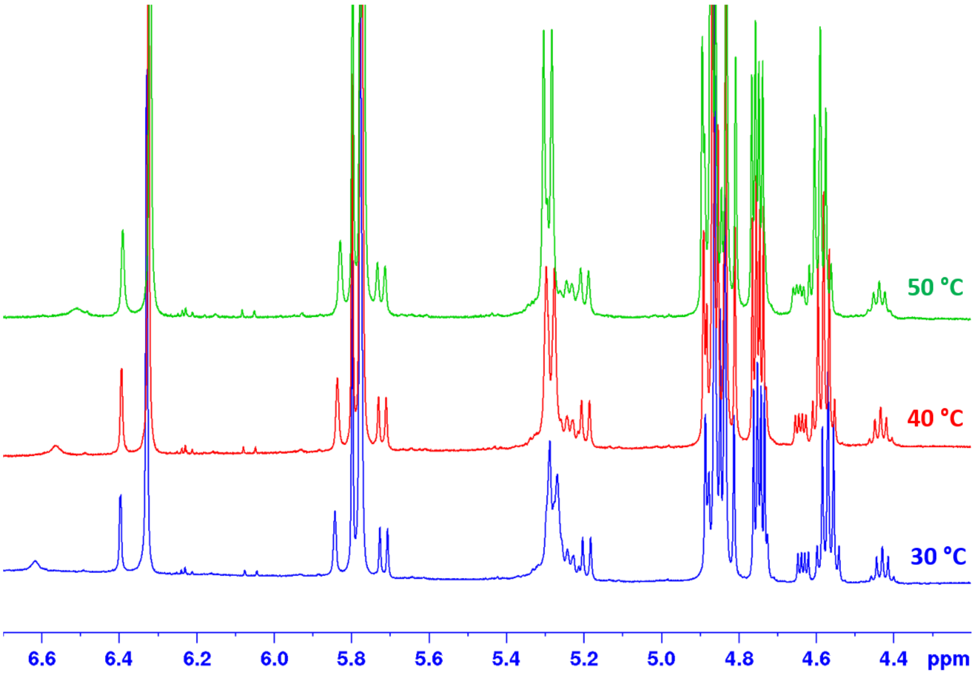

Figure 5 ^{1}H NMR section of thiamyxin B. Increasing measurement temperature does not cause a change in ration of different conformers.

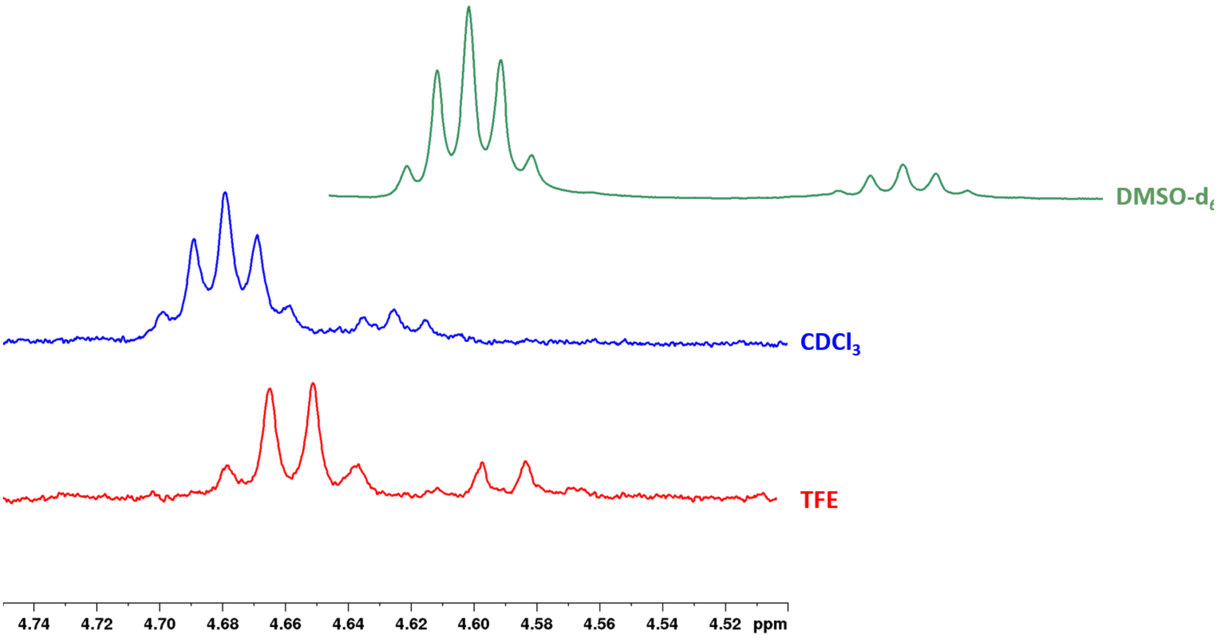

Figure 6 ^{1}H NMR section of thiamyxin B. Comparison of different solvents used for NMR measurements. No significant change in the ration of conformers was observed.

2.3 Stereochemistry assignment

The stereochemistry was assigned based using the Marfey's method.[5] Thiamyxin D was used for the stereochemistry assignment of Alanine, 4-methyl-thiazoline, O-methyl-serine and N-Methyl-valine. To attempt assignment of the isoleucine stereochemistry, thiamyxin A and B were hydrolyzed in addition to thiamyxin D and derivatized separately. The samples were analyzed using a longer gradient with improved separation for isoleucine and alloisoleucine. The retention times of references and thiamyxin hydrolysate and stereochemical assignment are summarized in table 1.

Table 2 Retention times and stereochemical assignment of the thiamyxin amino acids.

Reference	*L-FDLA*				*D-FDLA*				
Amino Acid	**Standard**	**TM D**	**TM A**	**TM B**	**Standard**	**TM D**	**TM A**	**TM B**	**configuration**
L-alanine	14.1	16.6	n/a	n/a	16.6	14.1	n/a	n/a	D/R
L-isoleucine	13.5	13.5	13.5	13.5	26.75	26.75	26.75	26.75	D/R – L/S
D-isoleucine	26.75	26.75	26.75	26.75	13.3	13.3	13.3	13.3	D/R – L/S
2-Me-Cysteine 1&2	10.4	10.4	n/a	n/a	9.6	9.6	n/a	n/a	L/R
O-Me-L-Serine	13.7	13.7	n/a	n/a	16.6	16.6	n/a	n/a	L/S
L-Valine	16.1	16.1	n/a	n/a	19.6	19.6	n/a	n/a	L/S

2.3.1 N-methyl-valine assignment

We were not able to detect N-methyl-valine after hydrolysis of the intact compounds, but instead observed a peak for L-valine. When subjecting the N-methyl-valine reference to the same conditions we were also able to detect Valine although in lower amounts (Figure 7). We assume that under acidic conditions the N-Methyl-Valine is demethylated and assigned the stereocenter as *S*-configured, in accordance with the detected L-Valine.

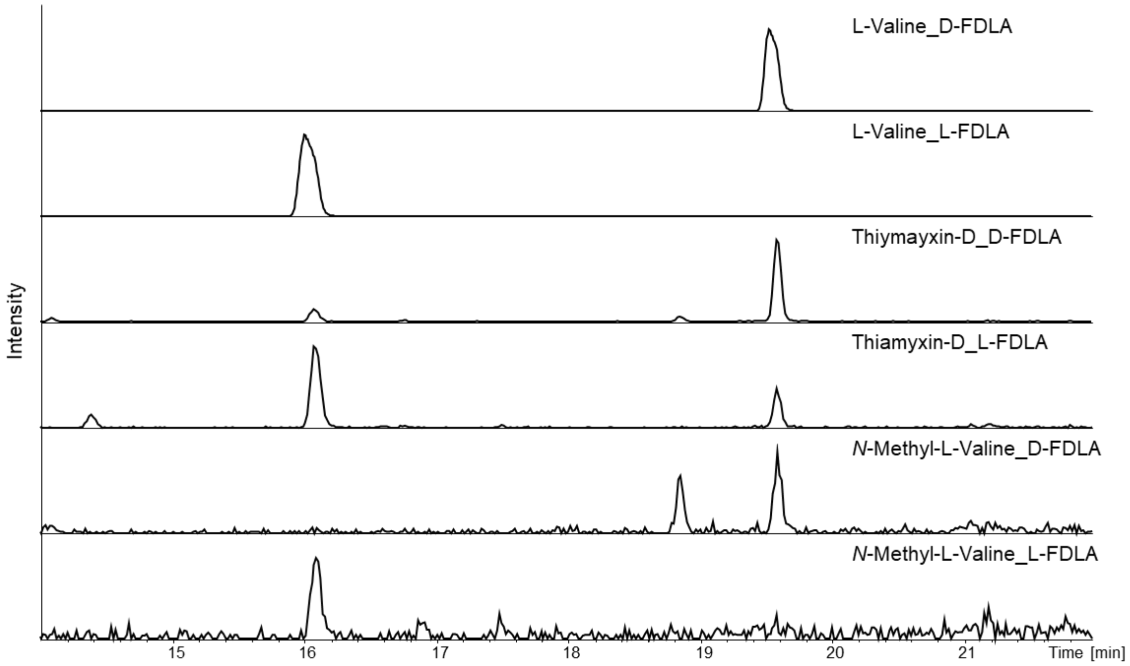

Figure 7 Extracted ion chromatograms for 412.183 *m/z* for derivatized L-Valine, thiamyxin D and N-methyl-L-valine.

2.3.2 Isoleucine assignment

To assign the stereochemistry of the isoleucine moiety, D- and L- isoleucine (I) as well as D- and L-alloisoleucine (AI) references were derivatized with D- and L- 1-flouro-2,4-dinitrophenyl-5-leucine-amide (FDLA). D-AI-D-FDLA, D-I-D-FDLA, L-AI-L-FDLA and L-I-L-FDLA have identical retention times,, as do D-AI-L-FDLA, D-I-L-FDLA, L-AI-D-FDLA and L-I-D-FDLA. When derivatized with D-FDLA, the thiamyxin A and B samples show peaks for D-AI-D-FDLA / D-I-D-FDLA and L-AI-D-FDLA / L-I-D-FDLA, however the peaks corresponding to D-AI-D-FDLA / D-I-D-FDLA are slightly larger. When derivatized with L-FDLA the thiamyxin A and B samples show peaks for L-I-L-FDLA / L-AI-L-FDLA and D-AI-L-FDLA / D-I-L-FDLA and again the peaks corresponding to D-AI-L-FDLA / D-I-L-FDLA are slightly larger. (Figure 8) We observe the corresponding peaks for D- and L- isoleucine for thiamyxin A as well as B due to a racemization reaction commonly occurring during acid hydrolysis for stereocenters adjacent to thiazoline moieties.[6] Even when extensively optimizing the reaction conditions, we were unable to fully retain Ile in its original configuration. As the differences in peak heights are not significant, we therefore were unable to assign D- or L-configuration of the thiamyxins based on this Marfeys analysis. Our NMR results however, clearly indicate thiamyxin B to incorporate Ile in *allo*-configuration (see Figure 3). We find an epimerization domain located in the isoleucine incorporating module so we assume the main product of the assembly line and all deriving shunt or degradation products (thiamyxin B, C and D) to incorporate D-*allo*-isoleucine. The high prevalence of L-Ile in natural products points towards its incorporation in thiamyxin A, but we cannot exclude a rather rare incorporation of D-Ile here. The resembling biological activities of thiamyxin A and B would reason them both to be D-configured, as this would result in a more similar conformation of the macrocycle. As the target of the

thiamyxins has not been identified yet, correlating their three dimensional structure with activity is highly speculative and only developing a total synthesis route for the thiamyxins will finally confirm the configuration of the Ile stereocenters.

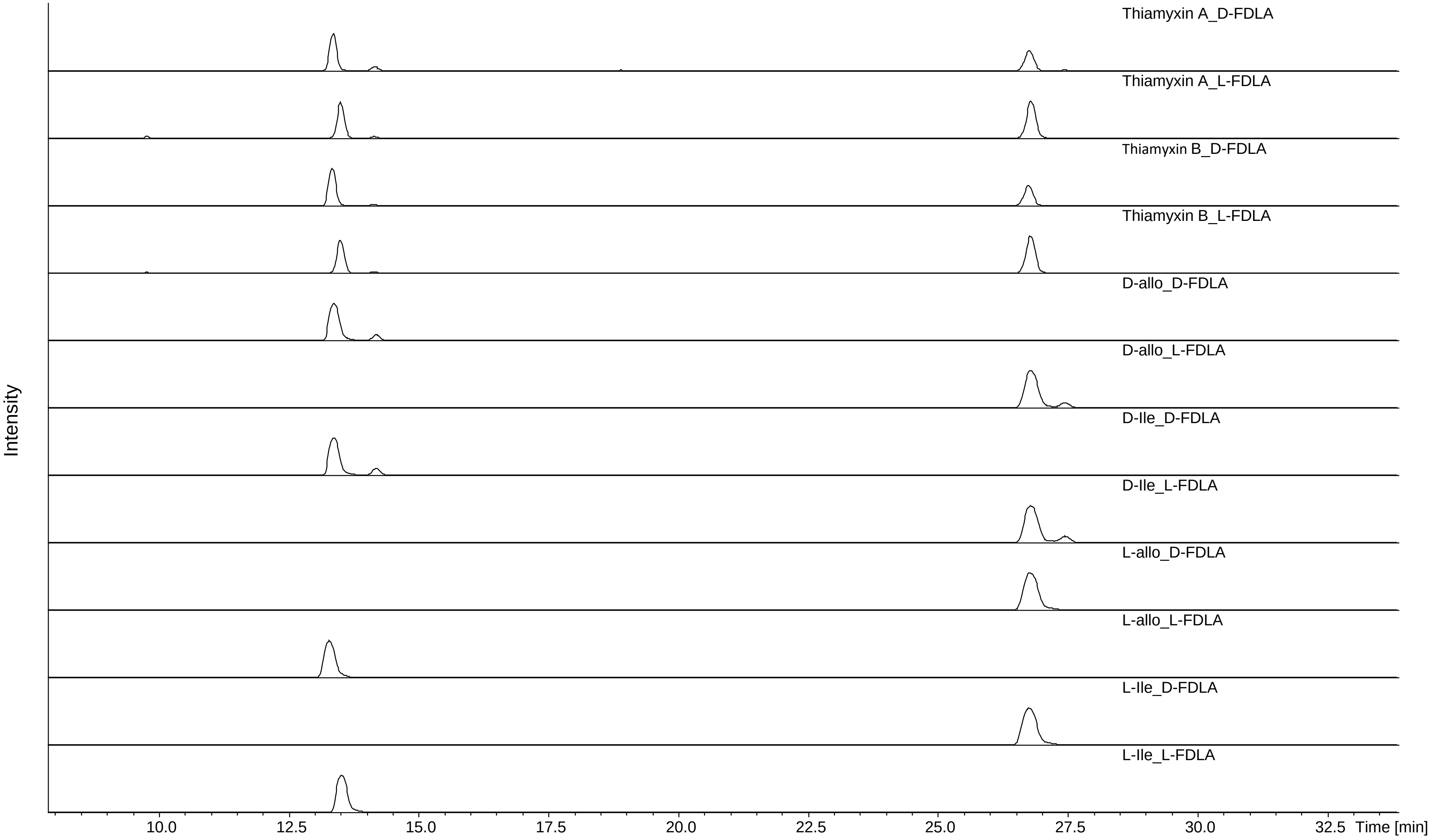

Figure 8 Extracted ion chromatograms for 426.198 *m/z* for derivatized D-alloisoleucine, D-Isoleucine, L-alloisoleucine, L-isoleucine and thiamyxins A and B.

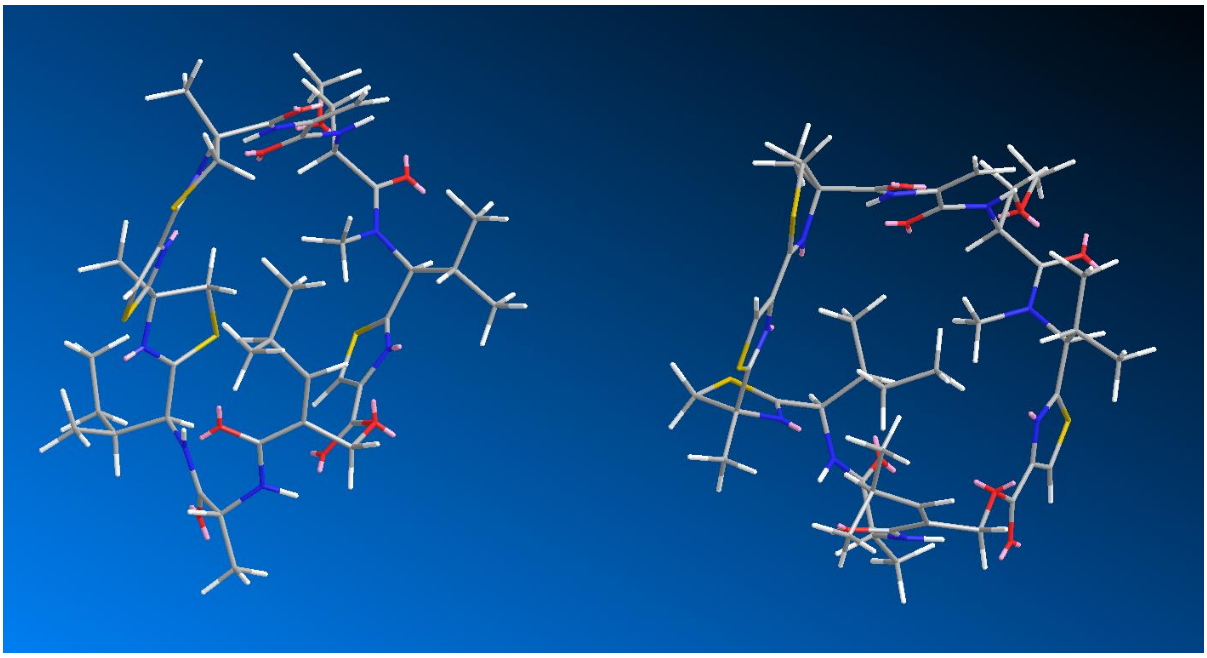

Figure 9 Lowest energy conformation of thiamyxin A (left) and thiamyxin B (right) as simulated using Chem3D (20.1.1).

2.3.3 Thiazoline assignment

To assign the stereochemistry of the 4-methyl-thiazoline stereocenters, we decided to use hydrolyzed thiangazole A as a reference due to inaccessibility issues of 2-methyl-cysteine. The peaks for 430.139 *m/z* of the hydrolyzed and derivatized samples of thiangazole A and thiamyxin D show identical retention times. As the stereocenters of the 4-methyl-thiazoline moieties in thiangazole A are all derived from *R*-configured 2-methylcysteine and there are no additional peaks with the relevant *m/z* in either sample we assign both 4-methyl-thiazoline stereocenters in the thiamyxins to be *R*-configured as well.

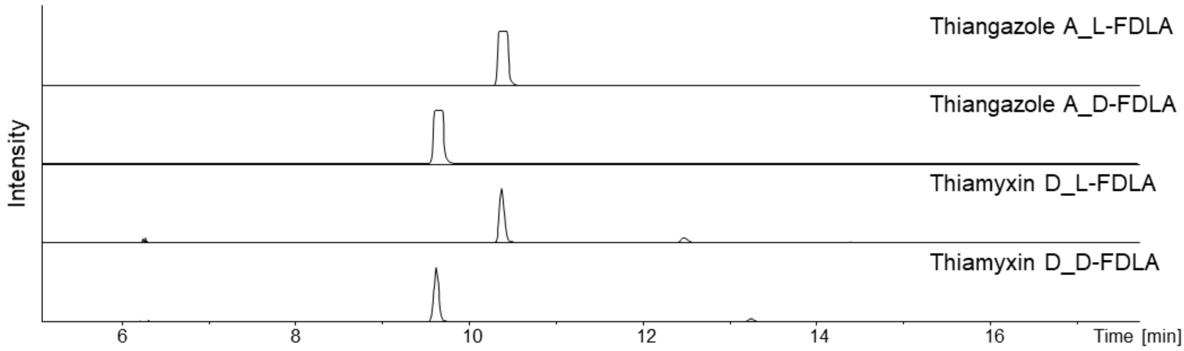

Figure 10 Extracted ion chromatograms for 430.139 *m/z* for derivatized thiangazole A and thiamyxin D.

2.3.4 Stability of thiamyxin A

To investigate if thiamyxin A can be converted to thiamyxin B, we treated thiamyxin A with pyridine, but could not detect any conversion. This finding supports our hypothesis that thiamyxin A indeed seems to be a side product of the assembly line rather than an artefact from the purification process.

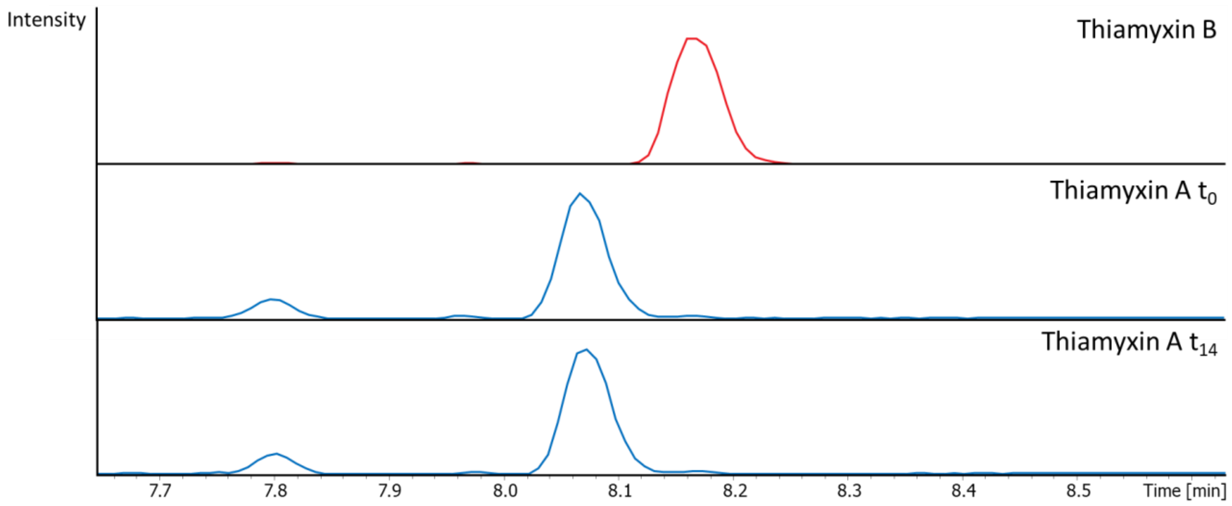

Figure 11 BPC of thiamyxin A (blue) in acqueous solution before base treatment (t_0) and after 14 h incubation at rt upon treatment with 5% pyridine. Thiamyxin B shown in red as a reference.

2.4 Gene cluster organization and proposed biosynthesis

Sequencing of the MCy9487 genome was performed as previously described for other myxobacteria.[2] The thiamyxin biosynthetic gene cluster was identified by submitting the MCy9487 genome to antiSMASH[7], analyzing the biosynthetic logic of the clusters and matching them to the elucidated structure. The pinpointed genes and closest orthologues can be found in Table 3. The orthologues were identified by BLAST[8] search based on the amino acid sequence of the query and the results table sorted by E-value. The top hit was chosen as representative orthologue. Most domains required for the biosynthesis hypothesis were identified by antiSMASH, however the oxidation domains in modules M7 and M12 as well as the O-methyltransferase domain in module M10 and the dimerization domain in module M1 were identified by submitting the corresponding sequences to CDD/SPARCLE[9]. The biosynthetic gene cluster with the corresponding annotations can be found under GenBank tag Thiamyxin_BGC_MCy9487 under GenBank accession number OP494098.

Table 3: Open reading frames in the thiamyxin biosynthetic gene cluster with proposed function and closest homologue.

**Name**	**Size [AA]**	**Proposed function / homologue**	**organism**	**Accession number of closest protein homologue**	**Sequence Identity [%]**
**Orf 1**	117	calmodulin	*Myxococcus fulvus*	WP_074958239.1	89.66
**Orf 2**	104	hypothetical protein	*Corallococcus terminator*	WP_147449015.1	62.14
**ThiA**	3022	polyketide synthase dehydratase domain-containing protein	*Corallococcus terminator*	WP_120539938.1	82.22

**ThiB**	3114	amino acid adenylation domain-containing protein	*Corallococcus terminator*	WP_120539939.1	84.04
**ThiC**	2025	AMP-binding protein	*Corallococcus terminator*	WP_147448653.1	84.85
**ThiD**	1393	amino acid adenylation domain-containing protein	*Hapalosiphon sp. MRB220*	WP_053457693.1	51.31
**Orf 3**	107	hypothetical protein	*Corallococcus terminator*	WP_120544219.1	61.95
**Orf 4**	396	TRC40/GET3/ArsA family transport-energizing ATPase	*Corallococcus terminator*	WP_120544218.1	96.97
**ThiI**	413	cytochrome P450	*Corallococcus terminator*	RKG77511.1	87.89
**ThiJ**	319	alpha/beta fold hydrolase	*Corallococcus terminator*	WP_158625188.1	84.64
**ThiE**	1555	amino acid adenylation domain-containing protein	*Corallococcus terminator*	WP_120544215.1	88.24
**ThiF**	2499	amino acid adenylation domain-containing protein	*Archangium primigenium*	WP_204490555.1	49.15
**ThiG**	2055	amino acid adenylation domain-containing protein	*Corallococcus terminator*	WP_120544021.1	85.29
**ThiH**	742	amino acid adenylation domain-containing protein	*Corallococcus terminator*	WP_120544021.1	83.11
**Orf 5**	940	type I polyketide synthase	*Corallococcus terminator*	WP_120544022.1	81.06
**Orf 6**	336	patatin-like phospholipase family protein	*Corallococcus terminator*	WP_120544023.1	87.99
**Orf 7**	333	radical SAM protein	*Corallococcus terminator*	WP_120543822.1	94.89
**Orf 8**	182	NUDIX domain-containing protein	*Corallococcus sp. ZKHCc1 1396*	WP_193348944.1	87.43
**Orf 9**	296	hypothetical protein	*Corallococcus terminator*	WP_120543827.1	87.50

2.4.1 Active site residues of module 5 (*thiC*)

Module 5 consists of a condensation and peptidyl carrier protein (PCP) domain. As there is no biosynthetic step assigned to this module, yet it the C domain contains an intact active site, we propose that its function is assisting the loading the nascent molecule onto the PCP-domain of the next module.

```
Feature 1
query            9 IYPLSPSQQGMLYESLA.[5].IHVEQLVWRM.[1].GPLDEA.[1].LERAWQQVVDRH.[1].VLRTCF.[4].QSEP 75
NP_624809     1194 ILPLTPLQEGLYFHSVY.[6].SYVEQQLLTL.[1].GEVDPG.[1].LAAAATRLLTLH.[1].NLAARF.[4].DGRV 1261
NP_251114     3250 VYPLTPMQEGLLLHTLL.[5].IYYMQDRYRI.[1].SPLDPE.[1].FAAAWQAVVARH.[1].ALRASF.[4].GETM 3316
Q939Z0        3022 IWPLSPLQEGLLFHAAD.[5].VYASMRTLAI.[1].GPLDVA.[1].FRASWTVLLDRH.[1].ALRASF.[4].SGEA 3088
WP_012266627    15 IYPLSPMQEGMLFHSLY.[5].IYCSQTLITL.[1].GEINLA.[1].FRQAWEKVVERH.[1].VLRTLF.[4].REKP 81
WP_012267918    17 IYPLSPMQQGMLFHSLY.[5].TYLSQIQITL.[1].GNLDIN.[1].FQQAWQKVVDRH.[1].ILRTCF.[4].TKQP 83
O68008        5091 IYPLANMQRGMLFHALE.[5].AYFEQMAINM.[1].GLIDER.[1].FAETFNDIMERH.[1].ILRASI.[4].TDEP 5157
Q70LM6        2584 VYTLTPLQEGMLFHSLY.[5].DYVVQLALKL.[1].HVNVEA     FSAAWQKVVERH.[1].ILRTSF.[4].LEKP 2649
Q04747           9 VYALTPMQEGMLYHAML.[5].SYFTQLELGI.[1].GAFDLE.[1].FEKSVNELIRSY.[1].ILRTVF.[4].LQKP 75

Feature 1                                                                                        ##   #
query           76 VQVVL.[2].VSVTLRSHDL.[11].LEAELE.[1].DRLRGFKP.[2].APLMKLSLFR.[4].EARLVWTFHHLLLDG 149
NP_624809     1262 VSVLE.[2].REAPFTVLDR.[ 7].IRAHAE.[1].DRRAGFDL.[2].GPPMRYTLIR.[4].RHVLVQTVHHIVADG 1331
NP_251114     3317 LQVIH.[2].GRTRIEFLDW.[11].LQALHK.[1].EREAGFDL.[2].QPPFHLRLIR.[4].RYWFMMSNHHILIDA 3390
Q939Z0        3089 VQVIA.[2].VPPDWRETDL.[11].FDRLAA.[1].MHAERFDL.[2].APQLRLHLVR.[4].RYRLIFTSHHIVADG 3162
WP_012266627    82 LQIVR.[2].VDLPWDYQDW.[11].LDLLLE.[1].ERQQGFEL.[2].APLMRCLMIQ.[4].TYKFLCNHHHIILDG 155
WP_012267918    84 LQVVR.[2].VTLPWFNQDW.[11].FQELLT.[1].DKEQYFEL.[2].VPLIRCHLIR.[4].KYEFIHTGHHILLDG 157
O68008        5158 RNVII.[2].RKINLDYHDL.[11].IQAYRK.[1].DREKGFRL.[2].EPLIRAALMR.[4].SYTFIWTNHHILLDG 5231
Q70LM6        2650 HQVVH.[2].VKTFVERLDW.[11].LQTYLE.[1].DRKRGFDL.[2].PPLMRWTLIR.[4].TFQFVWSFHHMLLDG 2723
Q04747          76 RQVVL.[2].RKTKVHYEDI.[11].IERYKQ.[1].VQRQGFNL.[2].DILFKVAVFR.[4].QLYLVWSNHHIMMDG 149

Feature 1
query          150 WCLPLLLQEVLSCY.[13].SRPFRDYVAWLKR.[2].PEAARTFWAERLRA.[3].PTPLG.[ 4].QGEPG.[1].G 224
NP_624809     1332 WSVPPMLRTLLAEY.[ 8].LGGFPEHVRRLAA.[2].GAASDRVWDEQLAD      LPGPS      LIAEG     H 1393
NP_251114     3391 WCRGLLMNDFFEIY.[13].PPRYRDYIAWLQR.[2].LEQSRRWWSESLRG.[2].RPTLV.[10].AGESG.[1].M 3470
Q939Z0        3163 WSLPLILVDVLTAY.[11].ATSYRDFLAWVDR.[2].KGAAGQAWRTELAG.[2].EATHV.[ 3].GSIIT.[1].L 3233
WP_012266627   156 WSMPIIYQEVLGFY.[13].PRPYQDYIVWLQQ.[2].PSIAESFWQRTLEG.[3].PTPLR.[ 6].MKSEG.[1].P 232
WP_012267918   158 WATAILLKEVFDFY.[13].PRPYQDYINWLQQ.[2].QTEAERFWRKNLQG.[3].PTPLV.[ 6].PLVTQ.[1].K 234
O68008        5232 WSRGIIMGELFHMY.[13].ARPYSDYIGWLQQ.[2].KEAAKAYWRNYLSG.[3].KSPIS.[ 4].SSGHA.[1].Y 5306
Q70LM6        2724 WSTPIVFQDWQAFY.[13].IPPFSAYIAWLKR.[2].LEEAQQYWRDYLQG.[3].PTPLG.[ 5].GSAGQ.[1].K 2799
Q04747         150 WSMGVLMKSLFQNY.[13].GKPYSDYIKWLGK.[2].NEEAESYWSERLAG.[3].PSVLP.[ 3].PVKKD.[1].Y 223
```

Figure 12 Alignment of the C domain of module 5 (*thiC*) amino acid sequence (query) with reference sequences from CDD/SPARCLE database. The active site histidines and aspartic acid of the characteristic HHxxxD motif are highlighted in yellow.

2.4.2 Active site residues of orf 5

Orf 5 is located downstream of thiH of the thiamyxin gene cluster. It contains a ketosynthase (KS) and PCP domain. The KS domain is missing the first active site histidine (Figure 13). We were not able to assign a biosynthetic function to these domains and therefore presume them to be inactive.

```
Feature 1                          #
query         158 GPSLGVQTACSTSLVAVHLACQSLLSG.[2].DMALAGAVSM.[5].RGYLHEEGSSLS.[4].CRAFDARANGTPFGSG 233
AAC38075      174 GPAMTVTTACSSSLVAMHLACRALQAG.[2].DMALAGGVNL.[5].LTIYMSQIRAIS.[4].CRVFDAAADGIVRGEG 249
AAB08104      177 GPSVLVDTACSGGLTALHLACQSLLVG.[2].RQALAAGSSL.[5].MMVTMSMMKFLS.[4].CYAFDERANGYARGEG 252
AAB53258      174 GPSYTVDSACSSSLYALEHAFRAIRDG.[2].DAAIVGGSNL.[5].VSLQFSRLGVLS.[4].CKSFDNSANGYARSEA 249
CAB06094      175 GPSIAVQTACSSSLVAVHLACLSLLSG.[2].DMALAGGSSL.[5].VGYFTSPGSMVS.[4].CRPFDVRADGTVFGSG 250
AAP42872      206 GPTATLDTACSGSLVALHLACQSLRGG.[2].SMALAGGVTV.[5].TFIGTGRGIGLP.[4].CRSFADGAEGIAFAEG 281
CAD19086      194 GPSIVVDTACSSSLVAVHLACQSLRSK.[2].DLAIAGGANL.[5].WSVAISKLQALA.[4].CKTFDARADGFVRAEG 269
CAE14178      201 GPSLQIDTACSSGLTALTQAVNSLRSG.[2].QQAIVGSVNL.[5].NMAAYYRAGMLS.[4].CRVFDADANGFVRGEG 276
AAF00958      197 GPCLSIDTACASSLVAVHQGIRSLRNR.[2].ELALVGGVNL.[5].ITISLSQSGMMS.[4].CKTFDASANGYVRGEG 272
BAB12210     2281 GPSMTIDTACSSSLVAIHLAYNALLNG.[2].DLALAGGVNI.[5].ISLIESRAHMLA.[4].CKTFDESANGMVRGEG 2356

Feature 1                                                                           #
query         234 LGVVILKRLSEA.[6].IHAIIKGSALNNDGALKVGY.[4].GDSQARVIREALA.[7].SISYVEANGTGTPLGDTIEL 315
AAC38075      250 CGVTVLKRLADA.[6].IQAVIRGSAINQDGASAGQT.[3].ANAQAAVISQALK.[7].DIDYVEAHGTGTPLGDPIEL 330
AAB08104      253 VAVLLLKRLEDA.[6].IRAVIRGTGCNQDGKTPGIT.[3].SVSQEALIRSVYK.[7].DTTYVECHGTGTQAGDTTEA 333
AAB53258      250 IVVCFLQKAKDS.[2].VYAQLLHAKTNCDGYKEQGI.[4].GHIQKLLLREFYE.[7].ELEFVEAHGTGTRVGDPEEL 327
CAB06094      251 VGLVVLKPLAAA.[6].IHAVIRGSAINNDGSAKMGY.[4].PAAQADVIAEAHA.[7].TVSYVECHGTGTPLGDPIEI 332
AAP42872      282 AGVVLLERLSTA.[6].VLAVVRGSAIGQEGTNNGVS.[3].GPAQQRLIRQALA.[7].EIDAVEGQGTGGLLSDAVEA 362
CAD19086      270 CGTIVLKRLSDA.[6].IRALIRGSATNQDGHSQGLT.[3].GLTQQALLRQALQ.[7].QVSYIETHGTGTILGDPIEV 350
CAE14178      277 AICLFLKTQKQA.[6].IYGYVRASAVNHGGRANSLT.[3].PEQQIALVKDCLL.[7].QISYLEAHGTGTSLGDPIEF 357
AAF00958      273 CGVLILKTLSEA.[6].ILALLRGSAVNHNGAAAGLT.[3].GPAQQELLRQALA.[7].DVSYIEAHGTGTSLGDPIEL 353
BAB12210     2357 CGIVVLKRLSQA.[6].ILAKIYGTAVNHDGPSSGLT.[3].GQAQEKLLHQALK.[7].QIDYIEAHGTGTALGDPIEL 2437

Feature 1                                  #
query         316 SALNRAF.[10].ALGSVKTNVGHLSVASGMAGLIKTVLALE.[1].QQLPASLNF.[17].TGLKGWP.[5].RRAGVS 406
AAC38075      331 SSLDSAF.[ 7].WVGSVKANMGHLDAAAGMASVIKTMMVLK.[1].AEVPAQLHL.[17].TAIESLP.[3].RLAGIS 416
AAB08104      334 SALSKVF.[ 8].LIGSVKTNIGHLEGASGLAGVVKSILMLE.[1].GVILPNRNF.[16].PTTLECW.[5].RRVSIN 421
AAB53258      328 LAIDEIF.[ 8].LLGSIKSNLGHSEPASGLCSIAKMCIAYT.[1].GYIPPNLNY.[14].MNVITDK.[5].GMSGIN 413
CAB06094      333 QGLRAAF.[11].VLGSVKSNIGHLEVAAGIAGLIKTILCLK.[1].KALPATLHY.[17].SKYGPWE.[4].RRAGVS 423
AAP42872      363 QALASVY.[10].LLGAVKSNLGHTQGASGVAGVIKTVQAMR.[1].GVLPRTLHT.[17].TAATPWP.[5].LRSGVS 453
CAD19086      351 SALSEVY.[10].ILGSVKTNVGHLEAAAGIAGLLKVVLALE.[1].GAVPKQLHF.[17].TEMSPWP.[5].RMGAVS 441
CAE14178      358 NALNEVF.[12].YIGSVKANIGHLEGAAGLAGIVKVLLMLQ.[1].KSIVPSAAF.[17].TEENSWR.[6].RFAGLS 451
AAF00958      354 NAIASVY.[ 7].YVASVKTNIGHLEAAAGMAGIIKTILILQ.[1].GEIPPHLHF.[17].TQNIPWP.[5].PIAGVS 441
BAB12210     2438 ESMSAVF.[ 9].IIGSVKTNLGHLEGAAGIAGLIKTVLALQ.[1].HKIPPHLHF.[17].VQGKPWD.[5].RIAGVS 2527
```

Figure 13 Alignment of the orf 5 KS Domain amino acid sequence (query) with reference sequences from CDD/SPARCLE database. The active site cysteine and histidines are highlighted in yellow.

2.4.3 Dimerization domain in thiA

A dimerization domain (red) commonly found within methyltransferase domains was identified upstream of the GNAT domain in module M1 of the thiamyxin gene cluster by submitting the aminoacid sequence of thiA to CD-search (Figure 14).

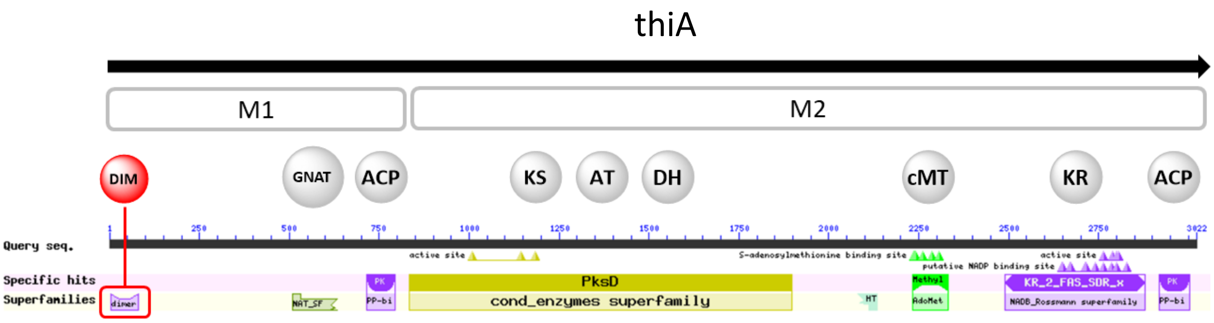

Figure 14 Output of the CDD/SPARCLE conserved domain search of thiA. The dimerization domain is marked in red, other domains in grey.

2.4.4 Alignment of GNAT Domains

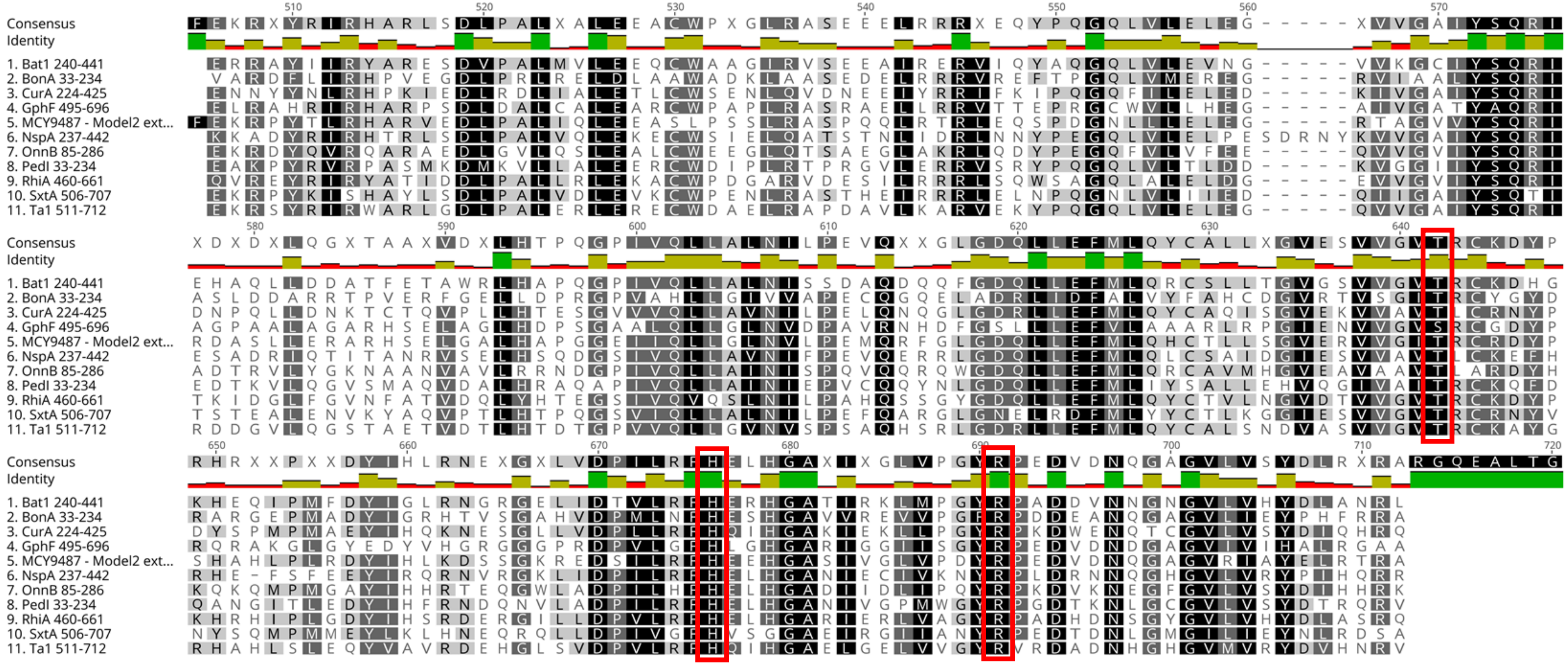

Figure 15 Alignment of GNAT domains from the thiamyxin (MCY9487 – Model 2), Gephyronic acid (GphF), Curacin A (CurA), Batumin (Bat1), Bongkrekic acid (BonA), Nosperin (NspA), Onnamide (OnnB), Pederin (PedI), Rhizoxin (RhiA), Saxitoxin (SxtA) and Myxovirescin A (Ta1) gene cluster, as performed by Skiba *et al.*[10] The conserved histidine, arginine and threonine required for decarboxylation are marked in red.

2.4.5 Phylogenetic analysis of C domains

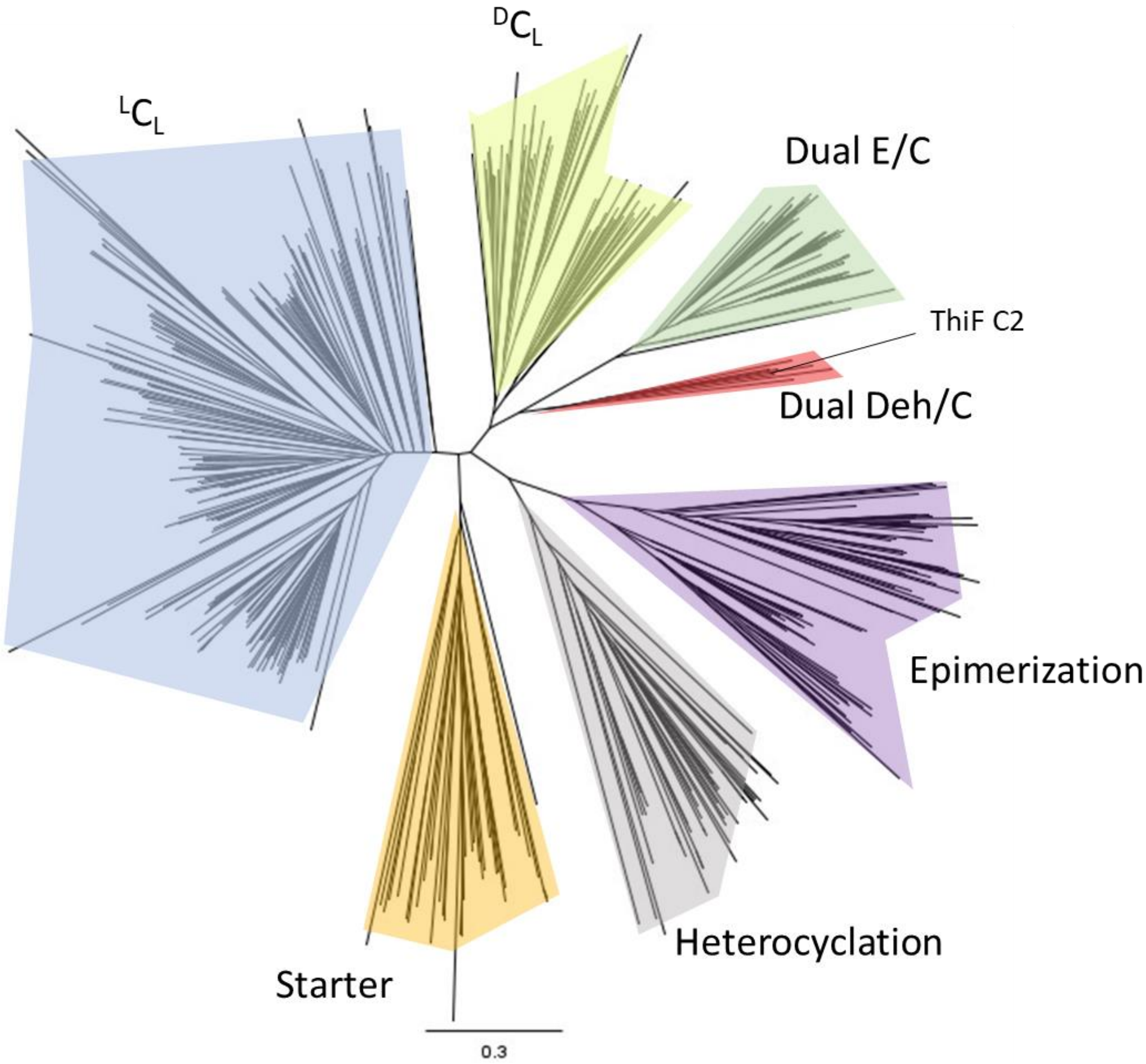

Figure 16 Phylogenetic tree of different C domain subtypes as previously described by Pogorevc et al.[11] Phylogenetic tree of all known C domain subtypes (LCL, DCL, Starter, Dual E/C, Epimerization and Heterocyclization domains), with the new Dual Deh/C subtype highlighted in red. The phylogeny was reconstructed using phyml, employing the JTT model of amino acid substitution and a gamma-distributed rate variation with four categories. The support values are based on 100-fold bootstrapping. The C domain list includes 525 domains from phylogenetic study by Rausch et. al.,[12], 10 C domains from the thiamyxin biosynthesis, as well as selected C domain examples from α,β-dehydro amino acid forming pathways: bleomycin (blmVI; Q9FB23), burriogladin (BgdA; MH170348), haerogladin (HgdA, MH170356), nocardicin (NocB; Q5J1Q6), hassallidin (HasO; K7VZQ9), syringomycin (SyrE; O85168), stenothricin (StenS; EFE73312.1). The C domain of module 10 of the thiamyxin biosynthesis encoded on *thiF*, belongs to the clade of dehydrating C domains.

2.5 Feeding Experiments

The isotopic patterns of the thiamyxins for the serine-d_3 supplemented cultures show intensified peaks at +2 Da, +3 Da, +4 Da and +5 Da compared to the monoisotopic peak, indicating 2 deuterium-labelled serine incorporations, one of which is reduced to dehydroalanine. The isotopic pattern of the thiamyxins for the Valine-d_8 supplemented culture show intensified peaks at +7 Da, +8 Da and +9 Da compared to the monoisotopic peak, indicating a deuterium-labelled valine incorporation. The alpha deuterium is acidic and exchanges with hydrogen in aqueous conditions which causes the +7 Da signal.

The isotopic patterns for the methionine-(methyl-^{13}C) supplemented cultures show intensified peaks at +1 - +9 Da, indicating up to 7 methylations (see Figure 17).

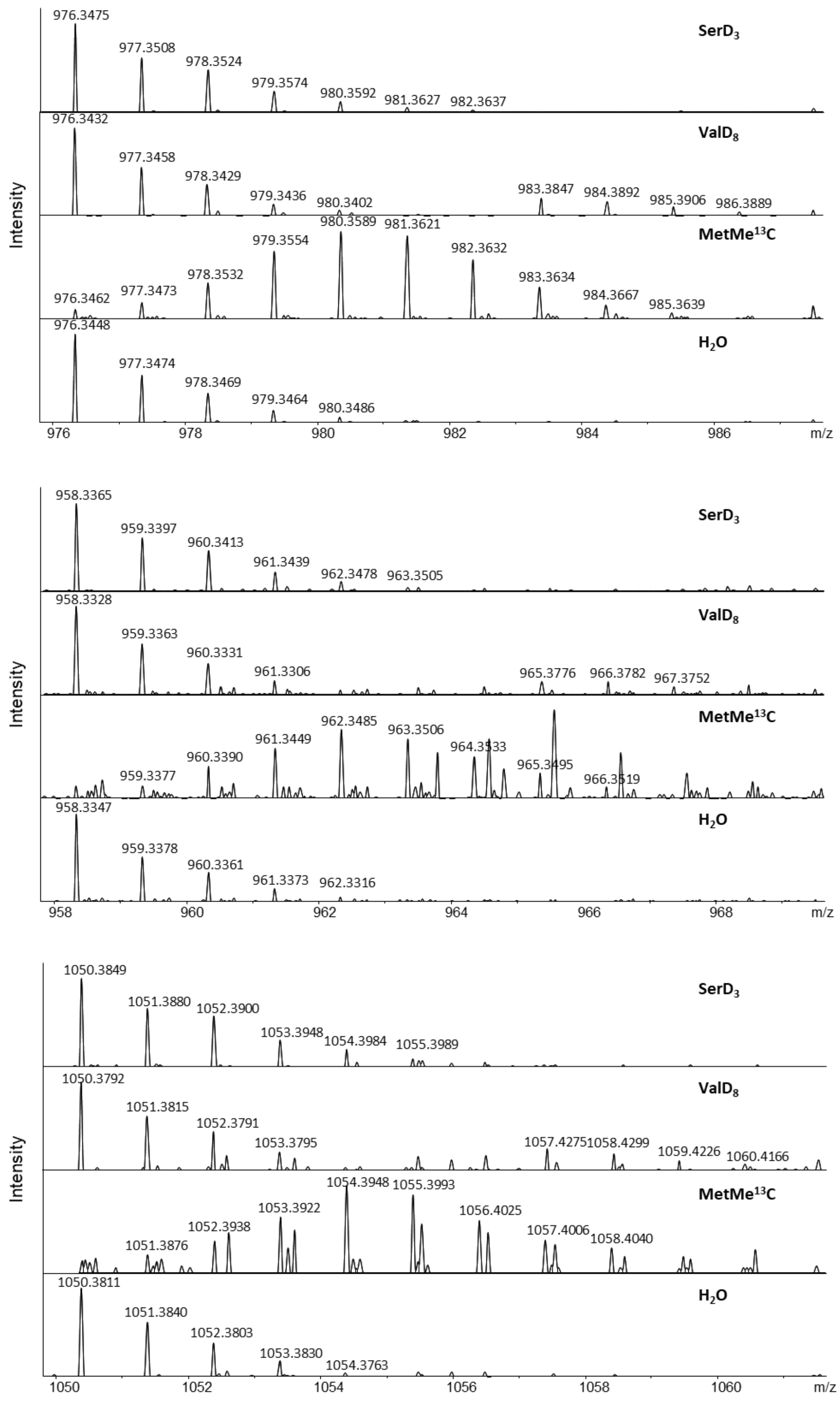

Figure 17 Zoomed in mass spectra for thiamyxin C (top), thiamyxin A/B (middle) and thiamyxin D (bottom) with L-serine-2,3,3-d$_3$, L-valine-d$_8$, and L-methionine-(methyl-^{13}C) feeding and control.

2.6 Antimicrobial activities

All thiamyxins except congener A, which was only produced in low amounts, were tested against two Gram-positive and two fungal pathogens. They were only found to have weak effects on our test panel with MIC values higher than 32 µg/mL (see Table 4).

Table 4 Antimicrobial activities of the thiamyxins.

**Test organism**	**MIC [µg/mL]**			
	thiamyxin A	thiamyxin B	thiamyxin C	thiamyxin D
***Bacillus subtilis* DSM-10**	nd	> 64	64	> 64
***Mucor hiemalis* DSM-2656**	nd	> 64	64	> 64
***Candida. albicans* DSM-1665**	nd	64	> 64	> 64
***Micrococcus luteus* DSM-1790**	nd	> 64	32	> 64

2.7 Antiviral activities

Half maximal inhibitory concentrations (IC_{50}) values presented in the main text were calculated based on the following curves determined for the inhibition of the respective virus (Figure 18-25). Half maximal cytotoxic concentration (CC_{50}) values presented here were determined simultaneously in the infected cell lines to see if the observed antiviral effects are overlapping with effects on the cell lines. IC_{50} values shown in the manuscript were determined in an independent experiment to circumvent effects of the viral infections on the cell lines.

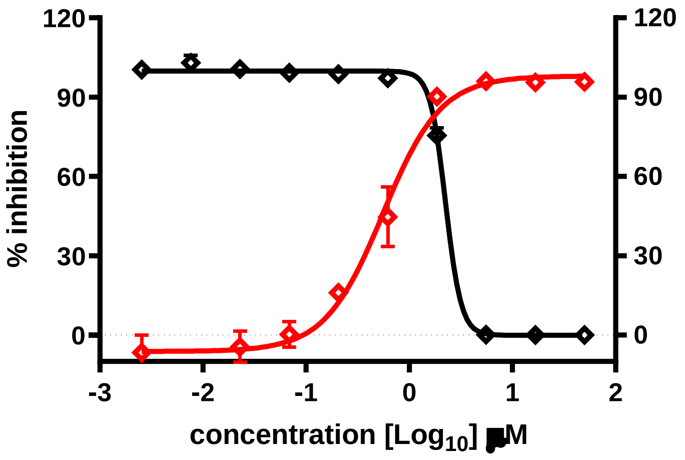

Figure 18 Inhibition of DENV-R2A (red, IC_{50} = 0.56 µM) and effects on cell viability (black, CC_{50} = 2.25 µM) of thiamyxin B.

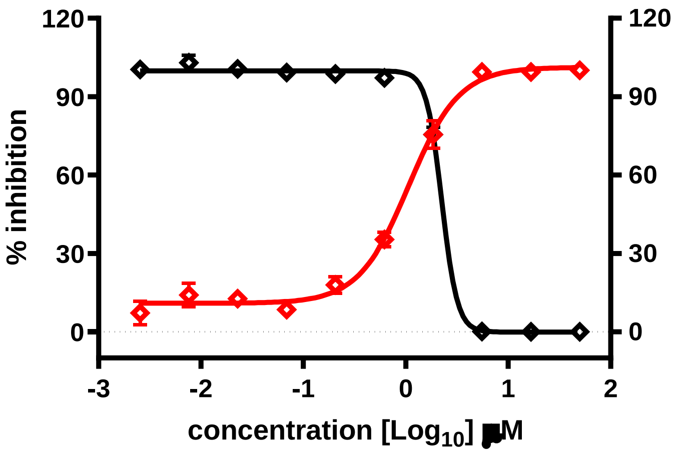

Figure 19 Inhibition of ZIKV-R2A (red, IC_{50} = 1.07 µM) and effects on cell viability (black, CC_{50} = 2.25 µM) of thiamyxin B.

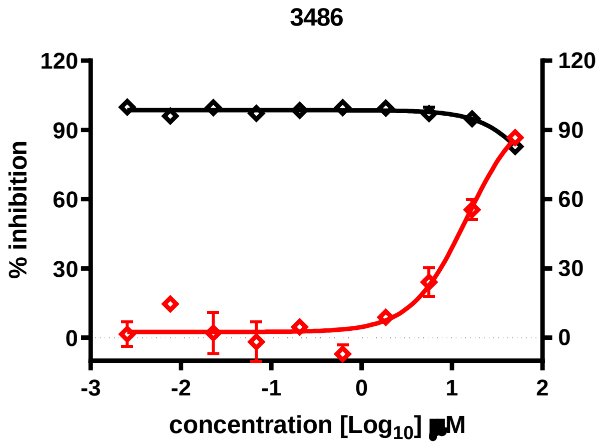

Figure 20 Inhibition of DENV-R2A (red, IC_{50} = 14.56 µM) and effects on cell viability (black, CC_{50} = >50 µM) of thiamyxin C.

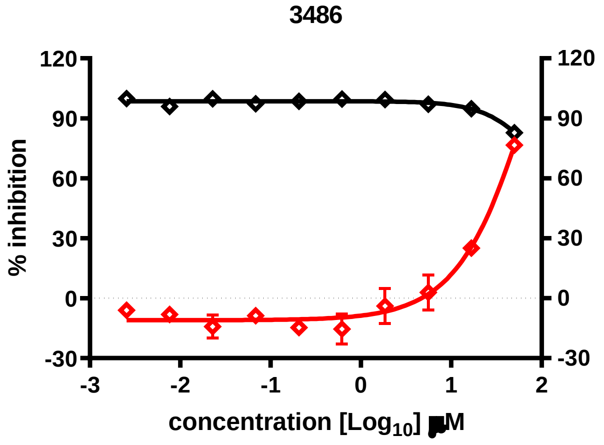

#

Figure 21 Inhibition of ZIKV-R2A (red, IC_{50} >15 µM) and effects on cell viability (black, CC_{50} >50 µM) of thiamyxin C.

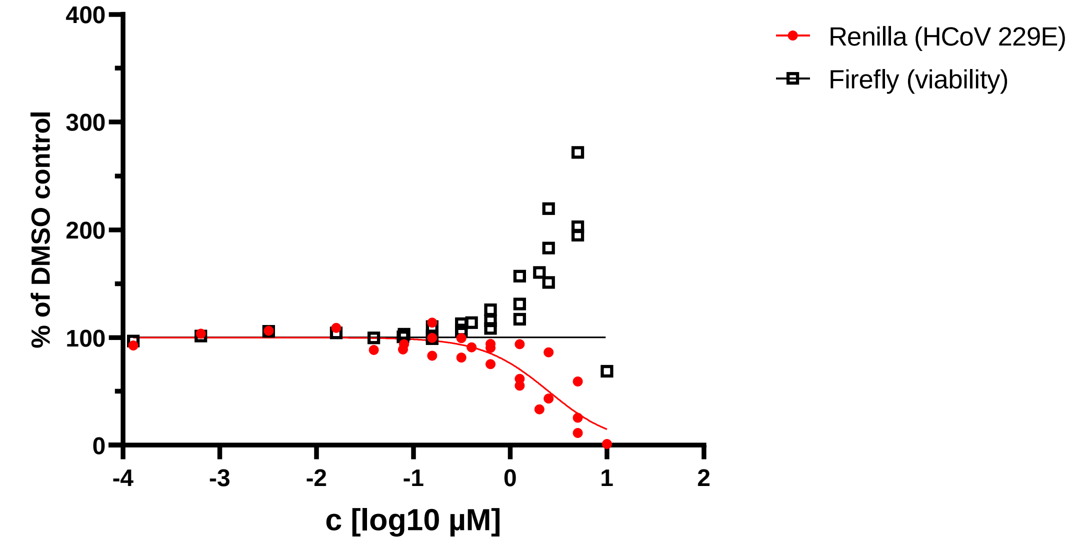

Figure 22 Inhibition of hCoV-229E-luc (red, IC_{50} = 2.47 µM) and effects on cell viability (black, CC_{50} >10 µM) of thiamyxin A.

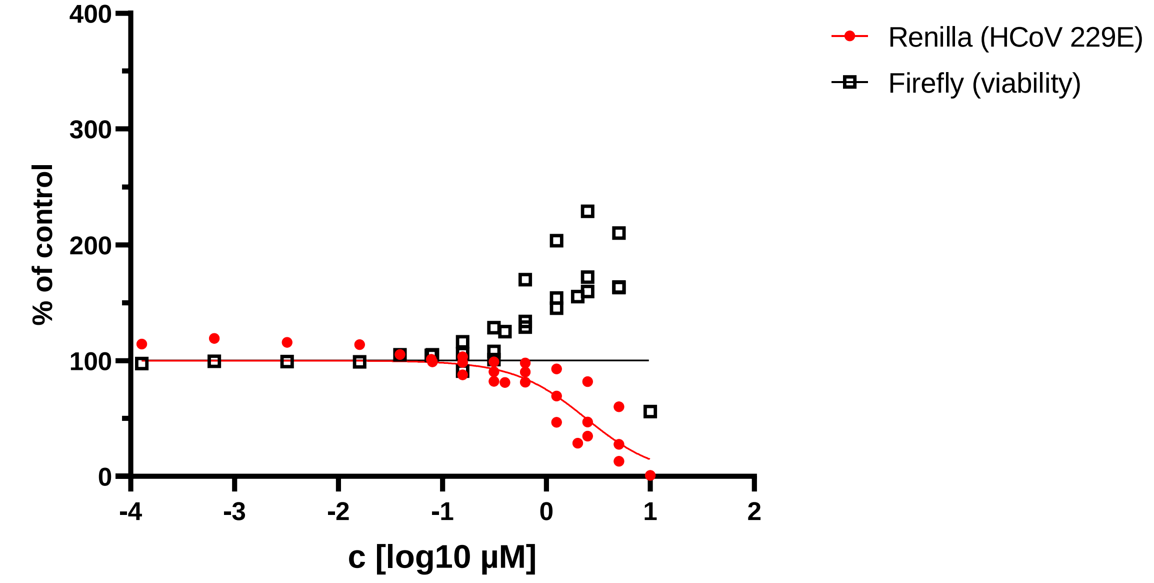

Figure 23 Inhibition of hCoV-229E-luc (red, IC_{50} = 2.39 µM) and effects on cell viability (black, CC_{50} >10 µM) of thiamyxin B.

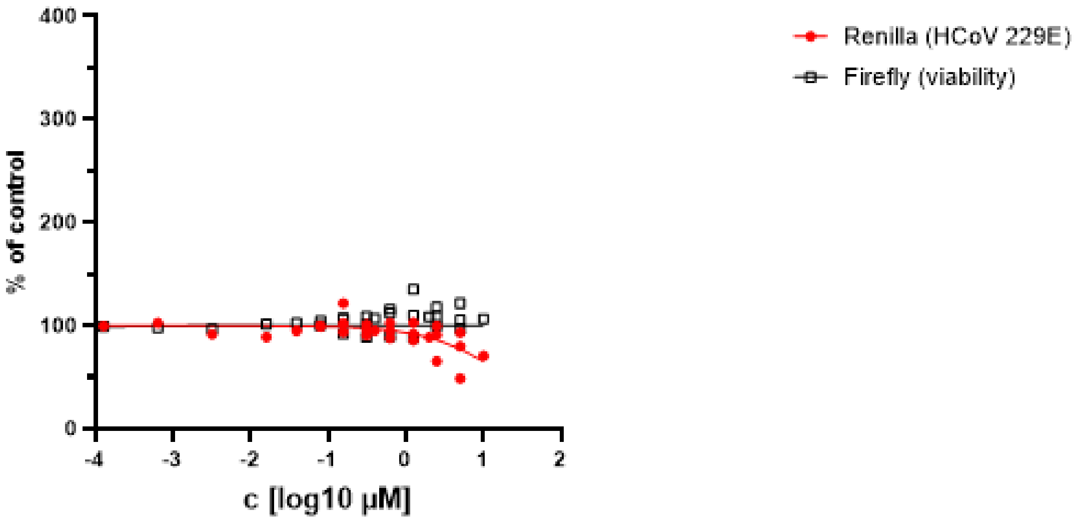

Figure 24 Inhibition of hCoV-229E-luc (red, IC_{50} ~ 20.52 µM) and effects on cell viability (black, CC_{50} >10 µM) of thiamyxin C.

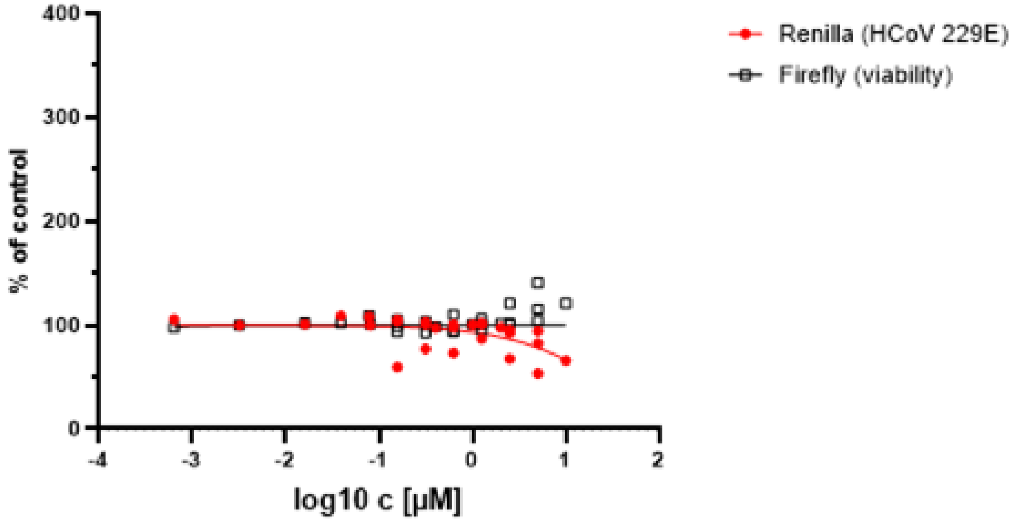

Figure 25 Inhibition of hCoV-229E-luc (red, IC_{50} ~ 23.06 µM) and effects on cell viability (black, CC_{50} >10 µM) of thiamyxin D.

2.8 NMR data

2.8.1 Thiamyxin B

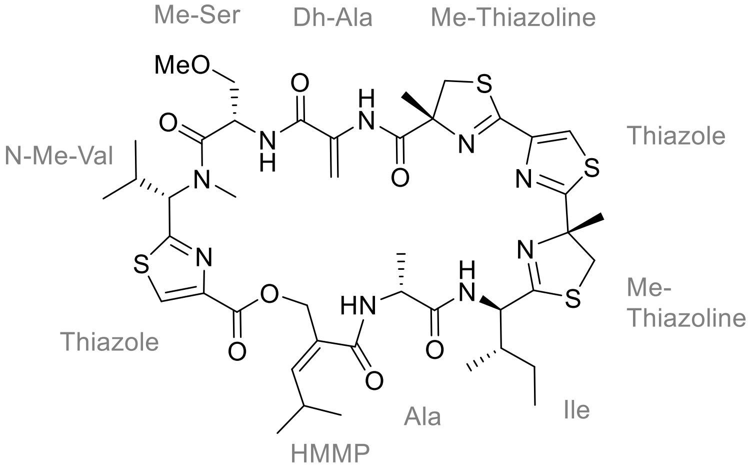

Table 5 NMR spectroscopic data of thiamyxin B in DMSO-d_6 at 700/175 MHz.

	NMR data in DMSO-d_6			
position	δ_C	δ_H (J in Hz)	COSY correlations	HMBC correlations
HMMP				
1	165.8	-	-	-
2	129.2	-	-	-
2’	67.0	4.90, 4.86, d (12.1)	3	1, 2, 3, *Thiazole*-1
3	146.6	5.83, s	2’, 4	1, 2, 2’,4, 4’, 5
4	27.8	2.83, dq (16.4, 6.7)	3, 4’, 5	2, 3, 4’, 5
4’	22.2	0.96, d (6.6)	4, 5	3, 4, 5
5	22.2	0.96, d (6.6)	4, 4’	3, 4, 4’
Ala				
1	171.6	-	-	-
2	47.6	4.61, quin (7.0)	3, NH	1, 3, *HMMP* -1
3	19.4	1.36, m*	2	1, 2
NH	-	7.51, bd (7.7)	2	-
Ile				

1	173.1	-	-	-
2	53.6	4.78, dd (9.4, 4.7)	3, NH	1, 3, 3', 4, *Ala*-1
3	37.5	1.91, m	2, 3', 4	1, 2, 3', 4, 5
3'	14.2	0.93, dd (10.5, 6.8)	3	2, 3, 4, 5
4	25.9	1.36, 1.21, m*	3, 5	3' ,3 ,5
5	11.4	0.87, t (7.4)	4	3, 4
NH	-	8.30, m*	2	2, *Ala*-1
Me-thiazoline				
1	176.5	-	-	-
2	83.3	-	-	-
3	43.3	3.74, dd (11.8, 39.7)	-	1, 2, Me, *Ile*-1
Me	28.3	1.65, s	-	1, 2, 3
Thiazole				
1	163.8	-	-	-
2	147.5	-	-	-
3	122.5	8.30, s*	-	1, 2, *Me-thiazoline*-1
Me-thiazoline				
1	*172.9*	-	-	-
2	*84.9*	-	-	-
3	*40.7*	*3.38, 3.81, d (11.7)*	-	*1, 2, Me,* Thiazole-1
Me	*25.5*	*1.59, s*	-	*1, 2, 3*
Dh-Ala				
1	163.1	-	-	-
2	132.7	-	-	-
3	102.6	6.37 *s*, 5.81 *s*	NH	1, 2
NH	-	9.36, s	3	1, 3, *Me-Thiazoline-1*

Me-Ser				
1	170.6	-	-	-
2	49.5	4.89, m*	3, NH	1, 3, *Dh-Ala*-1
3	71.0	3.59, m	2	1, 3, OMe
OMe	58.4	3.19, s	-	3
NH	-	8.97	2	2, 3, *Dh-Ala*-1
N-Me-Val				
1	168.3	-	-	-
2	59.7	5.31, bd (8.6)	3	nd
3	27.8	2.36, m	2, 3‘, 4	4, nd
3‘	18.3	0.38, d (6.4)	4	2, 3, 4
4	20.1	0.71, d (6.4)	3‘	2, 3, 3‘
NMe	30.2	2.90, s	-	2, *Me-Ser*-1
Thiazole				
1	160.5	-	-	-
2	145.2	-	-	-
3	130.6	8.57, s	-	1, 2, *N-Me-Val*-1

* overlapping signals circumvent exact assignment

nd = not detectable

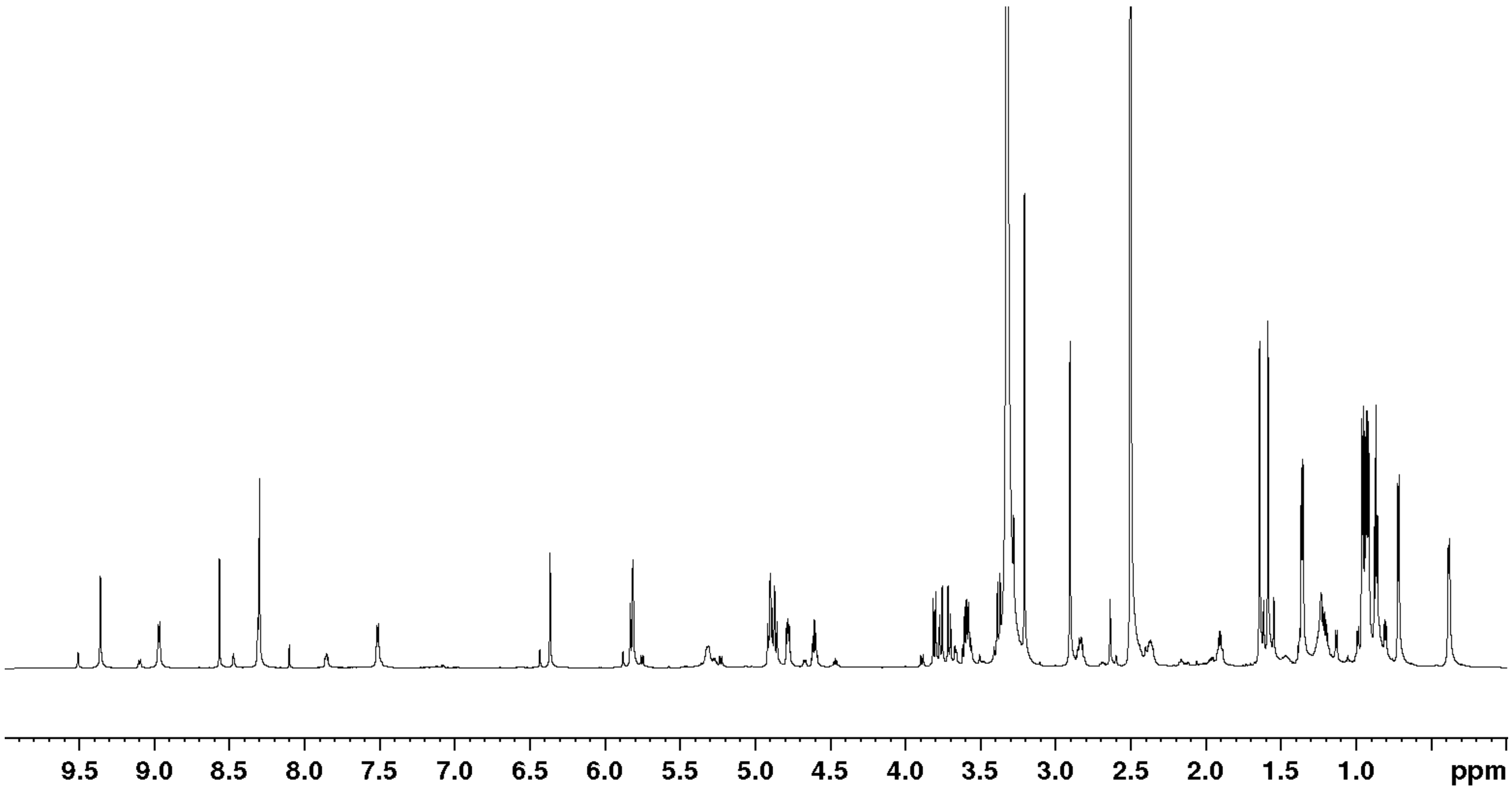

Figure 26 ^{1}H spectrum of thiamyxin B in DMSO-d_6 at 700 MHz.

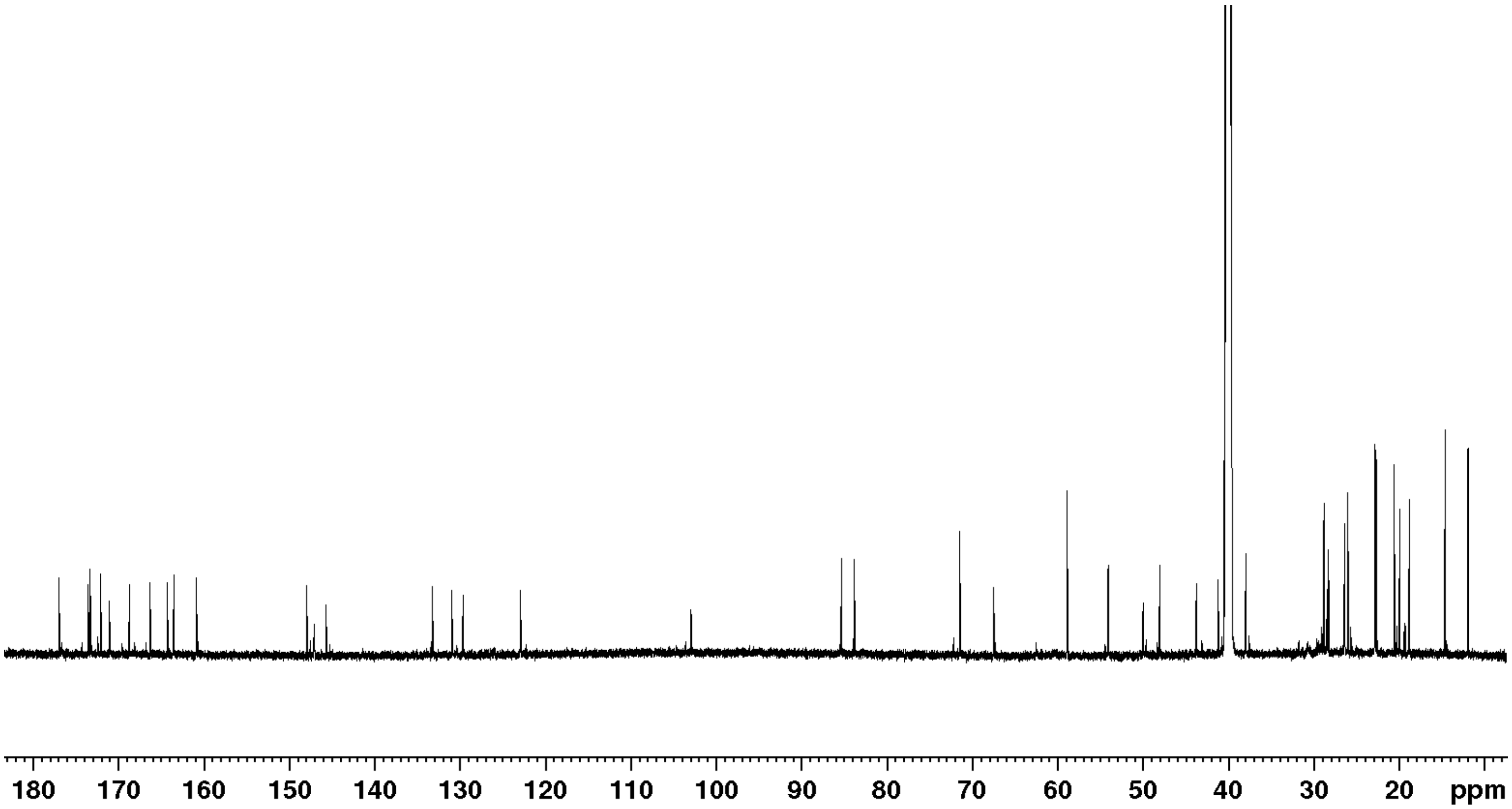

Figure 27 ^{13}C spectrum of thiamyxin B in DMSO-d_6 at 175 MHz.

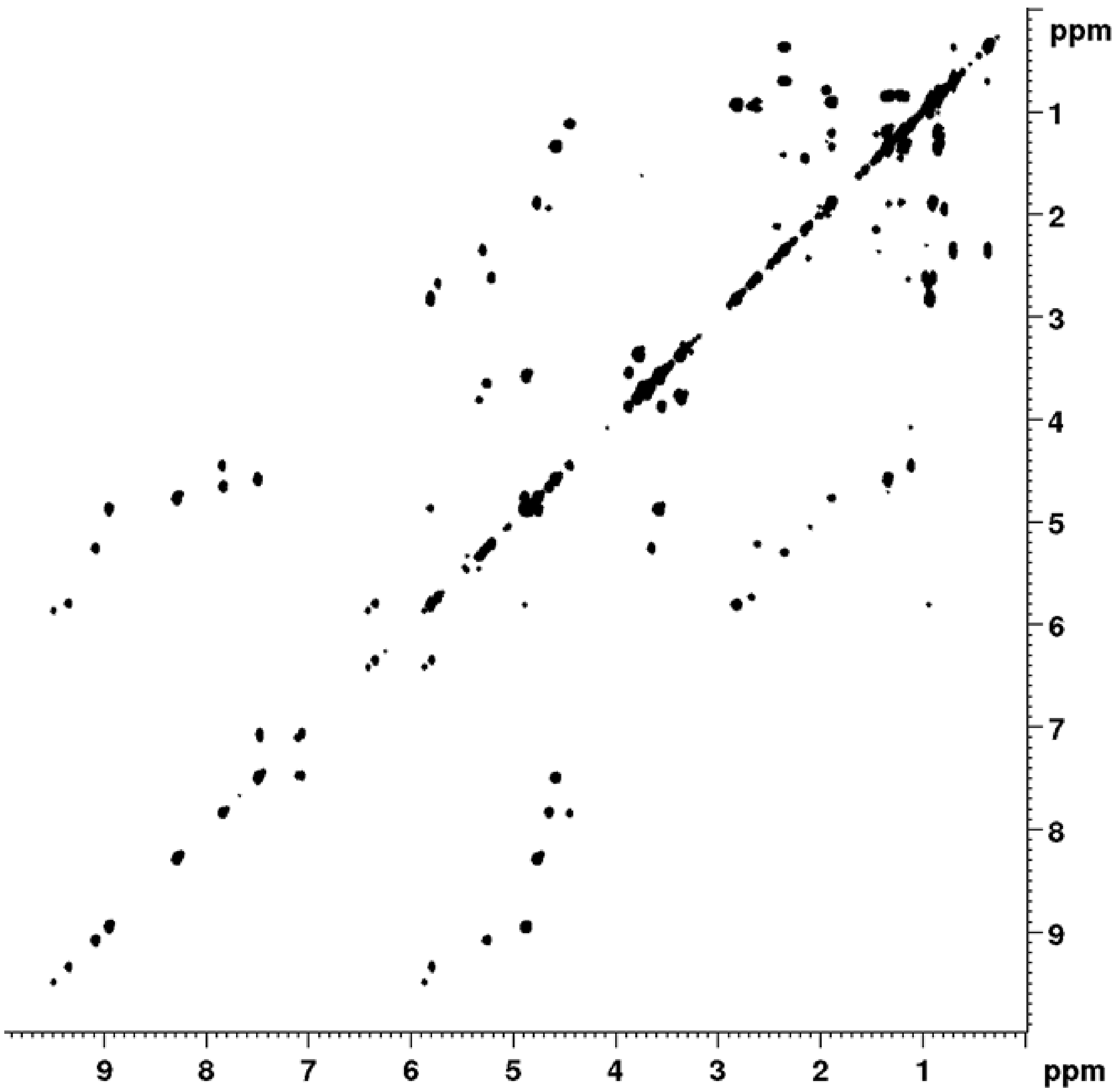

Figure 28 COSY spectrum of thiamyxin B in DMSO-d_6 at 700 MHz.

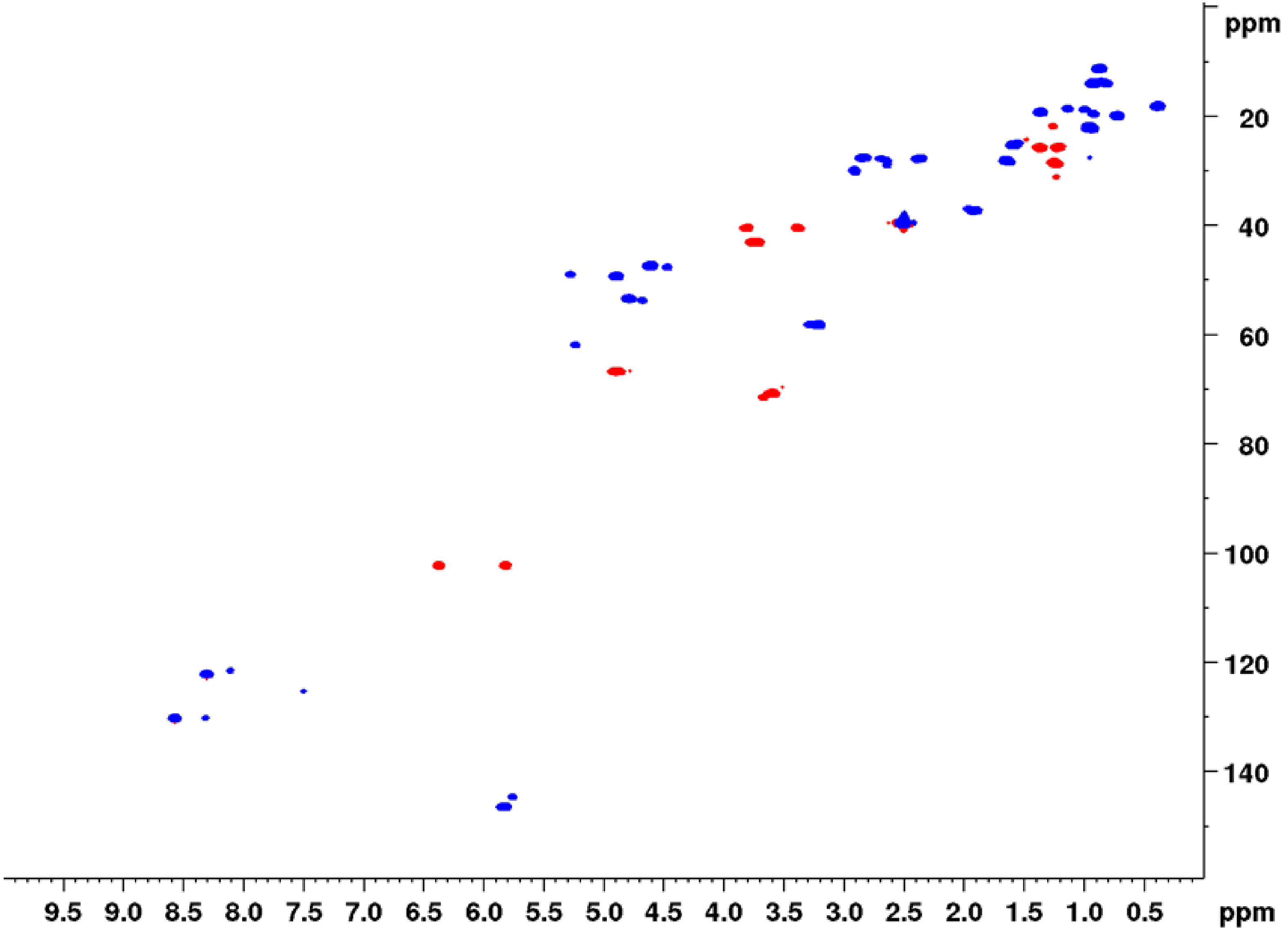

Figure 29 HSQC spectrum of thiamyxin B in DMSO-d_6 at 700/175 MHz.

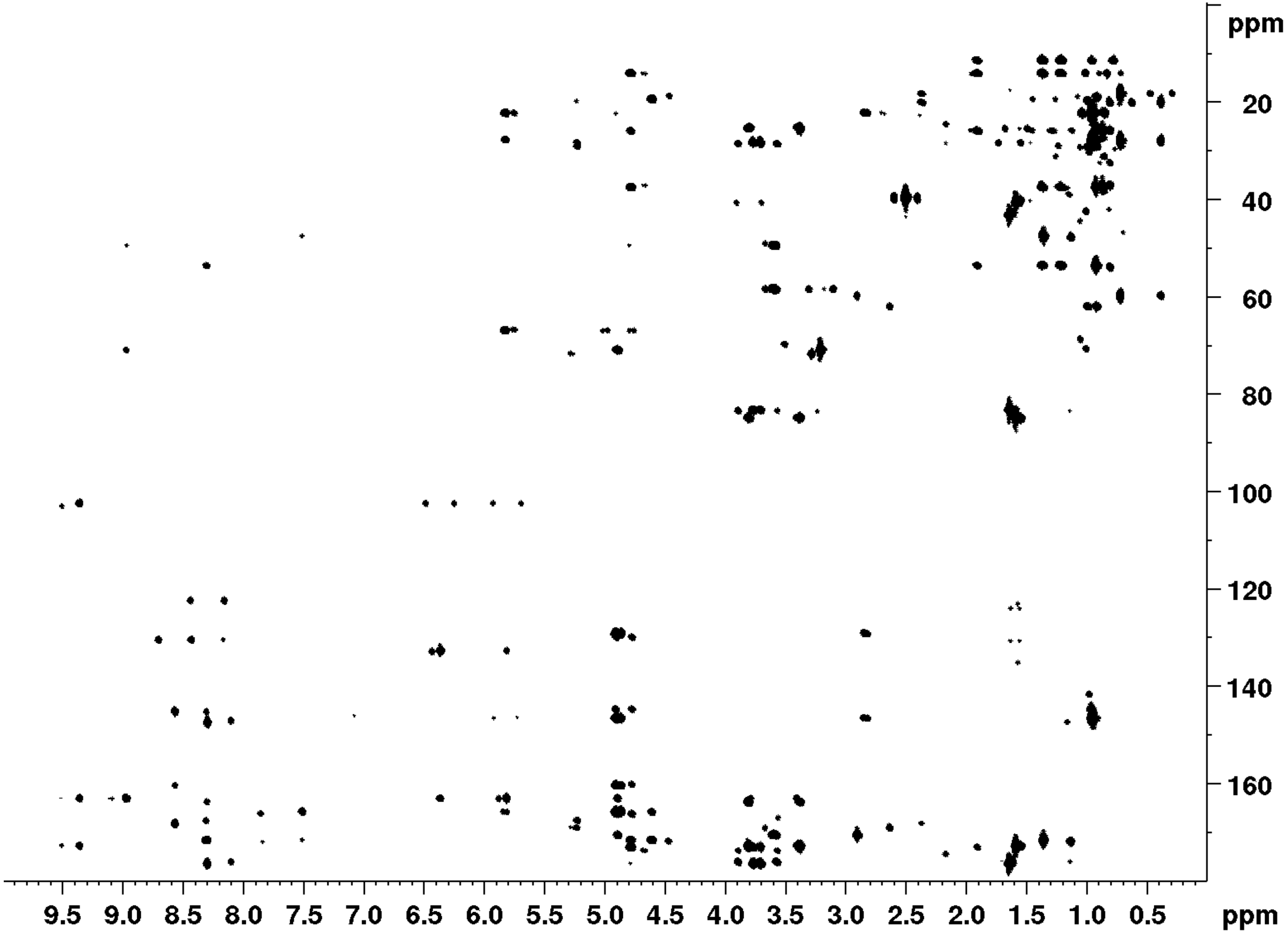

Figure 30 HMBC spectrum of thiamyxin B in DMSO-d_6 at 700/175 MHz.

2.8.2 Thiamyxin A

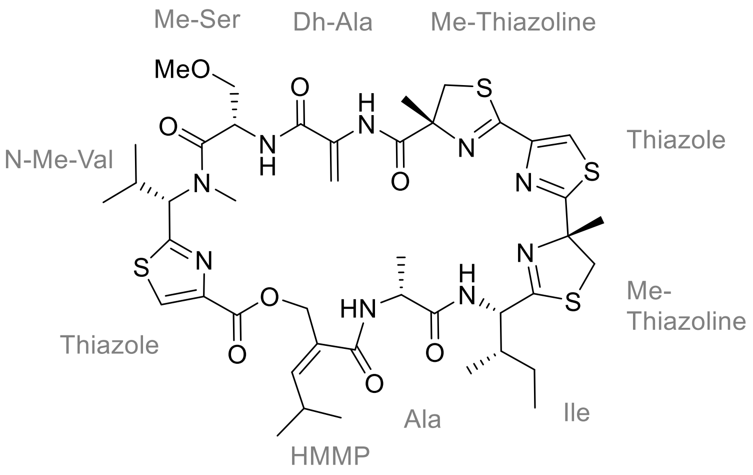

Table 6 NMR spectroscopic data of thiamyxin A in DMSO-d_6 at 500/125 MHz.

	NMR data in DMSO-d_6			
position	δ_C	δ_H (J in Hz)	COSY correlations	HMBC correlations
HMMP				
1	165.6	-	-	-
2	129.8	-	-	-
2’	65.9	4.81, 4.92, d (12.8)	3	1, 2, 3, *Thiazole*-1
3	142.6	5.76, d (10.0)	2’, 4	1, 2, 2’,4, 4’, 5
4	27.6	2.70, m	3, 4’, 5	2, 3, 4’, 5
4’	22.4	0.92, m*	4, 5	3, 4, 5
5	22.4	0.92, m*	4, 4’	3, 4, 4’
Ala				
1	171.6	-	-	-
2	48.4	4.42, quin (6.8)	3, NH	1, 3, *HMMP* -1
3	19.0	1.20, bd (7.0)	2	1, 2
NH	-	7.83, d (8.0)	2	-
Ile				
1	174.6	-	-	-

2	55.8	4.64, dd (8.8, 8.8)	3, NH	1, 3, 3', 4, *Ala*-1
3	37.0	1.82, m	2, 3', 4	1, 2, 3', 4, 5
3'	15.1	0.91, dd (10.5, 6.8)	3	2, 3, 4, 5
4	24.3	1.49, 1.19, m*	3, 5	3' ,3 ,5
5	10.4	0.85, t (7.4)	4	3, 4
NH	-	8.26, d (8.8)	2	2, *Ala*-1
Me-thiazoline				
1	176.1	-	-	-
2	82.8	-	-	-
3	43.4	3.63, 3.50, m*	-	1, 2, Me, *Ile*-1
Me	27.2	1.67, s	-	1, 2, 3
Thiazole				
1	163.7	-	-	-
2	147.9	-	-	-
3	121.9	8.26, s*	-	1, 2, *Me-thiazoline*-1
Me-thiazoline				
1	*172.8*	-	-	-
2	*84.8*	-	-	-
3	*40.6*	*3.37, 3.78, d (11.6)*	-	*1, 2, Me*, Thiazole-1
Me	*25.2*	*1.56, s*	-	*1, 2, 3*
Dh-Ala				
1	162.9	-	-	-
2	132.9	-	-	-
3	102.5	6.38, 5.79, s	NH	1, 2
NH	-	9.28, s	3	1, 3, *Me-Thiazoline-1*
Me-Ser				

1	170.6	-	-	-
2	49.4	4.88, m*	3, NH	1, 3, *Dh-Ala*-1
3	70.8	3.58, m	2	1, 3, OMe
OMe	58.3	3.20, s	-	3
NH	-	8.93	2	2, 3, *Dh-Ala*-1
N-Me-Val				
1	168.1	-	-	-
2	61.7	5.05, bd (10.6)	3	1, 3, 3‘, 4
3	27.8	2.31, m	2, 3‘, 4	1, 3‘, 4
3‘	18.3	0.54, d (6.6)	4	2, 3, 4
4	19.7	0.74, d (6.4)	3‘	2, 3, 3‘
NMe	30.0	2.82, s	-	2, *Me-Ser*-1
Thiazole				
1	160.4	-	-	-
2	144.9	-	-	-
3	130.3	8.54, s	-	1, 2, *N-Me-Val*-1

* overlapping signals circumvent exact assignment

nd = not detectable

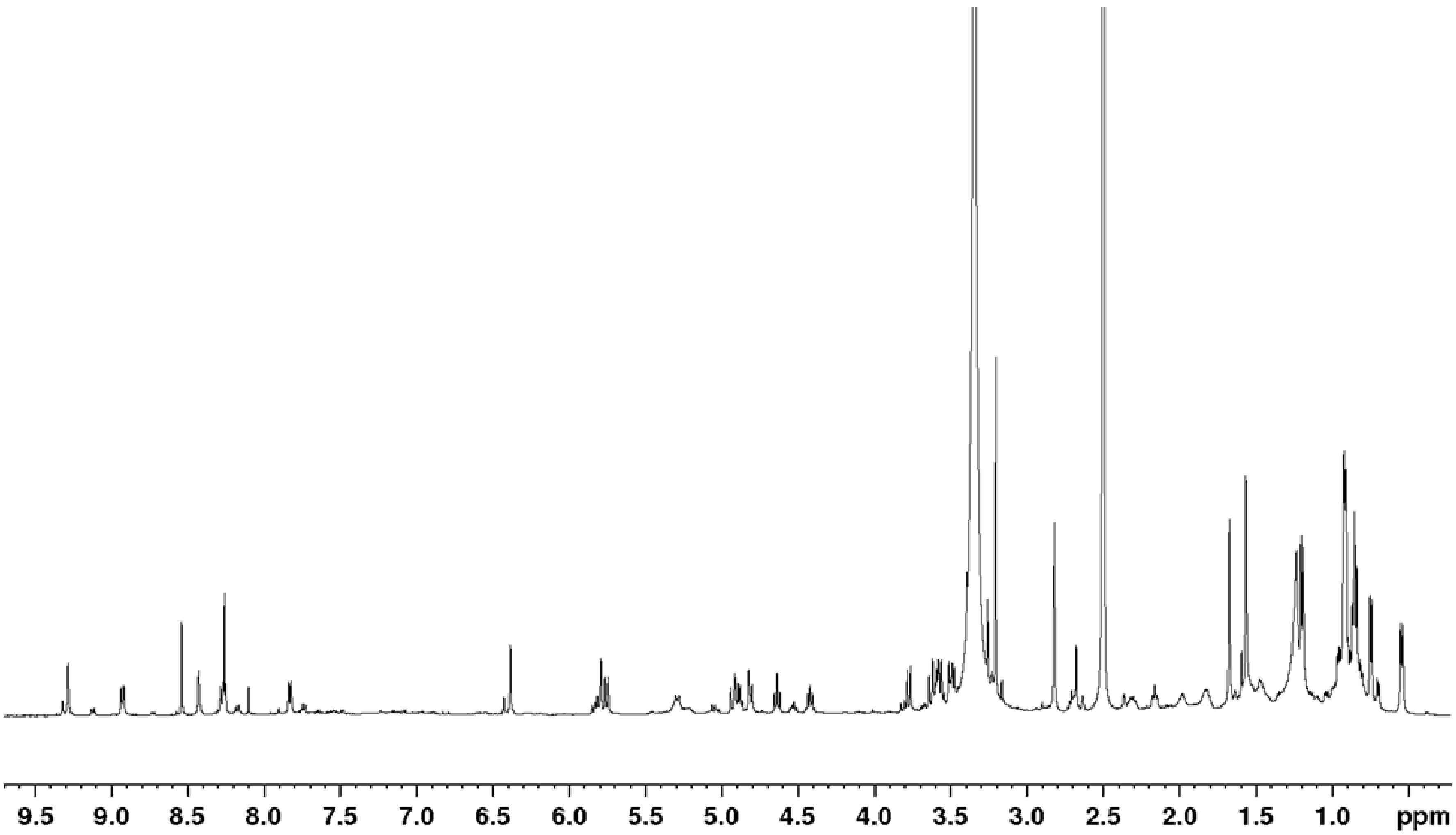

Figure 31 ^{1}H spectrum of thiamyxin A in DMSO-d_6 at 500 MHz.

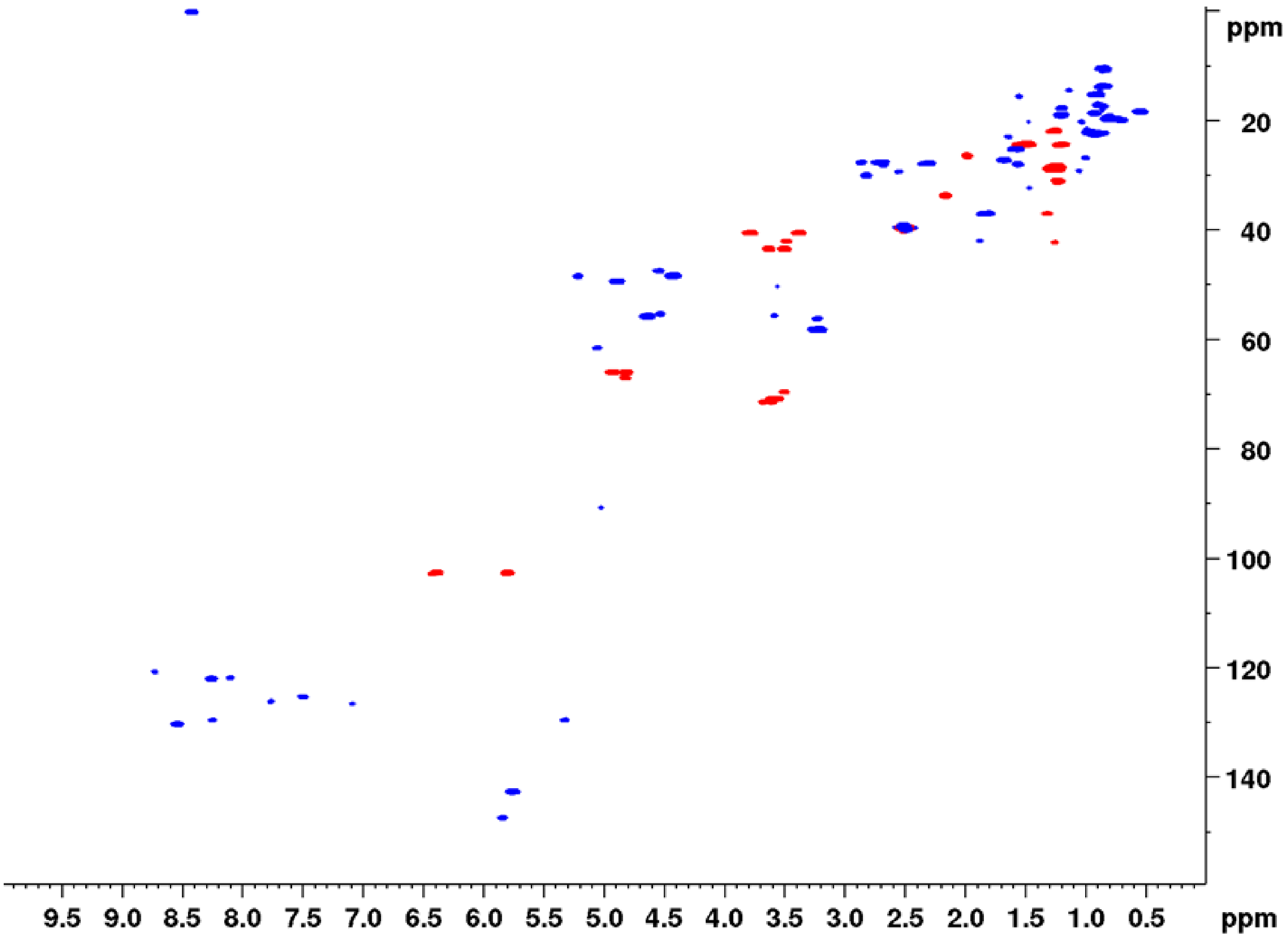

Figure 32 HSQC spectrum of thiamyxin A in DMSO-d_6 at 500/125 MHz.

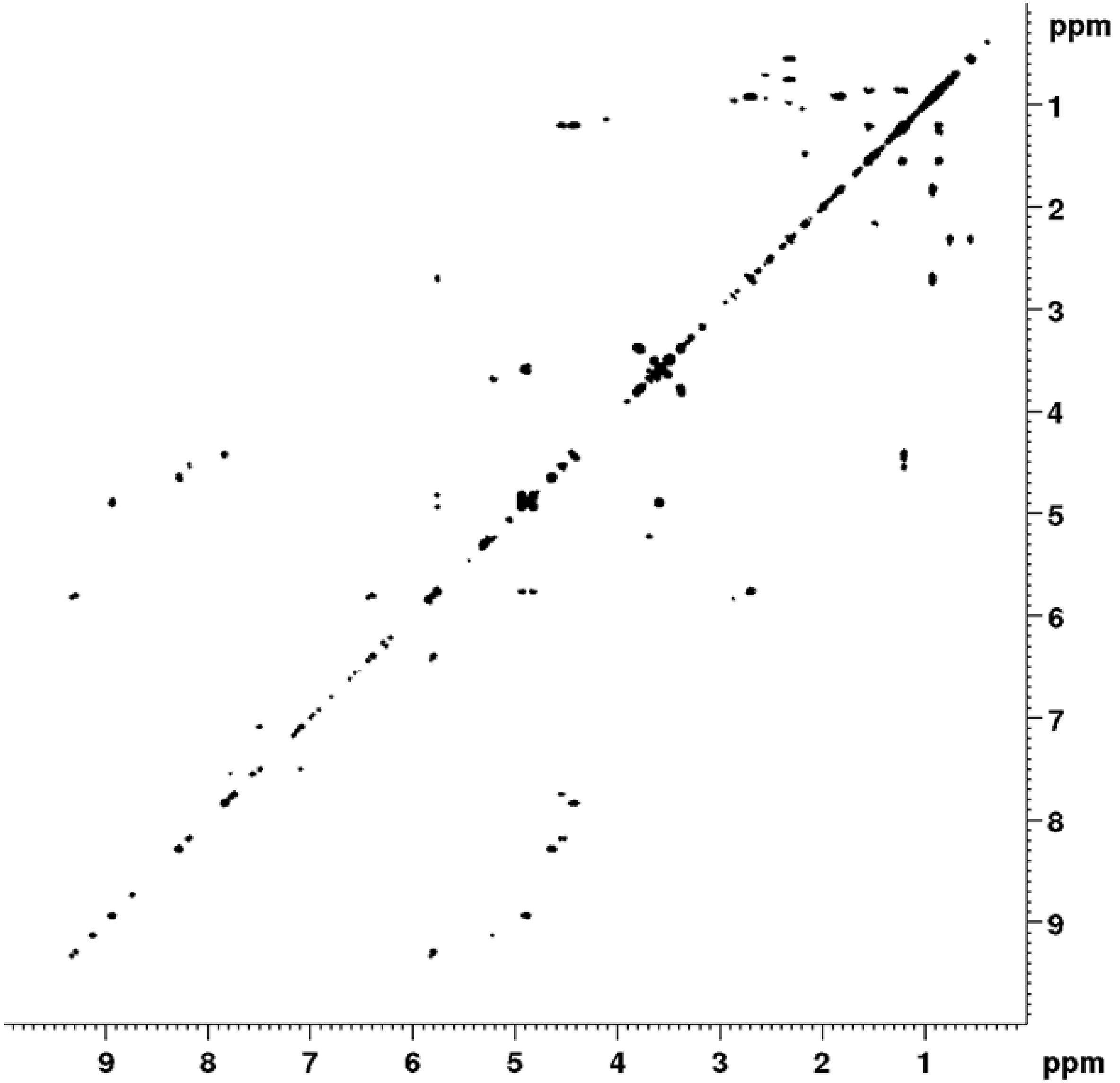

Figure 33 COSY spectrum of thiamyxin A in DMSO-d_6 at 500 MHz.

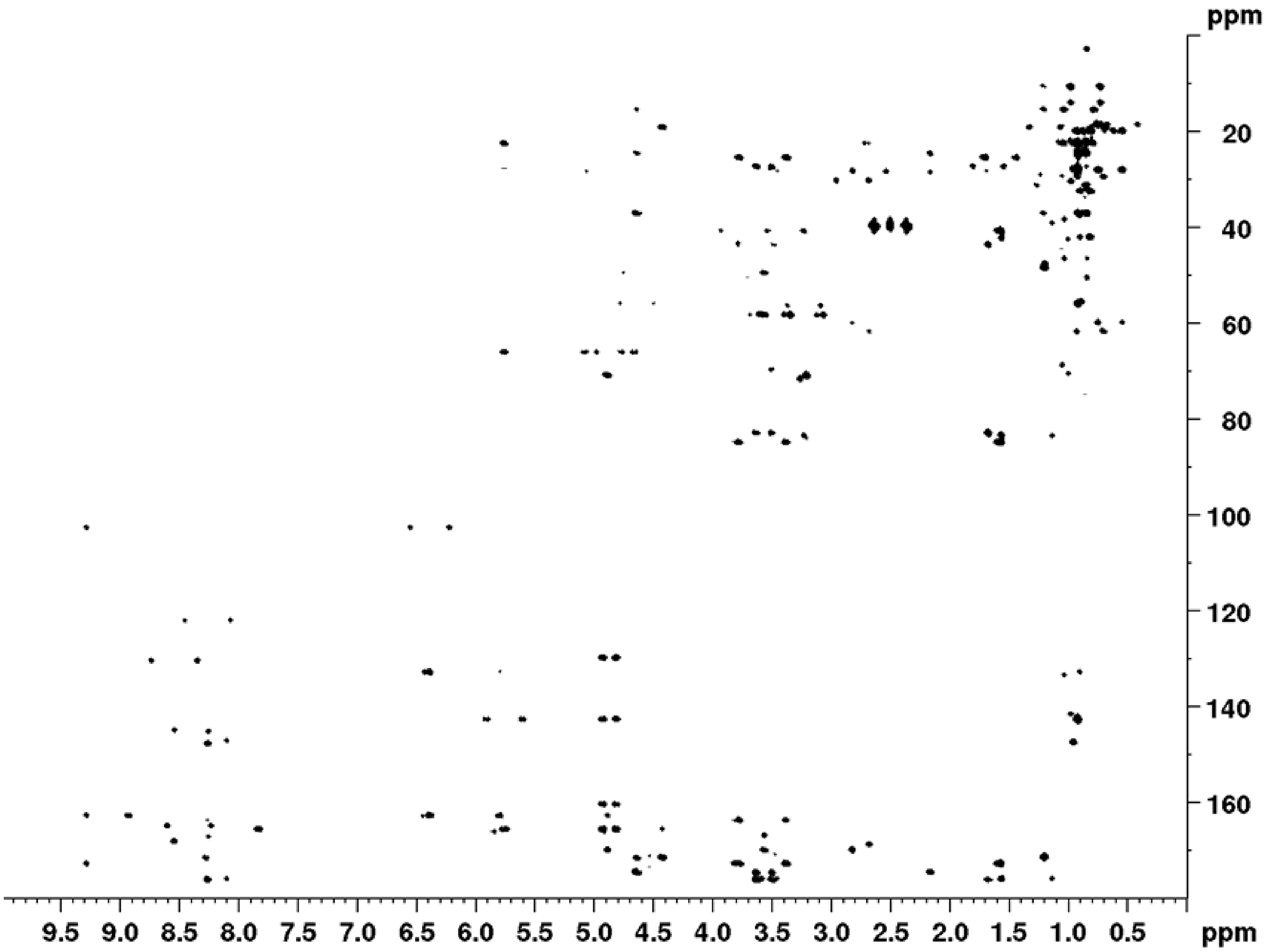

Figure 34 HMBC spectrum of thiamyxin A in DMSO-d_6 at 500/125 MHz.

2.8.3 Thiamyxin C

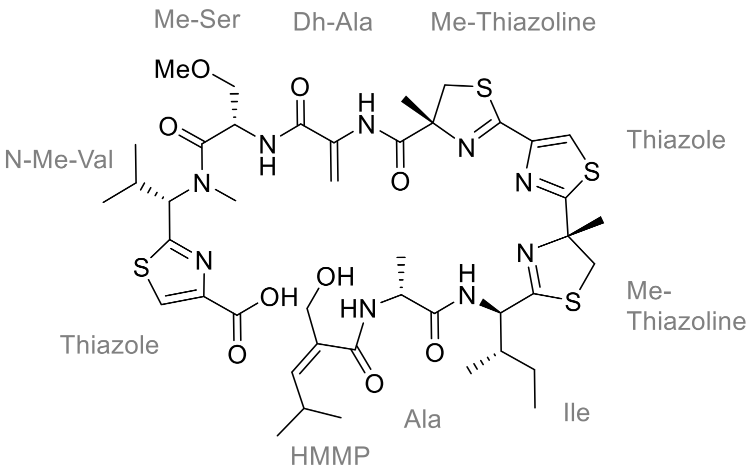

Table 7 NMR spectroscopic data of thiamyxin C in DMSO-d_6 at 700/175 MHz.

	NMR data in DMSO-d_6[xx]			
position	δ_C	δ_H (J in Hz)	COSY correlations	HMBC correlations
HMMP				
1	167.6	-	-	-
2	134.6	-	-	-
2’	63.1	4.00, m	3	1, 2, 3
3	139.8	5.43, bd (9.8)	2’, 4	1, 2, 2’,4, 4’, 5
4	27.3	2.81, m	3, 4’, 5	2, 3, 4’, 5
4’	22.7	0.90, m*	4, 5	3, 4, 5
5	22.7	0.90, m*	4, 4’	3, 4, 4’
Ala				
1	172.8	-	-	-
2	47.9	4.52, quin (7.3)	3, NH	1, 3, *HMMP* -1
3	18.4	1.30, q (7.4)	2	1, 2
NH	-	7.98, bs	2	-
Ile				
1	175.5	-	-	-

2	54.2	4.77, dd (8.9, 4.9)	3, NH	1, 3, 3’, 4, *Ala*-1
3	37.7	2.01, m	2, 3’, 4	2, 3’, 4, 5
3’	14.4	0.92, m*	3	2, 3, 4, 5
4	25.7	1.38, 1.20, m*	3, 5	3‘ ,3 ,5
5	11.4	0.85, t (7.8)	4	3, 4
NH	-	8.27, bd (8.8)	2	*Ala*-1
Me-thiazoline				
1	176.7	-	-	-
2	83.7	-	-	-
3	43.1	3.62, 3.51, m*	-	1, 2, Me, *Ile*-1
Me	27.4	1.64, s	-	1, 2, 3
Thiazole				
1	163.1	-	-	-
2	147.7	-	-	-
3	122.2	8.21, s*	-	2, *Me-thiazoline*-1
Me-thiazoline				
1	*172.8*	-	-	-
2	*85.0*	-	-	-
3	*40.4*	*3.39, 3.76, m**	-	*1, 2, Me,* Thiazole-1
Me	*25.9*	*1.54, s*	-	*1, 2, 3*
Dh-Ala				
1	163.1	-	-	-
2	133.2	-	-	-
3	103.0	6.37, 5.79, s	NH	1, 2
NH	-	9.30, s	3	1, 3, *Me-Thiazoline-1*
Me-Ser				

1	170.0	-	-	-
2	50.0	4.89, m*	3, NH	1, 3, *Dh-Ala*-1
3	70.8	3.56, m	2	1, 3, OMe
OMe	58.3	3.19, s	-	3
NH	-	8.85, t (7.8)	2	3, *Dh-Ala*-1
N-Me-Val				
1	172.2	-	-	-
2	59.8	5.36, bd (10.6)	3	n.d.
3	27.9	2.46, m	2, 3‘, 4	n.d.
3‘	18.9	0.72, t (7.8)	4	2, 3, 4
4	20.0	0.82, m*	3‘	2, 3, 3‘
NMe	29.8	2.92, s	-	2, *Me-Ser*-1
Thiazole				
1	176.4	-	-	-
2	147.7	-	-	-
3	122.3	8.19, s	-	1, 2

[xx] main diastereoisomer (D-*allo*-Ile carrying derivative) was analyzed

* overlapping signals circumvent exact assignment

n.d. = not detectable

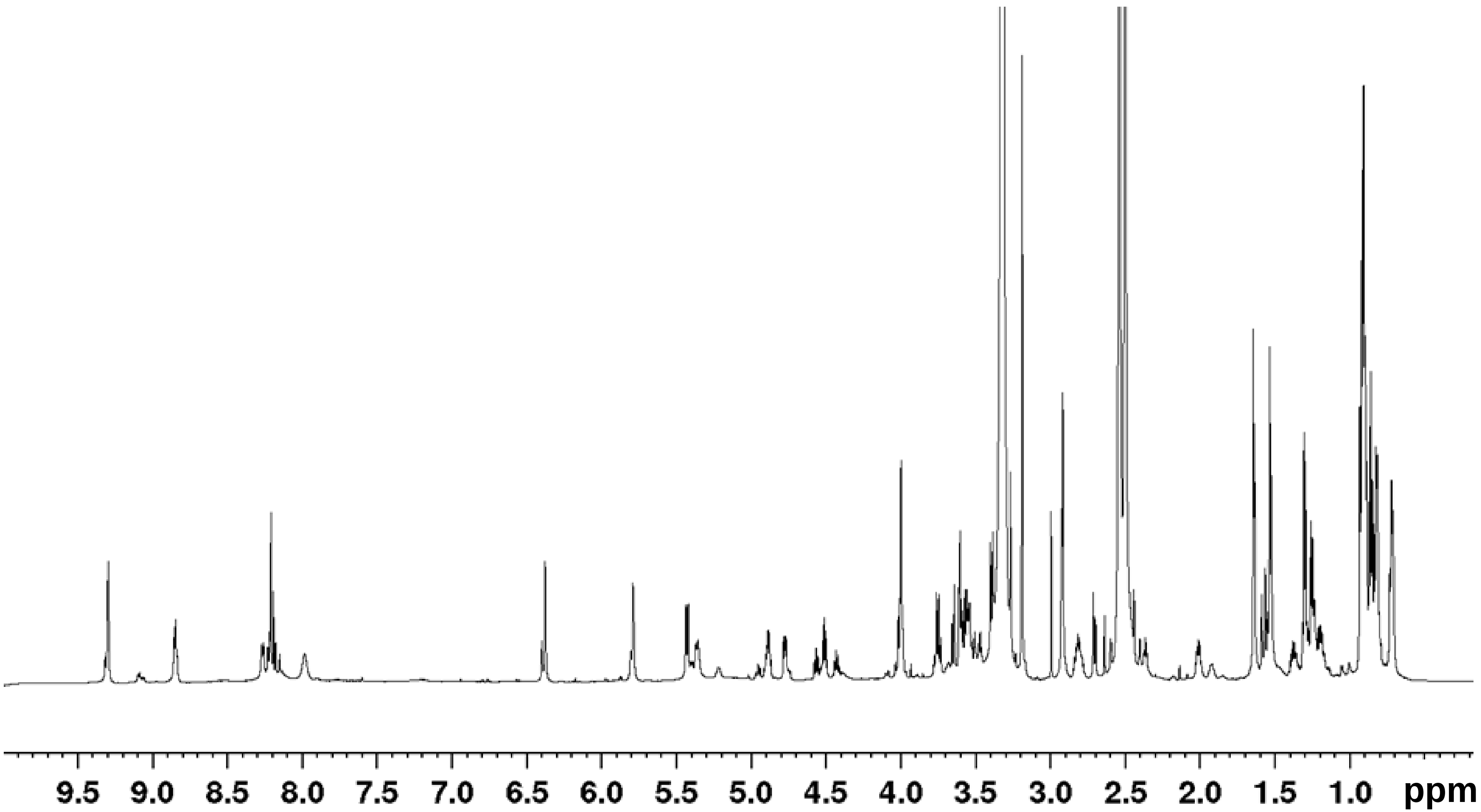

Figure 35 ^{1}H spectrum of thiamyxin C in DMSO-d_6 at 700 MHz.

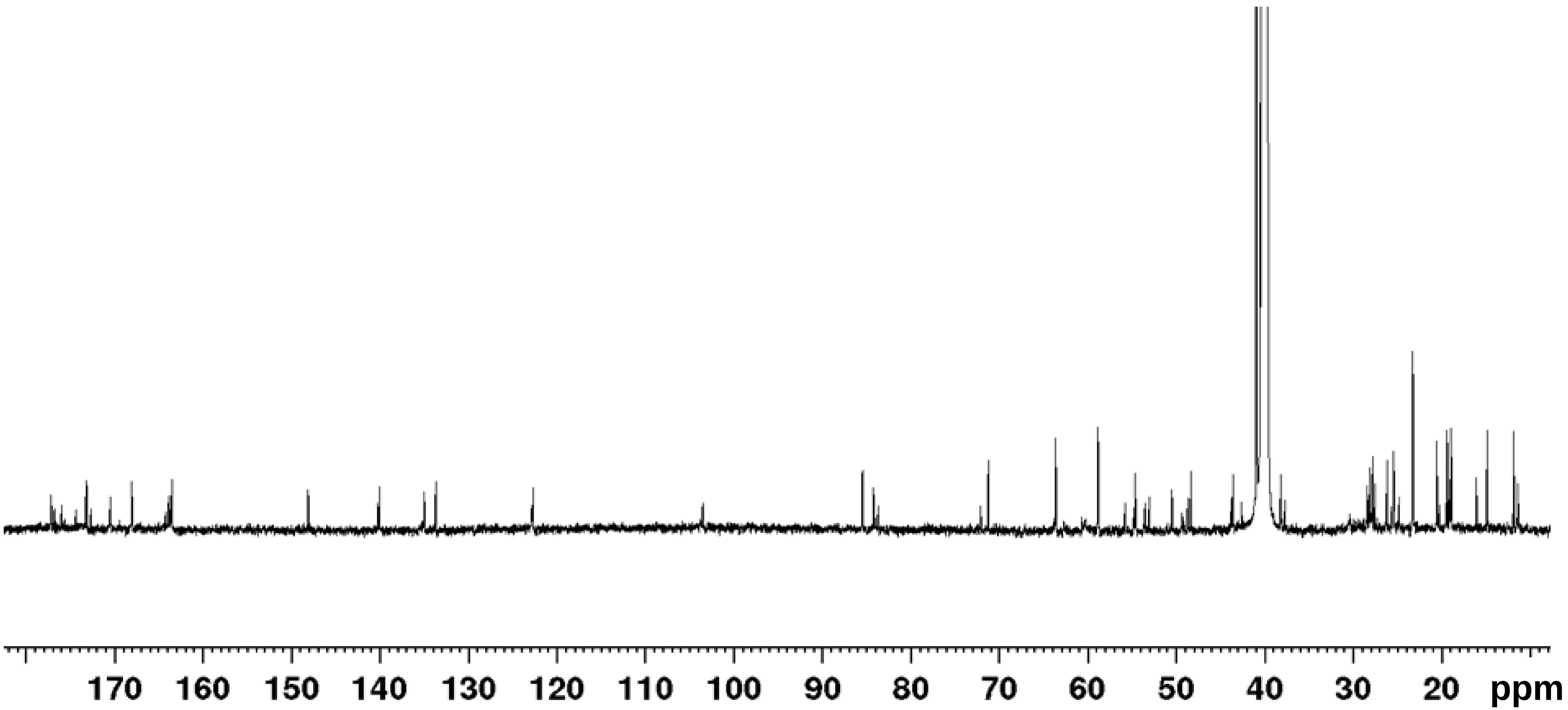

Figure 36 ^{13}C spectrum of thiamyxin C in DMSO-d_6 at 175 MHz.

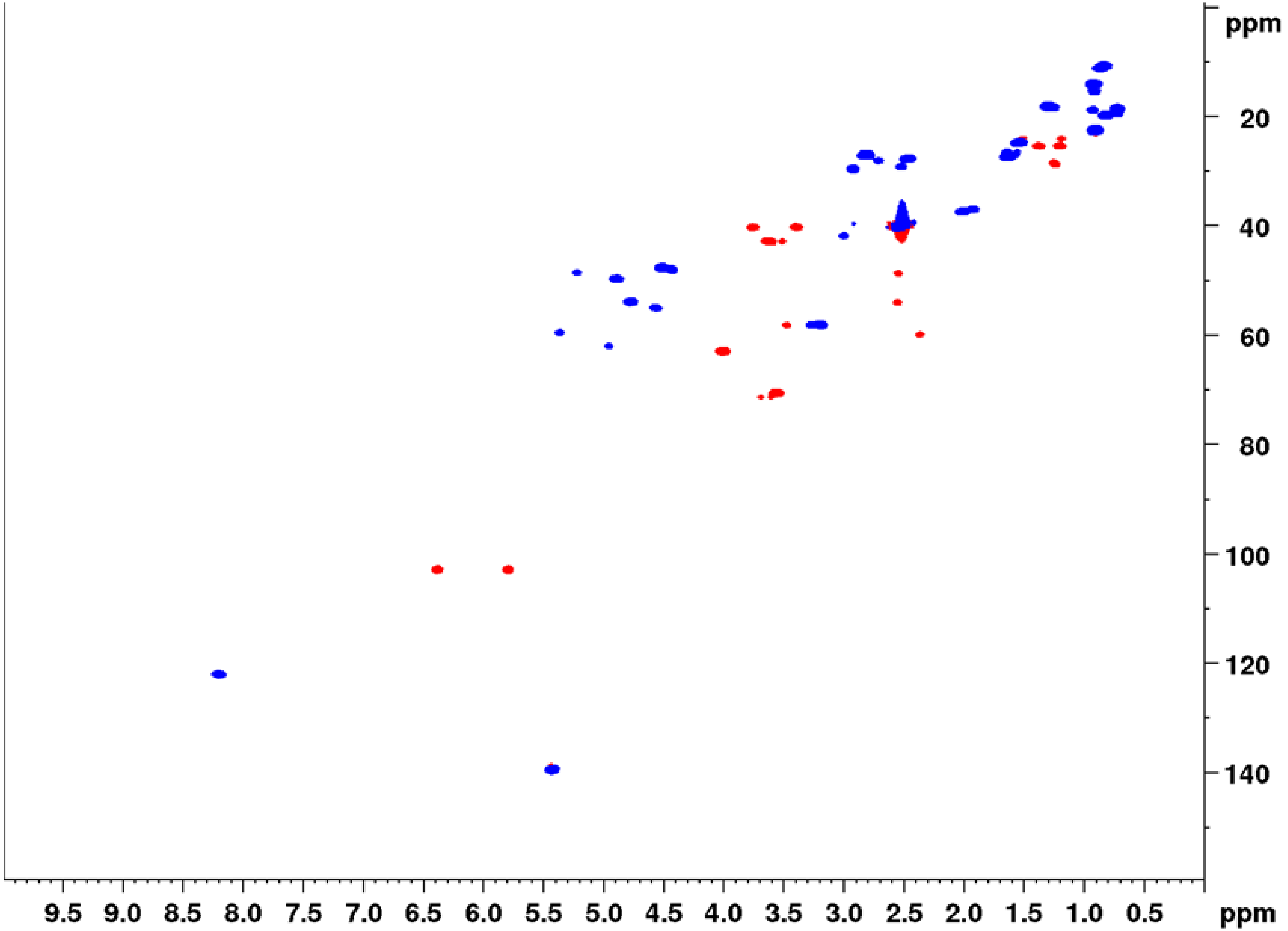

Figure 37 HSQC spectrum of thiamyxin C in DMSO-d_6 at 700/175 MHz.

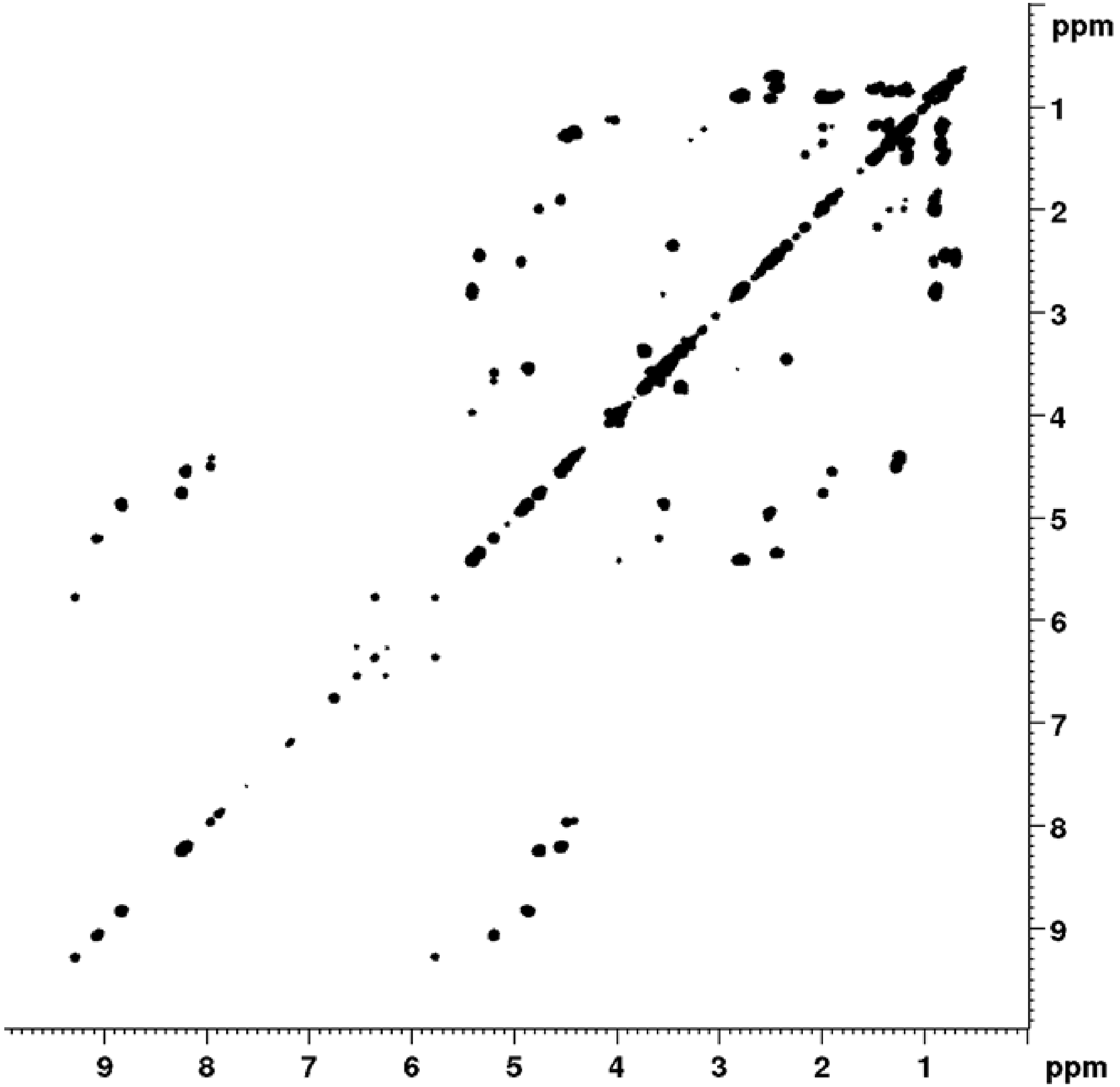

Figure 38 COSY spectrum of thiamyxin C in DMSO-d_6 at 700 MHz.

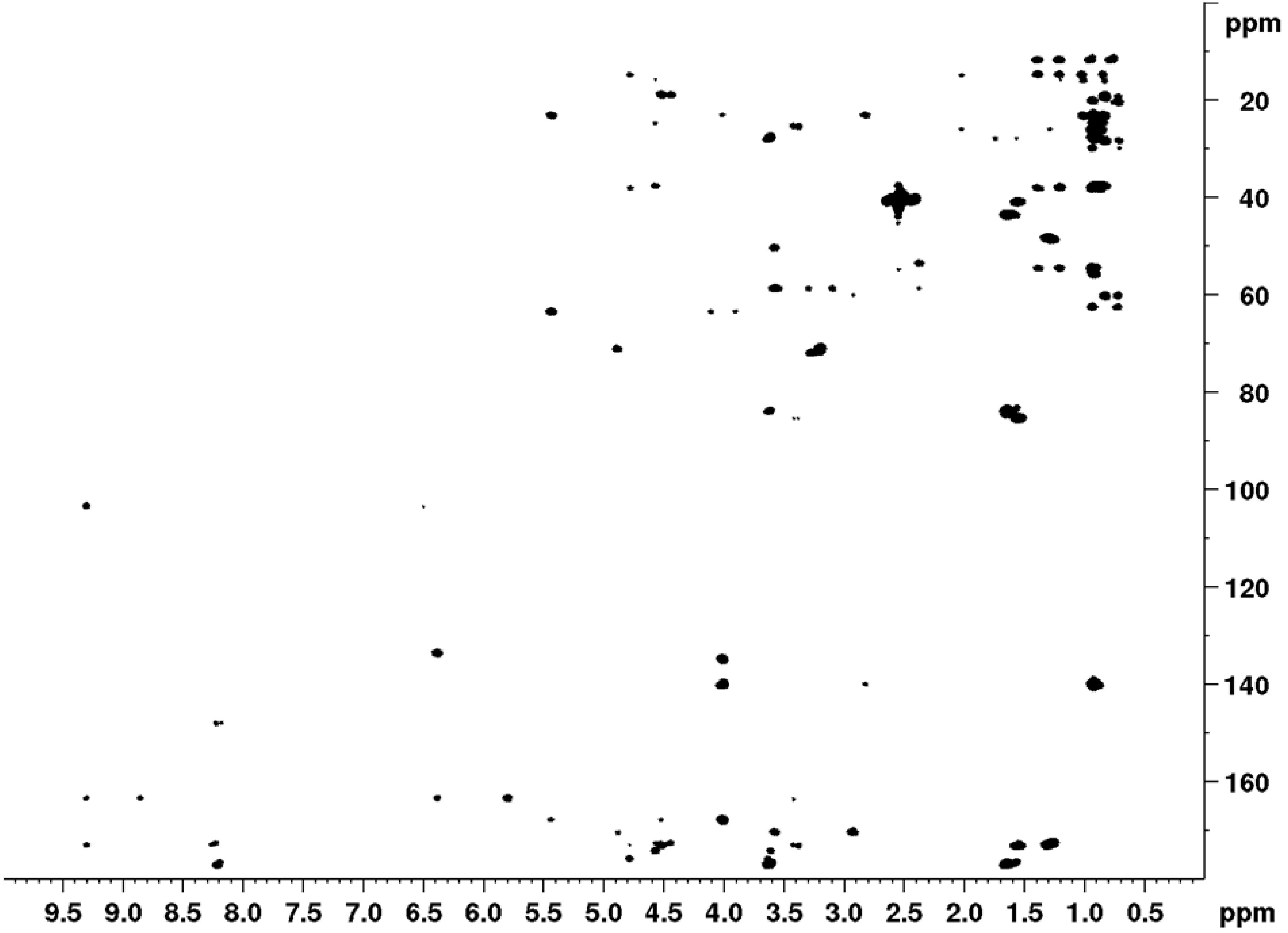

Figure 39 HMBC spectrum of thiamyxin C in DMSO-d_6 at 700/175 MHz.

2.8.4 Thiamyxin D

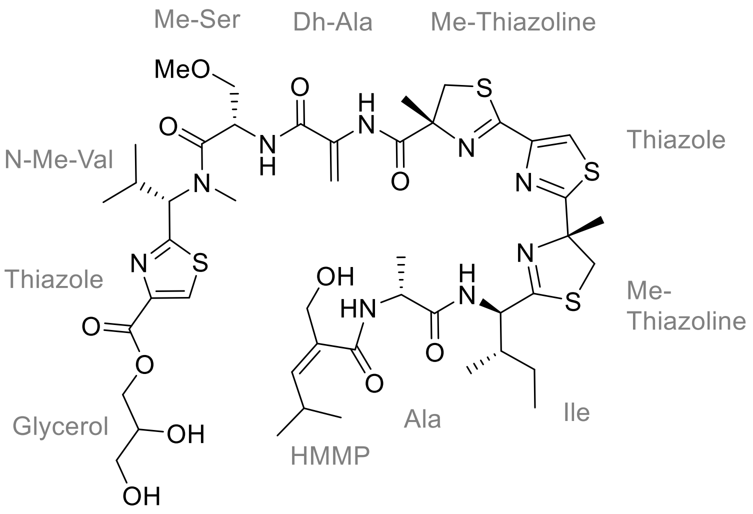

Table 8 NMR spectroscopic data of thiamyxin D in DMSO-d_6 at 700/175 MHz.

	NMR data in DMSO-d_6			
position	δ_C	δ_H (*J* in Hz)	COSY correlations	HMBC correlations
HMMP				
1	167.2	-	-	-
2	134.2	-	-	-
2’	63.0	4.00, s	3	1, 2, 3
3	139.5	5.45, bd (9.7)	2’, 4	1, 2, 2’,4, 4’, 5
4	27.0	2.83, m	3, 4’, 5	2, 3, 4’, 5
4’	22.4	0.91, m*	4, 5	3, 4, 5
5	22.4	0.91, m*	4, 4’	3, 4, 4’
Ala				
1	172.6	-	-	-
2	47.6	4.51, quin (7.3)	3, NH	1, 3, *HMMP*-1
3	18.0	1.29, m*	2	1, 2
NH	-	7.85, bd (7.4)	2	-
Ile				
1	175.0	-	-	-

2	53.8	4.76, m*	3, NH	1, 3, 3’, 4, *Ala*-1
3	37.4	2.00, m	2, 3’, 4	2, 3’, 4, 5
3’	16.4	0.90, m*	3	2, 3, 4, 5
4	25.4	1.37, 1.21, m*	3, 5	3‘ ,3 ,5
5	11.0	0.89, m*	4	3, 4
NH	-	8.21, m*	2	*Ala*-1
Me-thiazoline				
1	176.6	-	-	-
2	89.8	-	-	-
3	42.8	3.62, 3.51, m*	-	1, 2, Me, *Ile*-1
Me	22.1	1.14, s	-	1, 2, 3
Thiazole				
1	163.1	-	-	-
2	147.3	-	-	-
3	121.9	8.19, s*	-	2, *Me-thiazoline*-1
Me-thiazoline				
1	*172.6*	-	-	-
2	*84.8*	-	-	-
3	*40.4*	*3.40, 3.75, m**	-	*1, 2, Me,* Thiazole-1
Me	*24.7*	*1.07, s*	-	*1, 2, 3*
Dh-Ala				
1	162.7	-	-	-
2	132.9	-	-	-
3	102.8	6.38, 5.78, s	NH	1, 2
NH	-	9.30, s	3	1, 3, *Me-Thiazoline-1*
Me-Ser				

1	169.8	-	-	-
2	49.7	4.88, m*	3, NH	1, 3, *Dh-Ala*-1
3	70.7	3.49, 3.65, m	2	1, 3, OMe
OMe	58.0	3.19, s	-	3
NH	-	8.84, t (7.8)	2	3, *Dh-Ala*-1
N-Me-Val				
1	168.0	-	-	-
2	59.5	5.41, bd (10.8)	3	1, 3, 3‘, 4, NMe, *Me-Ser*-1
3	27.7	2.47, m*	2, 3‘, 4	1, 2, 3‘, 4
3‘	18.7	0.76, s	4	2, 3, 4
4	19.8	0.86, m*	3‘	2, 3, 3‘
NMe	29.8	2.94, s	-	1, 2, *Me-Ser*-1
Thiazole				
1	160.4	-	-	-
2	145.1	-	-	-
3	129.8	8.52, s	-	1, 2, *N-Me-Val*-1
Glycerol				
1	66.1	4.16, 4.28	2	2, 3, *Thiazole*-1
2	69.2	3.76	1, 3	1, 3
3	62.3	3.41	2	1, 2

* overlapping signals circumvent exact assignment

n.d. = not detectable

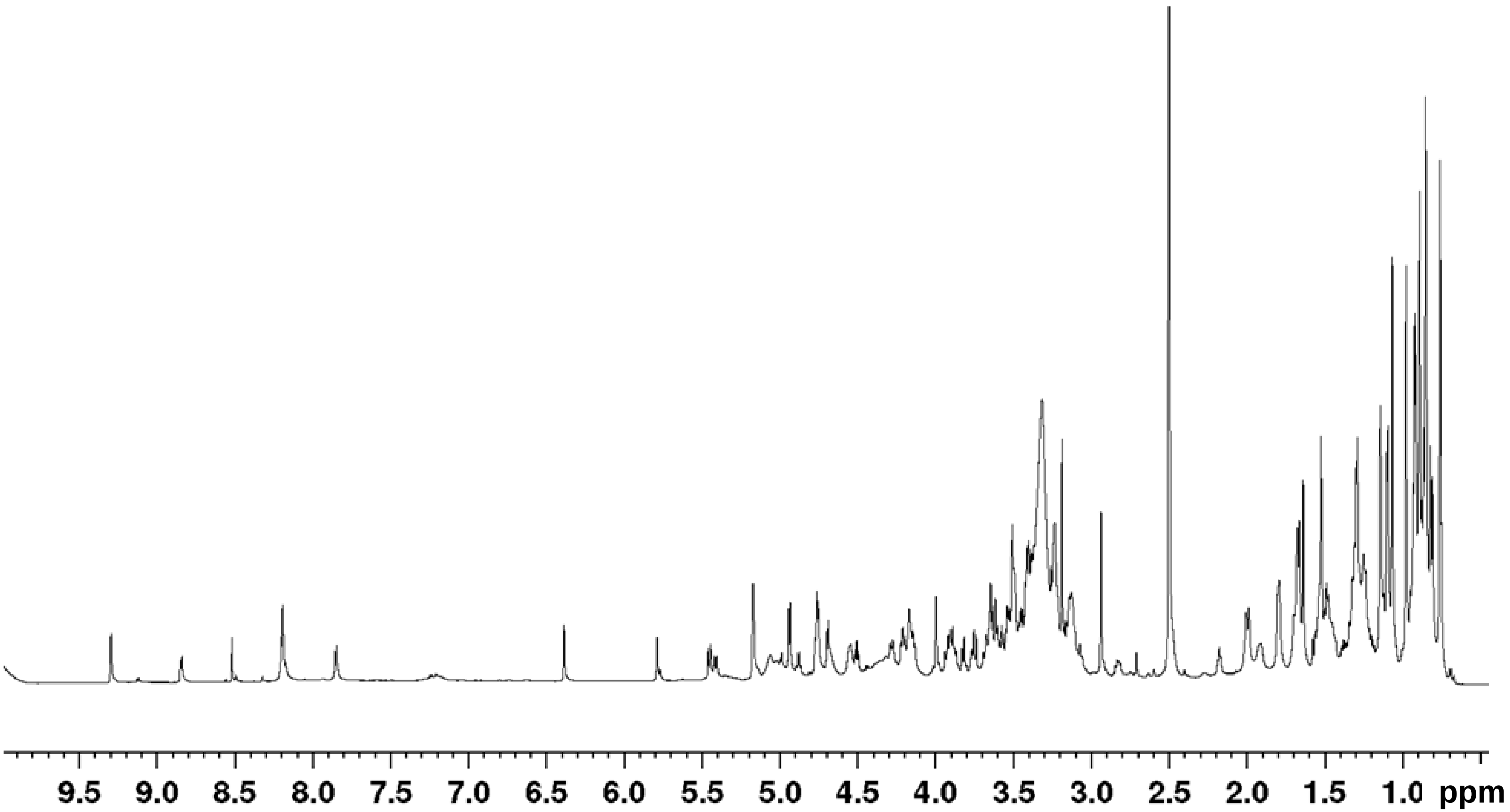

Figure 40 ^{1}H spectrum of thiamyxin D in DMSO-d_6 at 700 MHz.

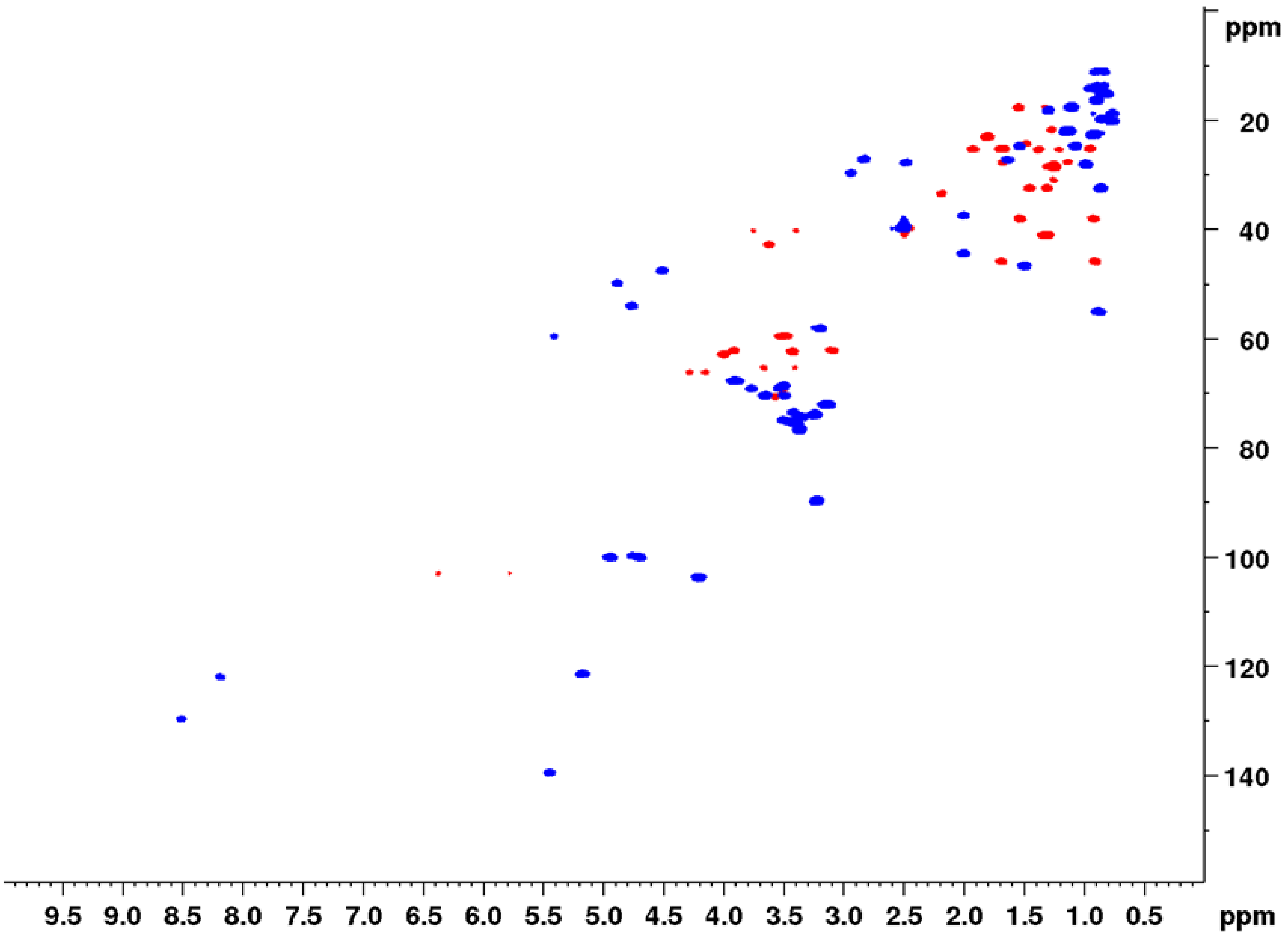

Figure 41 HSQC spectrum of thiamyxin D n DMSO-d_6 at 700/175 MHz.

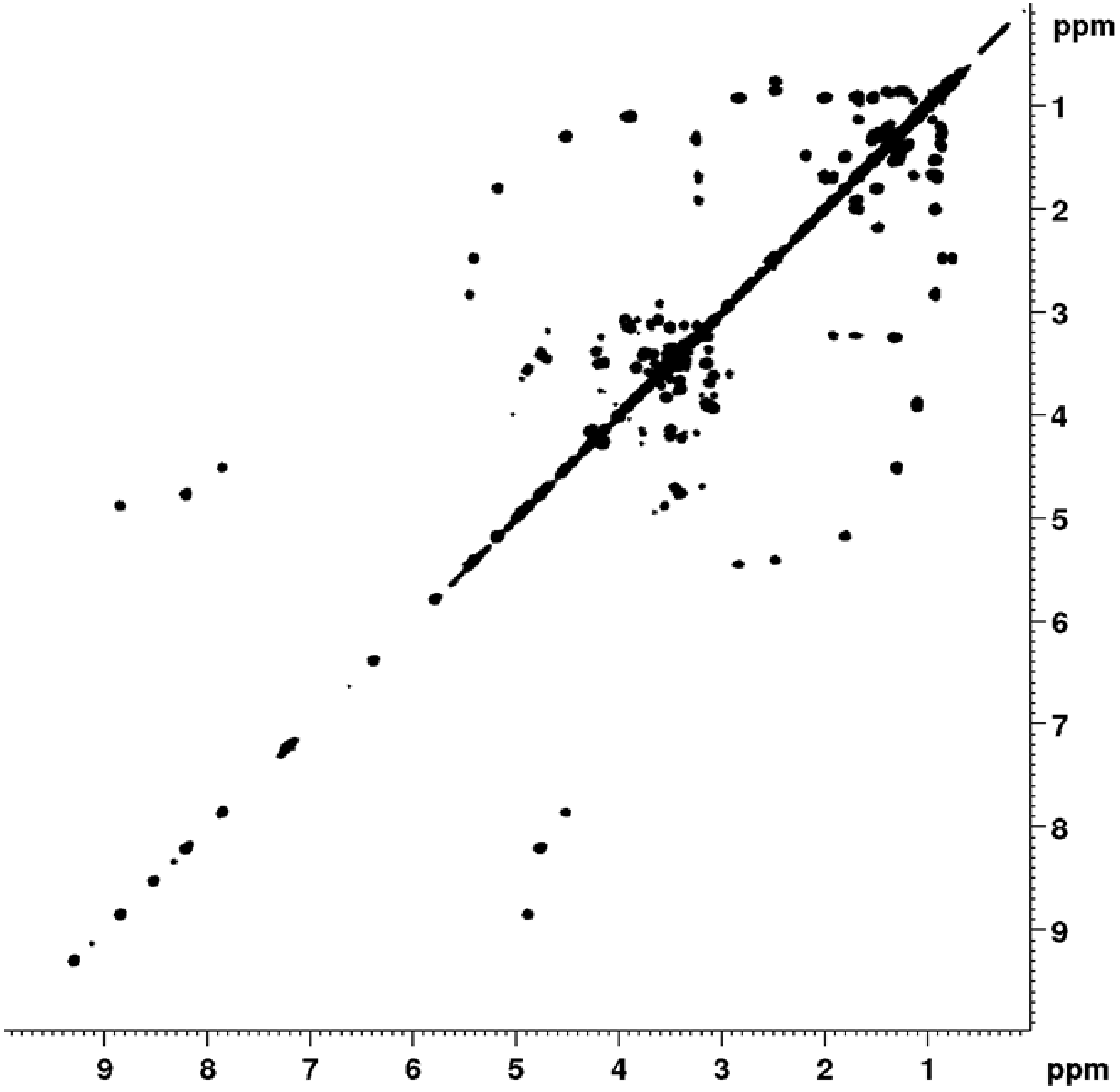

Figure 42 COSY spectrum of thiamyxin D in DMSO-d_6 at 700 MHz.

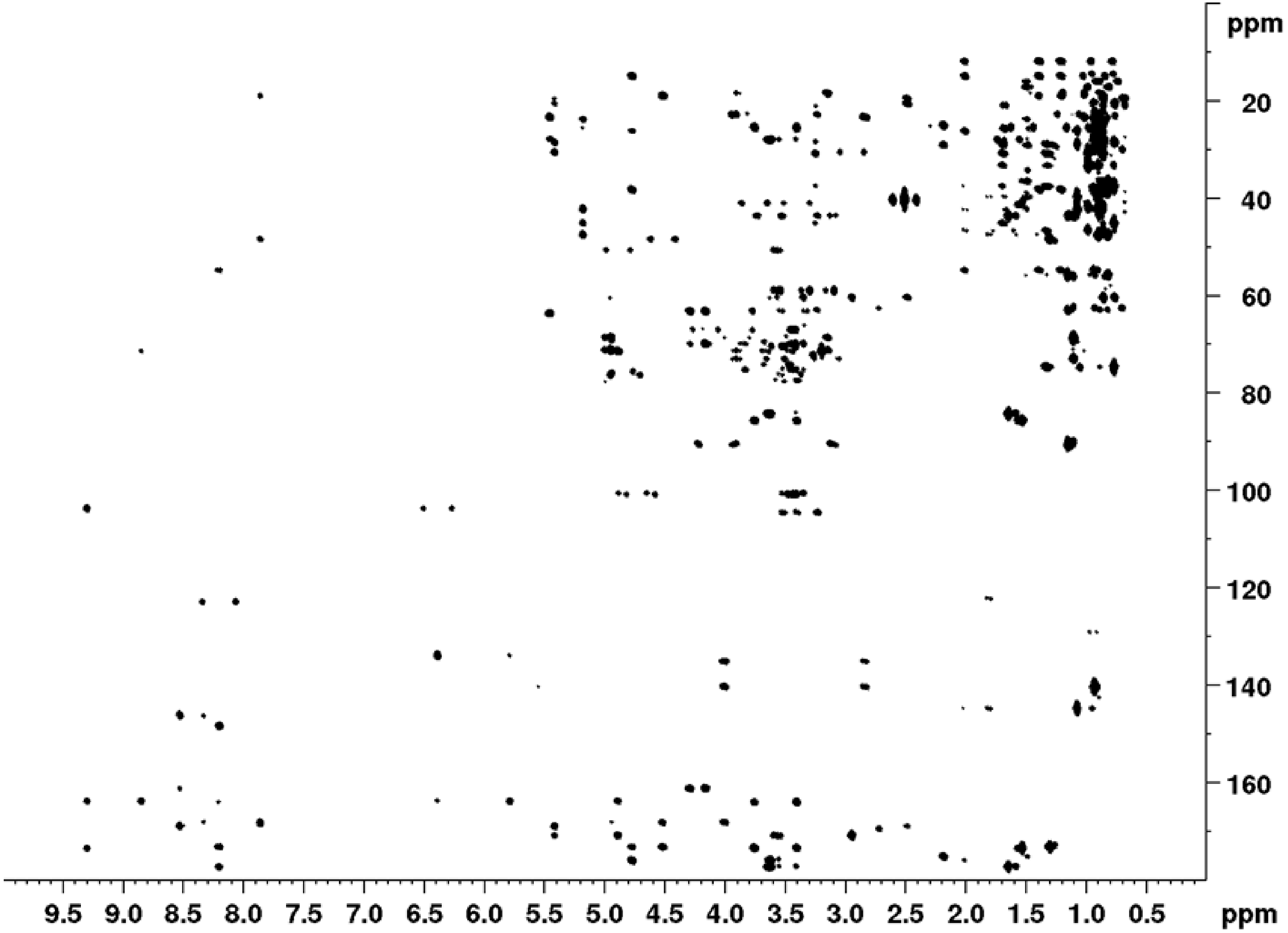

Figure 43 HMBC spectrum of thiamyxin D in DMSO-d_6 at 700/175 MHz.

3 References

[1] S. H. E. van den Worm, K. K. Eriksson, J. C. Zevenhoven, F. Weber, R. Züst, T. Kuri, R. Dijkman, G. Chang, S. G. Siddell, E. J. Snijder, V. Thiel, A. D. Davidson, *PLoS ONE* **2012**, *7*, e32857.

[2] C. D. Bader, F. Panter, R. Garcia, E. P. Tchesnokov, S. Haid, C. Walt, C. Spröer, A. F. Kiefer, M. Götte, J. Overmann, T. Pietschmann, R. Müller, *Chemistry – A European Journal* **2022**, *28*, e202104484.

[3] Okoth Dorothy A, J. J. Hug, R. Garcia, C. Spröer, J. Overmann, R. Müller, *Molecules (Basel, Switzerland)* **2020**, *25*, 2676.

[4] Z. J. Anderson, C. Hobson, R. Needley, L. Song, M. S. Perryman, P. Kerby, D. J. Fox, *Org. Biomol. Chem.* **2017**, *15*, 9372–9378.

[5] a) K. Fujii, Y. Ikai, T. Mayumi, H. Oka, M. Suzuki, K. Harada, *Anal. Chem.* **1997**, *69*, 3346–3352; b) R. Bhushan, H. Bruckner, *Amino Acids* **2004**, *27*, 231–247;

[6] P. Wipf, P. C. Fritch, *Tetrahedron Lett.* **1994**, *35*, 5397–5400.

[7] K. Blin, S. Shaw, K. Steinke, R. Villebro, N. Ziemert, S. Y. Lee, M. H. Medema, T. Weber, *Nucleic Acids Res.* **2019**, W81-W87.

[8] S. F. Altschul, W. Gish, W. Miller, E. W. Myers, D. J. Lipman, *J. Mol. Biol.* **1990**, *215*, 403–410.

[9] S. Lu, J. Wang, F. Chitsaz, M. K. Derbyshire, R. C. Geer, N. R. Gonzales, M. Gwadz, Di Hurwitz, G. H. Marchler, J. S. Song, N. Thanki, R. A. Yamashita, M. Yang, D. Zhang, C. Zheng, C. J. Lanczycki, A. Marchler-Bauer, *Nucleic Acids Res.* **2020**, *48*, D265-D268.

[10] M. A. Skiba, C. L. Tran, Q. Dan, A. P. Sikkema, Z. Klaver, W. H. Gerwick, D. H. Sherman, J. L. Smith, *Structure* **2020**, *28*, 63-74.e4.

[11] D. Pogorevc, Y. Tang, M. Hoffmann, G. Zipf, H. S. Bernauer, A. Popoff, H. Steinmetz, S. C. Wenzel, *ACS Synth. Biol.* **2019**, *8*, 1121–1133.

[12] C. Rausch, I. Hoof, T. Weber, W. Wohlleben, D. H. Huson, *BMC Evol. Biol.* **2007**, *7*, 78–92.
